# KIN‐4/MAST kinase promotes PTEN‐mediated longevity of *Caenorhabditis elegans* via binding through a PDZ domain

**DOI:** 10.1111/acel.12906

**Published:** 2019-02-17

**Authors:** Seon Woo A. An, Eun‐Seok Choi, Wooseon Hwang, Heehwa G. Son, Jae‐Seong Yang, Keunhee Seo, Hyun‐Jun Nam, Nhung T. H. Nguyen, Eun Ji E. Kim, Bo Kyoung Suh, Youngran Kim, Shunji Nakano, Youngjae Ryu, Chang Man Ha, Ikue Mori, Sang Ki Park, Joo‐Yeon Yoo, Sanguk Kim, Seung‐Jae V. Lee

**Affiliations:** ^1^ Department of Life Sciences Pohang University of Science and Technology Pohang Gyeongbuk South Korea; ^2^ School of Interdisciplinary Bioscience and Bioengineering Pohang University of Science and Technology Pohang Gyeongbuk South Korea; ^3^ Neuroscience Institute, Graduate School of Science Nagoya University Nagoya Japan; ^4^ Research Division Korea Brain Research Institute Daegu South Korea; ^5^ Present address: Research and Development D.R.NANO, Korea Institute of Science and Technology Seoul Korea; ^6^ Present address: Department of Chemical Engineering Pohang University of Science and Technology Pohang Gyeongbuk South Korea

**Keywords:** aging, DAF‐18/PTEN, insulin/IGF‐1 signaling, KIN‐4/MAST kinase, lifespan, PDZ

## Abstract

PDZ domain‐containing proteins (PDZ proteins) act as scaffolds for protein–protein interactions and are crucial for a variety of signal transduction processes. However, the role of PDZ proteins in organismal lifespan and aging remains poorly understood. Here, we demonstrate that KIN‐4, a PDZ domain‐containing microtubule‐associated serine‐threonine (MAST) protein kinase, is a key longevity factor acting through binding PTEN phosphatase in *Caenorhabditis elegans*. Through a targeted genetic screen for PDZ proteins, we find that *kin‐4* is required for the long lifespan of *daf‐2*/insulin/IGF‐1 receptor mutants. We then show that neurons are crucial tissues for the longevity‐promoting role of *kin‐4*. We find that the PDZ domain of KIN‐4 binds PTEN, a key factor for the longevity of *daf‐2* mutants. Moreover, the interaction between KIN‐4 and PTEN is essential for the extended lifespan of *daf‐2* mutants. As many aspects of lifespan regulation in *C. elegans* are evolutionarily conserved, MAST family kinases may regulate aging and/or age‐related diseases in mammals through their interaction with PTEN.

## 1 INTRODUCTION

Insulin/IGF‐1 signaling (IIS) is an evolutionarily conserved signaling pathway that regulates organismal lifespan. In *Caenorhabditis elegans*, mutations in the insulin/IGF‐1 receptor, *daf‐2*, substantially increase lifespan (Kenyon, 2010). Longevity conferred by *daf‐2* mutations requires various proteins, including DAF‐18/PTEN and transcription factors such as DAF‐16/FOXO, heat‐shock factor‐1 (HSF‐1), and SKN‐1/NRF2. The DAF‐18/PTEN protein dephosphorylates phosphatidylinositol‐3,4,5‐trisphosphate (PI(3,4,5)P_3_) to phosphatidylinositol‐4,5‐bisphosphate (PI(4,5)P_2_) (Solari et al., 2005). This leads to the inactivation of AKT kinases and subsequent activation of longevity‐promoting DAF‐16/FOXO (Reviewed in Altintas, Park, & Lee, 2016; Kenyon, 2010).

The PDZ (PSD‐95/Dlg‐1/ZO‐1) domain‐containing proteins (hereafter referred to as PDZ proteins) act as scaffolds for protein–protein interactions and mediate various cellular signaling processes (Reviewed in Kim & Sheng, 2004). PDZ domains are composed of six β‐sheets and two α‐helices and comprise ~90 amino acids. PDZ proteins typically bind the PDZ‐binding motifs that are located at the C‐terminal regions of the partner proteins. Roles of various PDZ proteins in cellular processes, such as signal transduction in neurons, are relatively well established; however, their function in aging and lifespan regulation is underexplored.

KIN‐4 is the sole homolog of the human microtubule‐associated serine/threonine kinase 1/2/3 (MAST1/2/3) in *C. elegans*, which contains a PDZ domain and a protein kinase domain (Manning, 2005). Human MAST family kinases are implicated in the inhibition of neurite outgrowth and regeneration in cultured cells (Loh, Francescut, Lingor, Bahr, & Nicotera, 2008). The sole *Drosophila* homolog of the human MAST kinase, Drop out, regulates dynein‐dependent transport during embryonic development (Hain et al., 2014). MAST kinases also bind to various proteins, including microtubules (Walden & Cowan, 1993), β2‐syntrophin (Lumeng et al., 1999), TNF receptor‐associated factor 6 (TRAF6) (Xiong, Li, Chen, Zhao, & Unkeless, 2004), cAMP‐regulated phospho‐protein (ARPP‐16) (Andrade et al., 2017), and PTEN (Valiente et al., 2005). Despite these findings, the role of MAST kinases in organismal aging remains unknown.

In the current study, we investigated the role of KIN‐4 in lifespan regulation conferred by reduced IIS in *C. elegans*. We used RNA interference (RNAi) to screen for genes encoding PDZ proteins that affected the lifespan of *C. elegans*. Our results showed that KIN‐4 was required for the long lifespan of *daf‐2* mutants. *kin‐4* was partly required for dauer formation and oxidative stress resistance in *daf‐2* mutants. Moreover, *kin‐4* in neurons was crucial for the extension of lifespan by *daf‐2* mutations. Through a large‐scale yeast two‐hybrid screen and subsequent protein–protein binding assays, we found that DAF‐18/PTEN bound to the PDZ domain of KIN‐4 through its C‐terminus. More importantly, the interaction between KIN‐4 and DAF‐18/PTEN was required to extend the lifespan of *daf‐2* mutants. Our findings suggest that MAST family kinases exert physiological effects on lifespan regulation via modulating IIS pathways through direct interaction with PTEN.

## 2 RESULTS

### 2.1 KIN‐4 is required for longevity conferred by reduced IIS

**Figure 1:**
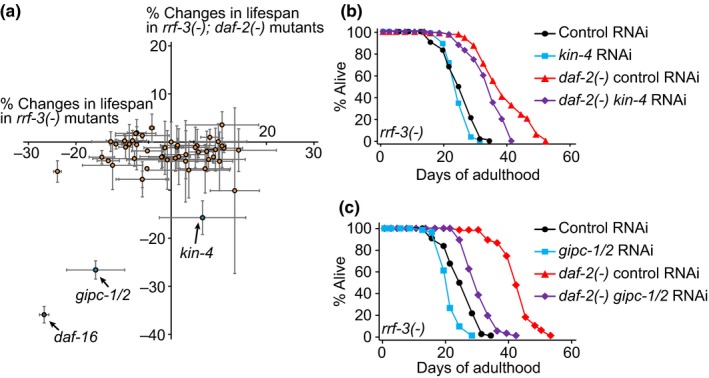
KIN‐4 is a PDZ protein that is required for the longevity of *daf‐2*/insulin/IGF‐1 receptor mutants. (a) A plot of percent changes in mean lifespan of *rrf‐3(pk1426) *[*rrf‐3(−)*] and *rrf‐3(pk1426); daf‐2(e1370)* [*rrf‐3(−); daf‐2(−)*] mutants upon knocking down each candidate PDZ protein‐encoding gene. Orange circles indicate the lifespan results after knocking down each targeted gene. Blue circles indicate the effects of *kin‐4* RNAi and *gipc‐1/‐2* RNAi on lifespan. A gray circle indicates the lifespan decrease by *daf‐16* RNAi that was used as a positive control. Error bars represent standard error of mean (*SEM*) of two independent lifespan experiments for each RNAi clone. (b, c) Knock‐down of* kin‐4* (b) or *gipc‐1/‐2 *(c) had a larger lifespan‐decreasing effect on *rrf‐3(−); daf‐2(−) *mutants than on *rrf‐3(−) *animals. See Supporting information Table S2 for statistics and additional repeats

**Figure 2:**
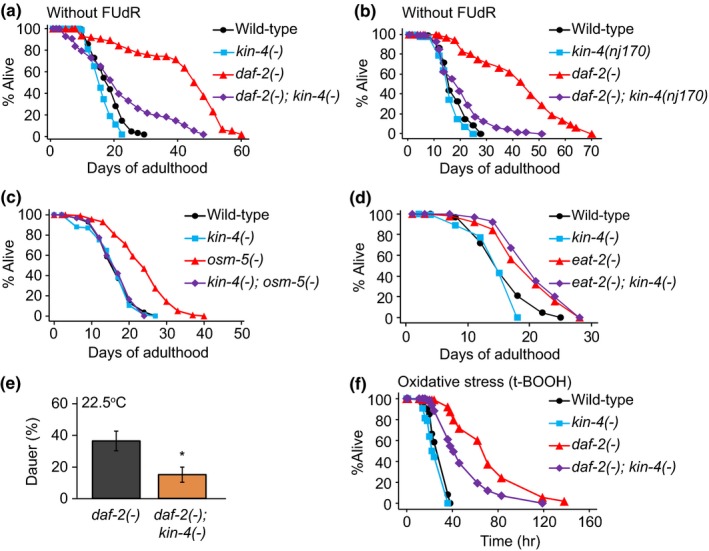
*kin‐4* is required for various phenotypes conferred by reduced insulin/IGF‐1 signaling. (a, b) *kin‐4(tm1049) *[*kin‐4(−)*] (a) and *kin‐4(nj170)* (b) mutation decreased the long lifespan of *daf‐2(e1370) *[*daf‐2(−)*] mutants without FUdR treatment. This lifespan‐shortening effect of *kin‐4(−)* mutation was confirmed by using *daf‐2(RNAi)* worms with or without FUdR treatment (Supporting information Figure S1d, e). (c) *kin‐4(−) *fully suppressed the lifespan extension by *osm‐5(p813)* [*osm‐5(−)*]. (d) *kin‐4(−)* mutation did not affect the lifespan increase by *eat‐2(ad1116)* [*eat‐2(−)*]. See Supporting information Table S3 for experimental repeats and statistics. (e) *kin‐4(−) *mutation reduced the dauer formation of *daf‐2(−) *mutants at 22.5°C (10 independent experiments with ≥33 worms for each trial. Error bars represent *SEM*. two‐tailed Student's *t* test. ∗ *p* < 0.05). See Supporting information Table S4 for statistical analysis. (f) The enhanced oxidative stress resistance of *daf‐2(−) *mutants was partially suppressed by *kin‐4(−) *mutation upon treating with 7.5 mM of tert‐Butyl hydroperoxide (t‐BOOH). See Supporting information Table S5 for statistics and experimental repeats

We aimed at identifying PDZ proteins that contribute to *C. elegans* longevity. We first identified 80 PDZ protein‐encoding genes by using Pfam and WormBase and by performing literature searches (Supporting information Table S1). We then examined the effect of each of the commercially available RNAi clones, targeting 49 candidate genes, on the lifespan of control worms and *daf‐2*/insulin/IGF‐1 receptor mutants (Figure 1a and Supporting information Table S2); we used *daf‐2* mutants that display a prominent longevity phenotype (Kenyon, 2010), as the effect of RNAi on the lifespan of these mutants would be more pronounced than on the wild‐type (Seo et al., 2015). Many PDZ proteins play roles in neurons (Reviewed in E. Kim & Sheng, 2004); because *C. elegans* neurons are refractory to RNAi (Timmons, Court, & Fire, 2001), we used *rrf‐3* mutant animals that display enhanced sensitivity to RNAi (Asikainen, Vartiainen, Lakso, Nass, & Wong, 2005) (Figure 1a; Supporting information Table S2). We found that RNAi targeting *kin‐4*, which encodes a MAST family kinase (Manning, 2005), or *gipc‐1* and *gipc‐2*, which encode GAIP‐interacting protein C proteins (Vartiainen, Pehkonen, Lakso, Nass, & Wong, 2006), had greater lifespan‐shortening effects on *daf‐2* mutants than on control animals (Figure 1a–c). We then determined the effects of genetic inhibition of *kin‐4* or *gipc‐1*/*‐2* on *daf‐2(–)* longevity using available loss of function mutants. Two independent *kin‐4* deletion mutations substantially suppressed the long lifespan of *daf‐2* mutants (Figure 2a, b; Supporting information Figure S1a, b), whereas *gipc‐1; gipc‐2* double mutations did not (Supporting information Figure S1c). These data suggest that *kin‐4* contributes to longevity caused by *daf‐2* mutations.

Next, we determined the effect of *kin‐4* mutation on longevity conferred by other gene mutations. The *kin‐4* mutation fully suppressed the longevity of the sensory neuron‐defective *osm‐5* mutants (Figure 2c), in which IIS is decreased (Apfeld & Kenyon, 1999), but did not affect the longevity of dietary restriction‐mimetic *eat‐2* mutants (Figure 2d). These data are consistent with the possibility that KIN‐4 functions in IIS for *C. elegans* longevity.

### 2.2 *kin‐4* is partly required for enhanced dauer formation and oxidative stress resistance caused by *daf‐2 *mutations

We sought to determine the role of KIN‐4 in other *daf‐2(−)*‐mediated physiological processes, including development and stress resistance. *kin‐4* mutations significantly reduced the formation of *daf‐2* mutation‐induced dauer, a hibernation‐like alternative larva (Hu, 2007), at 22.5°C but not at 25°C (Figure 2e; Supporting information Figure S2a). Additionally, the *kin‐4 *mutation partially suppressed the oxidative stress resistance of *daf‐2* mutants (Figure 2f) but further increased the enhanced pathogen resistance conferred by *daf‐2* mutations (Figure S2b). The thermotolerance of wild‐type and *daf‐2* mutant worms was not significantly affected by the *kin‐4* mutation (Supporting information Figure S2c). Thus, KIN‐4 appears to play roles in various IIS‐mediated physiological processes in a context‐dependent manner.

### 2.3 Neuronal *kin‐4* contributes to longevity conferred by *daf‐2* mutation

**Figure 3:**
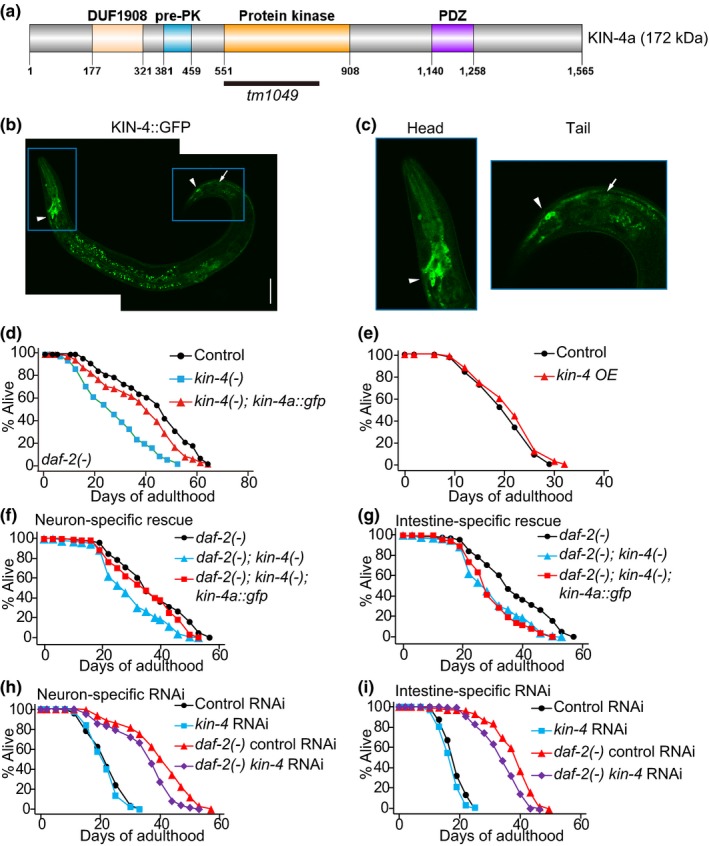
Neurons are crucial tissues for lifespan regulation by KIN‐4. (a) KIN‐4a is predicted to have 4 domains; DUF1908 whose function is not known, pre‐PK that is mainly found in MAST family kinases, protein kinase, and PDZ domains. The deleted part by *kin‐4(tm1049) *mutation is marked with a black solid line. (b, c) *kin‐4::gfp* transgene‐encoded protein (KIN‐4::GFP) was mainly expressed in head and tail (c) neurons (arrowheads) and dimly in the intestine (arrow). Scale bar indicates 50 μm. (d) KIN‐4a::GFP increased the shortened lifespan of *daf‐2(e1370); kin‐4(tm1049)* [*daf‐2(−); kin‐4(−)*] mutants. (e) *kin‐4* transgenic worms did not display longevity. We also found that *daf‐2* RNAi did not alter KIN‐4::GFP levels (Supporting information Figure S4d). See Supporting information Table S3 for experimental repeats and statistics. (f) Tissue‐specific promoter‐driven *kin‐4a* expression [*daf‐2(−); kin‐4(−); kin‐4a::gfp*] in neurons (*daf‐2(e1370); kin‐4(tm1049); rgef‐1p::kin‐4a::gfp*) prolonged the shortened lifespan of *daf‐2(e1370); kin‐4(tm1049) *[*daf‐2(−); kin‐4(−)*] mutants. (g) Transgenic expression of *kin‐4a* in the intestine (*daf‐2(−); kin‐4(−); ges‐1p::kin‐4a::gfp*) did not affect the decreased lifespan of *daf‐2(−); kin‐4(−) *mutants. (h) The long lifespan of *daf‐2(−) *mutants was decreased by neuron‐specific (*sid‐1(pk3321); uIs69[myo‐2p::mCherry; unc‐119p::sid‐1]*) *kin‐4* RNAi treatment. (i) Knock‐down of *kin‐4* in the intestine (*rde‐1(ne213); kbIs7[nhx‐2p::rde‐1; rol‐6(su1006)]*) significantly suppressed the long lifespan of *daf‐2* mutants. See Supporting information Table S3 for experimental repeats and statistics

KIN‐4 contains a protein kinase domain and a PDZ domain (Figure 3a) and is highly conserved in multiple species (Supporting information Figure S3a, b). To determine the expression pattern of KIN‐4 and the effect of *kin‐4* overexpression, we characterized transgenic animals expressing KIN‐4 tagged with the green fluorescent protein (GFP). We found that fusions of both the genomic *kin‐4* and *kin‐4* isoform a (*kin‐4a*) with *GFP* (*kin‐4::gfp* and *kin‐4a::gfp*, respectively) were expressed mainly in neurons and also in intestinal cells (Figure 3b, c; Supporting information Figure S4a). These results were confirmed using transgenic animals expressing *kin‐4* promoter‐driven GFP (*kin‐4p::gfp*) (Supporting information Figure S4b). The *kin‐4a::gfp *transgene largely restored longevity in *daf‐2; kin‐4* double mutants (Figure 3d) but did not affect the lifespan of wild‐type animals (Figure 3e; Supporting information Figure S4c).

To further test the tissue‐specific roles of KIN‐4 in longevity, we performed lifespan assays with both tissue‐specific *kin‐4* transgene and RNAi techniques. Neuronal expression of *kin‐4* (Figure S5a) rescued the shortened lifespan of *daf‐2; kin‐4* double mutants (Figure 3f). In contrast, *kin‐4* expression in the intestine or hypodermis (Figure S5b, c) had no effect on the lifespan of *daf‐2; kin‐4* mutants (Figure 3g; Supporting information Figure S5d). We then performed converse tissue‐specific *kin‐4* RNAi knock‐down experiments. We first confirmed that treatment with *kin‐4 *RNAi decreased *kin‐4* mRNA levels (Supporting information Figure S5e). Neuron‐specific knock‐down of *kin‐4* shortened the lifespan of *daf‐2* mutants but had minimal effects on the lifespan of control animals (Figure 3h). In addition, *kin‐4* knock‐down in the intestine significantly suppressed the longevity of *daf‐2* mutants (Figure 3i); however, *kin‐4* RNAi in other tissues had no effect on longevity (Supporting information Figure S5f–j). Altogether, these data indicate that KIN‐4 expression in neurons is necessary for long lifespan and sufficient to restore the longevity in *daf‐2 *mutants.

### 2.4 KIN‐4 does not seem to affect canonical DAF‐16/FOXO transcription factor activity in *daf‐2* mutants

DAF‐16/FOXO is an essential transcription factor that mediates the longevity of *daf‐2* mutants (Reviewed in Altintas et al., 2016; Kenyon, 2010). We therefore asked whether *kin‐4* influenced the activity of DAF‐16/FOXO for increasing lifespan in *daf‐2* mutants. We found that *kin‐4* mutations did not affect the nuclear localization of DAF‐16/FOXO in *daf‐2* mutants (Supporting information Figure S6a, b). Similarly, *kin‐4* mutations in *daf‐2* mutants did not reduce the mRNA levels of three selected DAF‐16 targets, *sod‐3*, *dod‐11*, and *mtl‐1* (Supporting information Figure S6c–e). We confirmed these results using *sod‐3p::gfp *transgenic animals (Supporting information Figure S6f). These data suggest that *kin‐4* influences reduced IIS‐mediated longevity by acting through factors other than DAF‐16/FOXO.

### 2.5 Identification of KIN‐4‐interacting proteins that contribute to the longevity of *daf‐2* mutants

**Figure 4:**
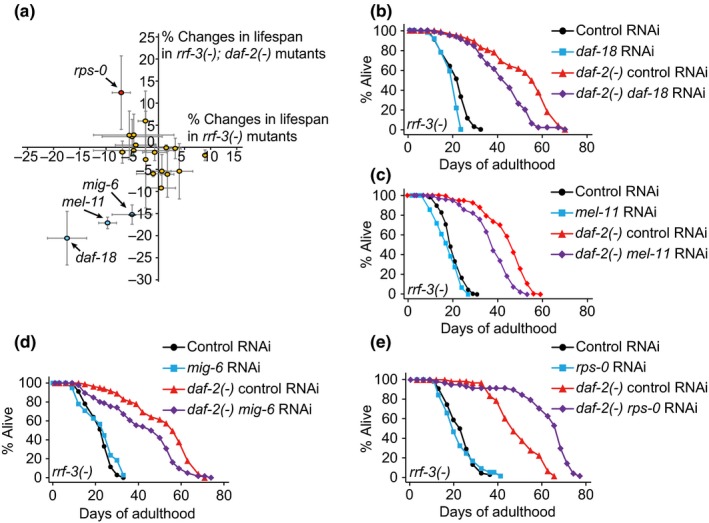
DAF‐18/PTEN binds KIN‐4 to affect the longevity of *daf‐2* mutants. (a) Percent lifespan changes by knocking down each of 21 genes that encode proteins that bound the PDZ domain of KIN‐4 in *rrf‐3(pk1426)* [*rrf‐3(−)*] and *rrf‐3(pk1426); daf‐2(e1370)* [*rrf‐3(−); daf‐2(−)*] mutants. Orange circles indicate the effects of RNAi clones on lifespan. Blue circles indicate RNAi clones that displayed bigger lifespan‐decreasing effects on *rrf‐3(−); daf‐2(−)* mutants than on *rrf‐3(−)* worms. A red circle indicates an RNAi clone that further increased the long lifespan of *rrf‐3(−); daf‐2(−)* mutants. Error bars represent *SEM* of mean survival times from two or three independent RNAi lifespan experiments. (b*−*d) *daf‐18* (b), *mel‐11* (c), and *mig‐6* (d) RNAi clones had bigger lifespan‐shortening effects on *rrf‐3(−); daf‐2(−) *mutant than on *rrf‐3(−)* animals. (e) *rps‐0* RNAi treatment further increased the long lifespan induced by *daf‐2(−)* mutation in an *rrf‐3(−)* background. See Supporting information Table S7 for experimental repeats and statistical analyses

Because KIN‐4 has a PDZ domain, we sought to identify proteins that bound to the PDZ domain, as these may contribute to longevity together with KIN‐4. We conducted an exhaustive yeast two‐hybrid screen using the PDZ domain of KIN‐4 as bait against *C. elegans *cDNA library and identified 41 prey proteins (Supporting information Figure S7). Of these, 23 proteins contained putative PDZ‐binding motifs at their C‐termini (Supporting information Table S6). To determine whether any of these 23 proteins were required for the longevity of *daf‐2* mutants, we performed an RNAi lifespan screen targeting 21 genes (Figure 4a; Supporting information Table S7). Knock‐down of *daf‐18/PTEN*, *mel‐11/myosin‐associated phosphatase regulatory subunit*, or *mig‐6/papilin and lacunin* had greater lifespan‐shortening effects on *daf‐2 *mutants than on control animals (Figure 4b–d). In addition, RNAi targeting *rps‐0*, which encodes a small ribosomal subunit SA protein, further extended the lifespan of *daf‐2 *mutants (Figure 4e). Among these candidates, the expression pattern of MEL‐11 (Wissmann, Ingles, & Mains, 1999) or MIG‐6 (Jafari et al., 2010; Kawano et al., 2009) is not predicted to overlap with that of KIN‐4, and the effect of *rps‐0* RNAi on lifespan was opposite of that caused by the genetic inhibition of *kin‐4*. Taking these results into consideration, we further analyzed the functional role of DAF‐18/PTEN in the longevity regulation by KIN‐4.

### 2.6 DAF‐18/PTEN binds to KIN‐4

**Figure 5:**
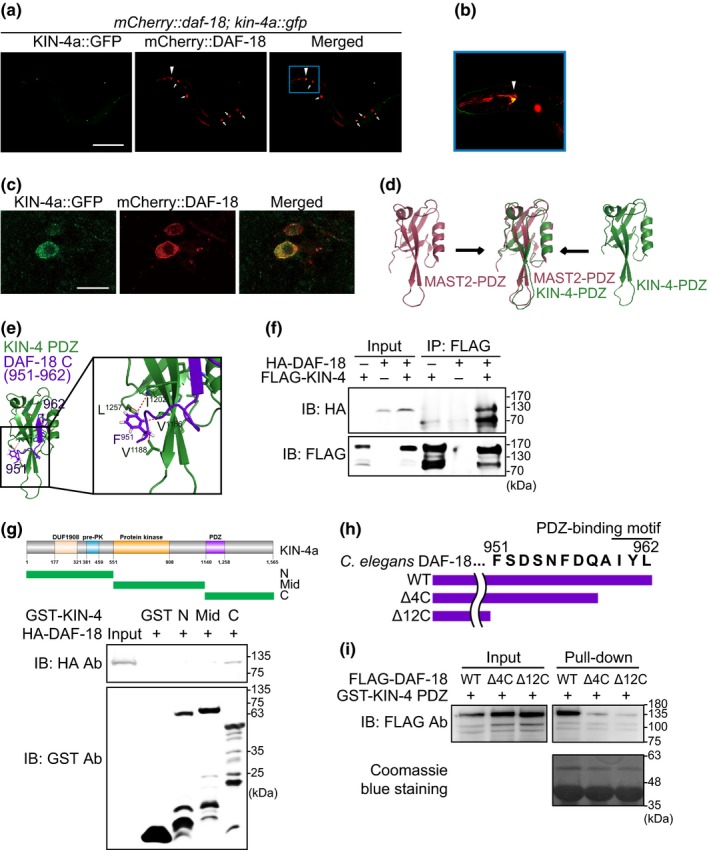
KIN‐4 physically interacts with DAF‐18/PTEN. (a‐c) Co‐localization of KIN‐4a::GFP and mCherry::DAF‐18. (a) DAF‐18 was mainly expressed in several head neurons (arrowhead). Arrows indicate red fluorescent signals of injection marker. Scale bar is 200 μm. (b) Shown in a blue rectangle is an enlarged picture of the head part in panel A. (c) Deconvolution images of a neuron. Subcellular localization of KIN‐4a::GFP and mCherry::DAF‐18 in a head neuron of *mCherry::daf‐18; kin‐4a::gfp; ofm‐1p::rfp* animals. Scale bar is 10 μm. (d) The PDZ domain of KIN‐4 (green) overlaps with that of MAST2 (magenta), one of human MAST family kinases. (e) The marked resides (V1186, V1188, I1202, and L1257) are conserved in the predicted structure of the PDZ domain of KIN‐4 (KIN‐4 PDZ, green) with that of human MAST2. F951 is a conserved residue in the C‐terminal region of DAF‐18 (DAF‐18 C, purple, See Supporting information Figure S8). DAF‐18 C from the 951st to the 962nd residues was predicted by substituting each residue of PTEN (2KYL) with a Coot program. (f) HA‐DAF‐18 was co‐immunoprecipitated by immunoprecipitating FLAG‐KIN‐4. IP: immunoprecipitation. IB: immunoblotting (g) A GST pull‐down assay of HA tag‐fused DAF‐18 [HA‐DAF‐18] with GST‐fused KIN‐4 fragment proteins. HA‐DAF‐18 was pulled down by using a PDZ domain‐containing KIN‐4 C‐terminal fragment [C], but not by an *N*‐terminal KIN‐4 fragment [N] or by a kinase domain‐containing KIN‐4 middle fragment [Mid]. See the upper illustration that depicts the domain regions in KIN‐4. (h) Illustration of wild‐type and deletion mutant DAF‐18 that were used for GST pull‐down assays. Wild‐type DAF‐18 [WT] has the intact DAF‐18 C‐terminal PDZ‐binding motif. DAF‐18 deletion mutant proteins, [Δ4C] and [Δ12C], do not contain the last 4 and 12 amino acids from its C‐terminal ends, respectively. (i) GST pull‐down assays using wild‐type or mutant FLAG‐tagged full‐length DAF‐18 [FLAG‐DAF‐18] with GST‐fused KIN‐4 PDZ domain [GST‐KIN‐4 PDZ] proteins. GST KIN‐4 PDZ strongly bound to FLAG‐DAF‐18 WT [WT] but weakly to FLAG‐DAF‐18 Δ4C [Δ4C] and FLAG‐DAF‐18 Δ12C [Δ12C]

DAF‐18/PTEN contributes to the longevity of *daf‐2* mutants by acting as a downstream phosphoinositide phosphatase (Reviewed in Altintas et al., 2016; Kenyon, 2010). Human PTEN interacts with MAST2, a homolog of KIN‐4, in cultured cells (Valiente et al., 2005), although the physiological role of this interaction has not been investigated. We determined the subcellular localization of KIN‐4 and DAF‐18 in vivo and found that KIN‐4::GFP and mCherry‐tagged DAF‐18 (mCherry::DAF‐18) co‐localized to the cytoplasm of neurons (Figures 5a–c). Next, we performed in silico analysis of the PDZ domain of KIN‐4 (Figure 5d), based on the crystal structure of the PDZ domain of human MAST2 (Terrien et al., 2012). Our structural modeling indicated potential interaction between the PDZ domain of KIN‐4 and the C‐terminal region of DAF‐18/PTEN (Figure 5e). Specifically, the C‐terminal PDZ‐binding motif of DAF‐18/PTEN was located in the groove of the PDZ domain of KIN‐4 (Figure 5e). Additionally, key residues in the human MAST2 that interact with human PTEN, including isoleucine (I1121), valine (V1123), methionine (M1136), and leucine (L1191) (Terrien et al., 2012), were highly conserved as V1186, V1188, I1202, and L1257, respectively, in KIN‐4 (Figure 5e). Moreover, amino acid residues including tyrosine (T401) and valine (V403) in the PDZ‐binding motif and phenylalanine (F392) at the C‐terminal region of human PTEN, which are important for the interaction with human MAST2 (Terrien et al., 2012), were generally conserved in *C. elegans* DAF‐18/PTEN as I960, L962, and F951, respectively (Figure 5e; Supporting information Figure S8).

We then experimentally tested the importance of the C‐terminal region of DAF‐18/PTEN for the interaction between KIN‐4 and DAF‐18/PTEN. Co‐immunoprecipitation assays using HEK293T cells confirmed that KIN‐4 bound to DAF‐18/PTEN (Figure 5f). Using glutathione S‐transferse (GST) pull‐down assays, we showed that the PDZ domain‐containing C‐terminal (C) region of KIN‐4 interacted with DAF‐18/PTEN, whereas the *N*‐terminal (N) or middle (Mid) region of KIN‐4 did not (Figure 5g). We then asked which regions and the residues of DAF‐18/PTEN were important for its interaction with KIN‐4 by generating FLAG‐tagged wild‐type and mutant DAF‐18/PTEN proteins and performing GST pull‐down assays using a GST‐fused KIN‐4 PDZ domain (Figure 5h–i). We found that 4‐amino acid and 12‐amino acid deletions at the C‐terminus (∆4C and ∆12C, respectively) substantially decreased the interaction between the PDZ domain of KIN‐4 and DAF‐18/PTEN (Figure 5i). These data indicate that the PDZ domain of KIN‐4 binds to the C‐terminal end of DAF‐18/PTEN.

### 2.7 Interaction between KIN‐4 and DAF‐18/PTEN is crucial for the longevity of *daf‐2* mutants

**Figure 6:**
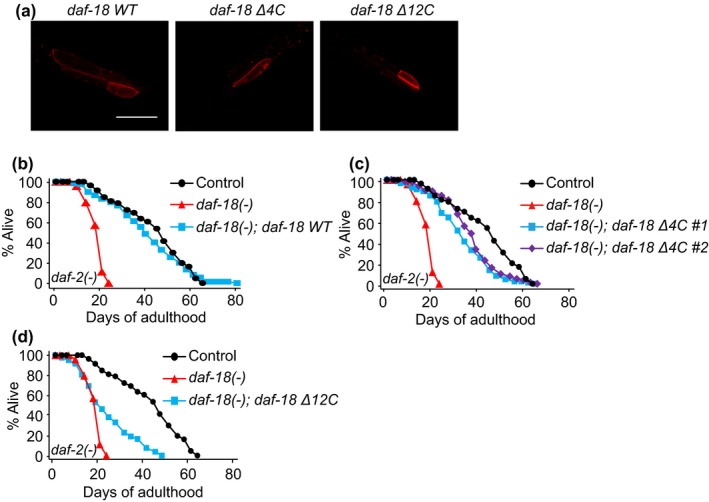
The interaction between KIN‐4 and DAF‐18 is crucial for longevity caused by IIS reduction. (a) Expression pattern of wild‐type [*daf‐18 WT*], the last four amino acid deletion mutant DAF‐18 transgene [*daf‐18 Δ4C*] and the last 12 amino acid deletion mutant *daf‐18* transgene [*daf‐18 Δ12C*], fused with mCherry in the posterior intestine cells. (b) *daf‐18 WT* fully suppressed lifespan reduction by *daf‐18(nr2037) *[*daf‐18(−)*] mutation in *daf‐2(e1370)* [*daf‐2(−)*] animals. (c) Two independent lines of *daf‐18 Δ4C* only partially increased the shortened lifespan of *daf‐2(−); daf‐18(−)* mutants. (d) *daf‐18 Δ12C* had small lifespan‐increasing effects on the shortened lifespan of *daf‐2(−); daf‐18(−)* animals. See Supporting information Table S3 for statistics and additional repeats

Next, we examined whether the interaction between KIN‐4 and DAF‐18/PTEN contributed to reduced IIS‐mediated longevity. We generated transgenic animals expressing mutant forms of DAF‐18/PTEN proteins carrying ∆4C and ∆12C fused with mCherry (*daf‐18 Δ4C* and *daf‐18 Δ12C*, respectively) and those expressing mCherry‐tagged wild‐type (WT) DAF‐18 (*daf‐18 WT*) as a control. Expression of all these three mCherry‐fused DAF‐18/PTEN proteins was detected, and their subcellular localization was enriched at the plasma membrane (Figure 6a). We found that *daf‐18 WT* fully restored the longevity of *daf‐2; daf‐18* double mutants (Figure 6b). In contrast, *daf‐18 Δ4C* or *daf‐18 Δ12C* only partially restored longevity in *daf‐2; daf‐18* double mutants (Figure 6c, d). These data suggest that the C‐terminal end of DAF‐18/PTEN, which mediates the physical interaction with KIN‐4, is required for the full longevity of *daf‐2* mutants. We also found that *kin‐4* mutation further shortened the lifespan of *daf‐18 Δ12C‐*expressing *daf‐2; daf‐18* mutants (Supporting information Figure S9). These data indicate that KIN‐4 promotes longevity in *daf‐2* mutants via other factors in addition to DAF‐18, and/or that DAF‐18 C‐terminal region promotes *daf-2(-)* longevity via KIN‐4‐independent targets. Altogether, our data suggest that KIN‐4 contributes to longevity in *daf‐2* mutants partly through binding to DAF‐18/PTEN at the C‐terminus.

## 3 DISCUSSION

### 3.1 *C. elegans* MAST family kinase KIN‐4 promotes longevity via binding DAF‐18/PTEN

PDZ proteins function as scaffolds that organize proteins for transducing various intracellular signals. However, the role of PDZ proteins in IIS, an important evolutionarily conserved lifespan‐regulatory signaling, has been poorly understood. In this study, we showed that KIN‐4/MAST kinase was a lifespan‐regulatory PDZ protein acting through DAF‐18/PTEN. KIN‐4, mainly in neurons, influenced the longevity conferred by reduced IIS. We also identified DAF‐18/PTEN as a key interacting protein of KIN‐4. The interaction between KIN‐4 and DAF‐18/PTEN was important for the lifespan regulation by IIS. Our study suggests that KIN‐4/MAST family kinase is a novel longevity‐promoting protein that acts in IIS via binding DAF‐18/PTEN protein.

### 3.2 KIN‐4 affects lifespan differently from previously reported PDZ proteins, MICS‐1 and MPZ‐1

Previous studies have reported lifespan‐influencing PDZ proteins, including MICS‐1/synaptojanin 2 binding protein (SYNJ2BP) and multi‐PDZ domain scaffold protein (MPZ‐1), in *C. elegans* (Hoffmann et al., 2012; Palmitessa & Benovic, 2010). The genetic inhibition of *mics‐1* promotes longevity (Hoffmann et al., 2012), and *mpz‐1* knock‐down increases lifespan, perhaps by activating DAF‐18/PTEN (Palmitessa & Benovic, 2010). Both *mics‐1* and *mpz‐1* genes were included in our RNAi lifespan screen (Supporting information Table S2); however, RNAi targeting of *mics‐1* or *mpz‐1* had small or no effect on lifespan in wild‐type or *daf‐2* mutant animals (Figure 1a; Supporting information Table S2). In contrast, *kin‐4 *mutation or RNAi substantially suppressed the extended lifespan of *daf‐2* mutants. This difference may have arisen from different genetic backgrounds and/or experimental conditions; for example, *mics‐1* RNAi lifespan assay was performed at 25°C (Hoffmann et al., 2012), and *mpz‐1* RNAi was applied for two generations to wild‐type worms (Palmitessa & Benovic, 2010). Here, we performed lifespan assays using RNAi‐sensitive *rrf‐3* mutants treated with each of these RNAi clones only during adulthood at 20°C. Despite the difference, previous reports do not conflict with our data showing that KIN‐4 is required for the extended lifespan of *C. elegans* conferred by reduced IIS. We speculate that KIN‐4 contributes to IIS‐mediated longevity in a manner distinct from MPZ‐1 or MICS‐1.

### 3.3 *kin‐4* mutation appears to elicit a compensatory activation of DAF‐16/FOXO in *daf‐2* mutants

Although a major protein acting downstream of DAF‐18/PTEN lipid phosphatase is DAF‐16/FOXO, we did not find evidence supporting the possibility that KIN‐4 acts with DAF‐18/PTEN for increasing DAF‐16/FOXO activity. In contrast, DAF‐16/FOXO activity in *daf‐2* mutant animals appears to be further increased by *kin‐4(−) *mutations (Supporting information Figure S6). We speculate that *kin‐4(−)* mutations decrease lifespan in *daf‐2* mutants via DAF‐18/PTEN while acting through factors other than DAF‐16/FOXO and that this in turn enhances DAF‐16/FOXO activity as a compensatory response. Genetic inhibition of several DAF‐16/FOXO‐independent factors that contribute to reduced IIS‐mediated longevity has been shown to elicit compensatory activation of DAF‐16/FOXO. For example, we previously reported that mutations in *smg‐2/UPF1*, a key factor for nonsense‐mediated mRNA decay, decrease the longevity of *daf‐2* mutants, while further increasing DAF‐16/FOXO activity (Son et al., 2017). In addition, PQM‐1, paraquat (methyl viologen)‐responsive transcription factor, is required for longevity in *daf‐2* mutants, but genetic inhibition of *pqm‐1* increases the nuclear localization of DAF‐16 (Tepper et al., 2013). It will be interesting to determine whether KIN‐4 and DAF‐18/PTEN act with DAF‐16/FOXO‐independent longevity factors for contributing to long lifespan in *C. elegans* with reduced IIS.

### 3.4 KIN‐4 may increase DAF‐18/PTEN activity via phosphorylation

Post‐translational modifications of PTEN, including phosphorylation, are important for its regulation (Fragoso & Barata, 2015). In many cases, phosphorylation at the C‐terminal region of PTEN leads to its conformational change that inhibits the lipid phosphatase activity of PTEN (Fragoso & Barata, 2015). On the other hand, Rak tyrosine kinase and polo‐like kinase 3 (Plk3) activate PTEN by phosphorylating the C2 domain and C‐terminal part of PTEN, respectively (Xu, Yao, Jiang, Lu, & Dai, 2010; Yim et al., 2009). In addition, RhoA‐associated kinase (ROCK) phosphorylates PTEN and increases its activity (Li et al., 2005). Mammalian MAST2 also phosphorylates PTEN in vitro (Valiente et al., 2005), although its effect on the activity of PTEN remains unexplored. Given the similar lifespan phenotypes caused by mutations in *daf‐18*/PTEN and *kin‐4*, we speculate that *C. elegans* KIN‐4 may increase the activity of DAF‐18/PTEN through binding and phosphorylation.

### 3.5 KIN‐4 contributes to longevity possibly through altering the protein phosphatase activity of DAF‐18/PTEN

Taking into consideration that DAF‐18/PTEN has dual phosphatase activity for both proteins and lipids (Fragoso & Barata, 2015), it seems likely that the binding of KIN‐4 may affect the phosphatase activity of DAF‐18/PTEN for as yet unidentified substrate proteins. In mammals, substrate proteins of PTEN phosphatase include protein kinase B/Akt and cyclic AMP response element‐binding protein (CREB; Gu et al., 2011; Phadngam et al., 2016). Therefore, homologs of these proteins may mediate the effects of the interaction between KIN‐4 and DAF‐18/PTEN on the longevity of *C. elegans*. One limitation regarding this scenario is that tyrosine 138, which is essential for the protein phosphatase activity of PTEN in mammals (Davidson et al., 2010), is replaced by leucine in *C. elegans* DAF‐18/PTEN. Nevertheless, *C. elegans* DAF‐18/PTEN can act as a protein phosphatase for VAB‐1/ephrin receptor (Brisbin et al., 2009). Therefore, it will be important to identify and functionally characterize protein substrates of DAF‐18/PTEN in *C. elegans* in future research, with respect to the role of KIN‐4 in protein–protein interaction and longevity.

### 3.6 KIN‐4 appears to contribute to longevity in *daf‐2* mutants by acting with other factors as well as with DAF‐18/PTEN

In this paper, we showed that physical interaction between KIN‐4 and DAF‐18/PTEN is required for full longevity of *daf‐2* mutants. However, KIN‐4 also seems to contribute to reduced IIS‐mediated longevity through additional factors. The mammalian MAST kinases bind various proteins, including microtubules (Walden & Cowan, 1993), β2‐syntrophin (Lumeng et al., 1999), TRAF6 (Xiong et al., 2004), and ARPP‐16 (Andrade et al., 2017). We also identified various factors that bound the PDZ domain of *C. elegans* KIN‐4 through a yeast two‐hybrid screen (Supporting information Table S6). Thus, KIN‐4 binding to some of these proteins may contribute to longevity in *daf‐2* mutants. In addition, MAST2 and MAST3 kinases phosphorylate Na^+^/H^+^ exchanger 3 (NHE3; Wang et al., 2006) and ARPP‐16 (Andrade et al., 2017), respectively, and therefore, phosphorylation of *C. elegans* homologs of these proteins by KIN‐4 may affect reduced IIS‐mediated longevity. It will be important to test these possibilities by performing biochemical and molecular genetic experiments in future studies.

### 3.7 Interaction between MAST kinases and PTEN may play important roles in mammalian physiology

Several lines of evidence indicate that MAST kinases are crucial for the physiology of cancer cells (Eissmann et al., 2013; Robinson et al., 2011; Tomoshige et al., 2015). In some breast cancer cells, MAST1 and MAST2 are fused with other proteins through recurrent gene rearrangements; these fusion proteins act as putative tumorigenic drivers (Robinson et al., 2011). A pedigree study reported that *MAST1* variants are associated with lung cancer (Tomoshige et al., 2015). Furthermore, MAST2 influences glioblastoma tumor growth, potentially as an apoptosis suppressor (Eissmann et al., 2013). In this study, we characterized the physiological role of the physical interaction between KIN‐4 and DAF‐18/PTEN in *C. elegans*. Considering evolutionarily conserved amino acid sequences of these proteins, our study may provide insights into the role of MAST kinases and PTEN in tumorigenesis and aging, thus contributing toward the development of therapeutic strategies for anti‐cancer and anti‐aging medicine.

## 4 EXPERIMENTAL PROCEDURES

### 4.1 Strains

All strains used in this study are described in the Supporting Information.

### 4.2 Identification of PDZ proteins

Identification of potential *C. elegans* PDZ proteins was performed by using combination of bioinformatic methods similarly to a previous paper (Seo et al., 2015). See the supporting information for details.

### 4.3 Lifespan assays

Lifespan assays were conducted at 20°C as previously described, with some modifications (Lee et al., 2015). See the Supporting Information for details.

### 4.4 Dauer formation

Dauer assays were performed as previously described, with some modifications (Gaglia et al., 2012). Gravid adult worms were placed on OP50‐seeded NGM plates and were allowed to lay eggs for 3 hr at 22.5°C and 25°C. The number of dauers and total worms was counted when non‐dauer worms reached L4 or young adult stage. Error bars indicate *SEM*, and statistics were calculated by using Student's *t* test.

### 4.5 Stress resistance assays

Stress resistance assays were performed by following a previous report with some modifications (Seo et al., 2015). See the Supporting Information for details.

### 4.6 Generation of transgenic worms

Transgenic worms were generated as previously described, with some modification (Artan et al., 2016). See the Supporting Information for details.

### 4.7 Generation of mutant strain using CRISPR

To generate *kin‐4(nj170)*, the homologous recombination was performed essentially by following a previous report (Dickinson, Ward, Reiner, & Goldstein, 2013). First, the *kin‐4* locus was replaced by a repair template (pSN588) that carries the *loxP‐unc‐119(+)‐loxP* sequence flanked by the left and right arms corresponding to the 1st intron and the 3′ UTR genomic sequence of *kin‐4*, respectively. This repair template was injected into *unc‐119(ed3) *animals with *peft‐3::Cas9* and an sgRNA plasmid (pSN598) to generate a double‐stranded break at the *kin‐4* locus. Progeny were screened for animals in which the *kin‐4* locus was replaced by the repair template. The *loxP‐unc‐119(+)‐loxP* cassette was subsequently removed by injecting *peft‐3::Cre *plasmid (pDD104). The animals in which the *unc‐119(+) *gene was excised were isolated and were confirmed by DNA sequence analysis. The resulting strain carries a 17 kb deletion in the *kin‐4* locus and removes the sequence between 24,629 of the cosmid F22B3 and 11,644 of the cosmid C10C6. The *unc‐119(ed3)* mutation was then crossed out to obtain IK2019.

### 4.8 Yeast two‐hybrid screen

Yeast two‐hybrid screen was performed by Panbionet (http://panbionet.com) following a previous report (Kim et al., 2008). See the Supporting information for details.

### 4.9 Prediction of protein domains, structure modeling and sequence alignment, and generation of the phylogenic tree

Prediction of protein domains, structure modeling and sequence alignment, and generation of the phylogenic tree were performed similarly to a previous paper (Hwang et al., 2015), with modifications. See the Supporting Information for details.

### 4.10 Co‐immunoprecipitation and immunoblotting

Co‐immunoprecipitation and immunoblotting were performed following a previous report (Seo et al., 2015) with some modifications. See the Supporting Information for details.

### 4.11 GST pull‐down assay

GST pull‐down assays were performed following methods described in a previous report (Dev, Nishimune, Henley, & Nakanishi, 1999) with some modifications. See the Supporting Information for details.

### 4.12 Microscopy

Microscopy experiments were executed as previously described with some modifications (Son et al., 2017). See the Supporting Information for details.

### 4.13 Quantitative RT–PCR analysis

Quantitative RT–PCR experiments were performed as previously described with some modifications (Son et al., 2017). See the Supporting information for details.

## CONFLICT OF INTEREST

None declared.

## AUTHOR CONTRIBUTIONS

S.W.A.A. contributed to designing and performing the majority of experimental assays in this manuscript, data analysis, and writing manuscript; E.‐S.C. initiated the project and performed survival assay experiments and data analysis; W.H., H.G.S., K.S., and E.J.E.K. contributed to survival assay experiments; J.‐S.Y., H.‐J.N., and S.K. contributed to bioinformatics analysis; N.T.H.N. and J.‐Y.Y. contributed to performing biochemistry experiments; B.K.S., S.K.P., Y.R., and C.M.H. contributed to confocal microscopy imaging; Y.K. contributed to prediction of protein structure modeling; S.N. and I.M. contributed to generating crucial mutants and transgenic worms for this manuscript; S.‐J.V.L. contributed to designing all experiments, performing the initial RNAi screen, some survival assay experiments, data analysis, and writing manuscript.
