# Supplementary material for: KIN‐4/MAST kinase promotes PTEN‐mediated longevity of Caenorhabditis elegans via binding through a PDZ domain
Source: Aging Cell. 2019 Feb 17;18(3):e12906. doi: 10.1111/acel.12906 (PMC6516182; doi:10.1111/acel.12906)
Supplement: Supplementary file 1 [file ACEL-18-e12906-s001.docx]

**Supplementary Figure Legends**

**FIGURE S1** Genetic inhibition of *kin-4* shortens the extended lifespan of *daf-2* mutants. (a, b) *kin-4(tm1049)* [*kin-4(−)*] (a) and *kin-4(nj170)* (b) mutations substantially suppressed the longevity of *daf-2(−)* mutants. (c) *gipc-1(hc192); gipc-2(ok1172)* [*gipc-1(−); gipc-2(−)*] double mutation slightly decreased the long lifespan of *daf-2(e1370)* [*daf-2(−)*] mutants. (d, e) *kin-4* mutation had a bigger lifespan-shortening effects on *daf-2* RNAi-treated worms than on control RNAi-treated animals with (d) or without FUdR (e). See Table S3 for statistics and additional experiments.

**FIGURE S2** The effects of *kin-4* mutation on pathogen resistance and thermotolerance in *daf-2* mutants. (a) *kin-4(tm1049)* [*kin-4(−)*] mutation did not affect the dauer formation of *daf-2(−)* mutants at 25^o^C (three independent experiments with ≥56 worms for each trial. Error bars represent two-tailed Student’s *t−*test. ∗ p<0.05). See Table S4 for statistical analysis. (b, c) Resistance of *daf-2(e1370)* [*daf-2(−)*] mutants against *P. aeruginosa* (PA14) was enhanced by *kin-4(−)* mutation (b), whereas heat stress (35°C) resistance was not influenced (c). See Table S5 for experimental repeats and statistical analyses.

**FIGURE S3** KIN-4 is highly conserved among species. (a) A phylogenic tree showing *C. elegans* KIN-4 and its homologs in other species. Scale bar indicates the number of substitutions per amino acid residue. (b) Domain illustration of MAST2 and KIN-4a and schematic illustration of KIN-4 fragments.

**FIGURE S4** *kin-4* is expressed in neurons and the intestine. (a) Expression pattern of KIN-4a::GFP (*kin-4p::kin-4a::gfp; odr-1p::rfp*). Arrowheads (neurons) and an arrow (the intestine) indicate KIN-4a::GFP signals. Scale bar is 200 μm. Images were obtained at young adult stage. (b) An image of *kin-4* promoter-driven reporter GFP (*kin-4p::gfp; ofm-1p::rfp*). Arrowheads (neurons) and arrows (intestinal cells) indicate GFP expression driven by a *kin-4* promoter. Scale bar is 100 μm. Images were obtained at L2/L3 stages. (c) Two independent lines of *kin-4a*-overexpressing transgenic worms did not display longevity. (d) Knocking down *daf-2* did not influence the expression of KIN-4::GFP in head or tail region (n≥28 from total duplicate experimental sets).

**FIGURE S5** Validation of *kin-4* transgene expression and *kin-4* RNAi. (a-c) Expression pattern of neuron-specific *kin-4a* (*rgef-1p::kin-4a::gfp; odr-1p::rfp*) (a), intestine-specific *kin-4a* (*ges-1p::kin-4a::gfp; odr-1p::rfp*) (b), and hypodermis-specific *kin-4a* (*lin-26p::kin-4a::gfp; odr-1p::rfp*) (c) transgenes in *daf-2(e1370); kin-4(tm1049)* animals. The microphotographs were obtained by using L2/L3 stage worms. Scale bar is 100 μm. Arrowheads indicate GFP signals. (d) Transgenic expression of *kin-4a* in the hypodermis (*daf-2(−); kin-4(−); lin-26p::kin-4a::gfp*) did not affect the decreased lifespan of *daf-2(−); kin-4(−)* mutants. (e) Treatment with *kin-4* RNAi reduced the mRNA levels of *kin-4* in wild-type as well as *daf-2(e1370)* [*daf-2(−)*] animals (n=3). *kin-4* mRNA levels were measured by using qRT-PCR. Error bars indicate SEM (two-tailed Student’s *t−*test, **p<*0.05, ****p<*0.001). (f*−*h) *kin-4* RNAi in the hypodermis (*rde-1(ne219); kzIs9[pKK1260(lin-26p::nls::GFP); pKK1253(lin-26p::rde-1); pRF6(rol-6(su1006)]*) (f), seam cells and the hypodermis (*rde-1(ne219); Is[wrt-2p::RDE-1]*) (g), or muscles (*rde-1(ne219); kzIs20[pDM#715(hlh-1p::rde-1); pTG95(sur-5p::nls::GFP)]*) (h) did not affect the lifespan phenotype of *daf-2(−)* mutants. (i) RNAi knock-down of *kin-4* did not affect the lifespan of wild-type or *daf-2(−)* animals in systemic-RNAi defective *sid-1(pk3321)* [*sid-1(−)*] backgrounds. (j) *kin-4* RNAi did not influence lifespan in wild-type or *daf-2(−)* animals in RNAi-defective *rde-1(ne219)* [*rde-1(−)*] mutant backgrounds. See Table S3 for experimental repeats and statistics.

**FIGURE S6** *kin-4* mutation does not decrease DAF-16/FOXO activity in *daf-2* mutants. (a, b) Subcellular localization of DAF-16::GFP was not changed by *kin-4(tm1049)* [*kin-4(−)*] mutation in *daf-2(e1370)* [*daf-2(−)*] mutants. Representative images of DAF-16::GFP (a) and the quantification (b) (n≥30 from total triplicate experimental sets, Chi-Square test). Scale bar is 100 μm. (c-e) mRNA levels of *sod-3* (c), *dod-11* (d), and *mtl-1* (e), measured by qRT-PCR, were further increased by *kin-4(−)* mutation in *daf-2(−)* mutants (n=3). (f) *kin-4(−)* mutation also further increased the elevated *sod-3p::gfp* expression in *daf-2(−)* mutants (n≥17). Error bars represent SEM (two-tailed Student’s *t−*test, **p<*0.05, ***p<*0.01, ****p<*0.001).

**FIGURE S7** A yeast two-hybrid screen to identify proteins that bind KIN-4. The PDZ domain of KIN-4 was used as bait, and 41 preys were identified. The interactions were confirmed through yeast growth on two different selective deficient (SD) media lacking leucine, tryptophan, and alanine [SD-LWA] and selective deficient (SD) media lacking leucine, tryptophan, and uracil [SD-LWU]. pPC86 was used as a negative control and polypyrimidine-tract-binding protein (PTB)/PTB as a positive control. See Table S6 for the list of identified KIN-4-binding proteins from the screen.

**FIGURE S8** Evolutionarily conserved PDZ-binding motifs in DAF-18. The comparison of C-terminal sequences between *C. elegans* DAF-18 and PTEN in other species. DAF-18 has F951 and a PDZ-binding motif at the C-terminal region.

**FIGURE S9** *kin-4* mutation further decreased the lifespan of *daf-2(−); daf-18(−); daf-18 Δ12C* animals. The lifespan-decreasing effect of *kin-4(tm1049)* [*kin-4(−)*] was statistically significant for two out of three trials. See Table S3 for experimental repeats and statistics.

**Supplementary Tables**

**TABLE S1** List of PDZ domain-containing protein-encoding genes

| **Gene/transcript name** | **Description** | **Included in RNAi lifespan screen** |
| --- | --- | --- |
| *magu-2*/ C01B7.4 | a protein with similarity to the MAGUK (membrane-associated guanylate kinase) family of proteins | O |
| C01B7.5 | a PDZ domain-containing protein conserved amongst nematodes | O |
| *nrfl-1*/ C01F6.6 | an ortholog of Drosophila SIP1 that maintains epithelial integrity via regulation of Moesin activity, and of the human SLC9A3R1, SLC9A3R2, PDZK1, and PDZD3 proteins | O |
| *glr-1*/ C06E1.4 | an AMPA-type ionotropic glutamate receptor | - |
| C09G1.4 | an ortholog of human PARD6G (PAR-6 family cell polarity regulator gamma), PARD6A (PAR-6 family cell polarity regulator alpha) and PARD6B (PAR-6 family cell polarity regulator beta) | O |
| *lin-10*/ C09H6.2 | a PDZ and PTB domain-containing protein that is homologous to mammalian Munc interacting proteins | - |
| *kin-4*/ C10C6.1 | an ortholog of members of the human PDZ (PDZ domain containing) family including MAST1 | O |
| *tiam-1*/ C11D9.1 | an ortholog of members of the human ARHGEF (Rho guanine nucleotide exchange factors) family including TIAM2 | O |
| *dlg-1*/ C25F6.2 | a MAGUK protein, orthologous to Drosophila Discs large | - |
| *smz-1*/ C25G4.6 | a PDZ domain-containing protein | O |
| *dsh-2*/ C27A2.6 | one of three *C. elegans* Dishevelled (Dsh) homologs | O |
| *shn-1*/ C33B4.3 | the sole *elegans* SHANK protein and orthlog of vertebrate SHANK3 (SH3 and multiple ankyrin repeat domains 3) | O |
| *dsh-1*/ C34F11.9 | a homolog of Drosophila DISHEVELED and a paralog of MIG-5 (and DSH-2) | O |
| *gipc-1*/ C35D10.2 | an ortholog of human GIPC3 (GIPC PDZ domain containing family member 3), GIPC1 (GIPC PDZ domain containing family member 1) and GIPC2 (GIPC PDZ domain containing family member 2) | O |
| *gpr-2*/ C38C10.4 | a protein containing a GPR (G Protein Regulator)/GoLoco motif characteristic of guanine nucleotide exchange factors specific for G-alpha GTPases | - |
| *nab-1*/ C43E11.6 | a PDZ and SAM domain-containing protein that is the sole *C. elegans* ortholog of the mammalian Neurabin/Spinophilin proteins | O |
| *psmd-9*/ C44B7.1 | an ortholog of human PSMD9 (proteasome 26S subunit, non-ATPase 9) | O |
| C45G9.7 | an ortholog of human TAX1BP3 (Tax1-binding protein 3) | O |
| *ptp-1*/ C48D5.2 | a non-receptor tyrosine phosphatase containing Band 4.1 and PDZ domains | O |
| C50D2.3 | uncharacterized | - |
| C50F7.3 | uncharacterized | - |
| C50F7.6 | an ortholog of human PDZD4 (PDZ domain containing 4), PDZRN3 (PDZ domain containing ring finger 3) and PDZRN4 (PDZ domain containing ring finger 4) | - |
| C52A11.3 | a PDZ domain-containing protein | O |
| *mpz-1*/ C52A11.4 | a multi-PDZ domain scaffold protein | O |
| C53B4.4 | an ortholog of human PDZD8 (PDZ domain containing 8) | - |
| *pkc-3*/ F09E5.1 | an atypical protein kinase that lacks a DAG binding domain | - |
| *rhgf-1*/ F13E6.6 | an RGS RhoGEF (Regulator of G-protein Signaling Rho Guanine Nucleotide Exchange Factor) | - |
| *lin-2*/ F17E5.1 | a protein belonging to the membrane associated guanylate kinase (MAGUK) family, with several domains (L27, PDZ, SH3, and guanylate kinase) thought to assemble specific multiprotein complexes in particular regions of the cell | - |
| *mpz-3*/ F18C5.4 | a multiple PDZ domain protein | O |
| F20D6.1 | uncharacterized | - |
| F23B2.8 | uncharacterized | O |
| F23C8.13 | an ortholog of human *dlg1* (discs large MAGUK scaffold protein 1) and DLG4 (discs large MAGUK scaffold protein 4) | - |
| *snx-27*/ F25H2.2 | an ortholog of human SNX27 (sorting nexin family member 27) | O |
| *let-413*/ F26D11.11 | a protein with strong similarity to human ERBIN, rat DENSIN, Drosophila Scribble and its human ortholog hSCRIB | O |
| *stn-2*/ F27D9.8 | a gamma-syntrophin | O |
| F28E10.4 | uncharacterized | - |
| *lim-8*/ F28F5.3 | a protein containing one PDZ and one LIM domain | - |
| *stn-1*/ F30A10.8 | a syntrophin, a PDZ and PH domain-containing adaptor protein, with homology to vertebrate alpha and beta-syntrophins | - |
| *gras-1*/ F30F8.3 | an ortholog of human GRASP (general receptor for phosphoinositides 1 associated scaffold protein) and CYTIP (cytohesin 1 interacting protein) | - |
| *syd-1*/ F35D2.5 | a Rho-GTPase-like activating domain as well as PDZ and C2 domains | O |
| F40F9.3 | uncharacterized | O |
| *elks-1*/ F42A6.9 | the *C. elegans* homolog of the vertebrate ELKS (glutamine, leucine, lysine, and serine-rich) proteins | - |
| *magu-4*/ F44D12.1 | an ortholog of human DLG5 (discs large MAGUK scaffold protein 5) | O |
| *gipc-2*/ F44D12.4 | an ortholog of human GIPC3 (GIPC PDZ domain containing family member 3), GIPC1 (GIPC PDZ domain containing family member 1) and GIPC2 (GIPC PDZ domain containing family member 2) | O |
| *cla-1*/ F45E4.3 | a protein containing C-terminal PDZ and C2 Ca^2+^-binding domains that is related to the mammalian active zone protein Piccolo/Aczonin | O |
| *par-3*/ F54E7.3 | a PDZ domain-containing protein orthologous to mammalian atypical PKC isotype-specific interacting protein (ASIP) and Drosophila Bazooka | - |
| *rgs-7*/ F56B6.2 | three isoforms of an atypical regulator of G protein signaling (RGS) protein that contains both a C2 and an RGS domain, and that binds EGL-30 via both domains, similar in organization to mammalian PDZ-RGS3 | - |
| *ser-1*/ F59C12.2 | a putative ortholog of mammalian 5-HT2 metabotropic serotonin receptors | - |
| *mpz-4*/ H06H21.9 | a *Caenorhabditis*-specific protein with two PDZ domains | O |
| *frm-8*/ H09G03.2 | a protein containing a WW domain, a PDZ domain, and a FERM domain with homology to the human predicted protein KIAA0316 | O |
| *magi-1*/ K01A6.2 | a multi PDZ-domain containing tight junction-associated protein and members of vertebrate membrane associated guanylate-kinase (MAGUK) family | O |
| *cnk-1*/ R01H10.8 | a protein that contains a SAM domain, a PDZ domain, and a PH domain | O |
| *tbc-10*/ R06B10.5 | an ortholog of human TBC1D10C (TBC1 domain family member 10C), TBC1D10A (TBC1 domain family member 10A) and TBC1D10B (TBC1 domain family member 10B) | - |
| *mpz-5*/ R11A8.8 | uncharacterized | - |
| *mig-5*/ T05C12.6 | one of three *C. elegans* Dishevelled homologs | O |
| *unc-10*/ T10A3.1 | a protein with zinc-finger, Q/N-rich, PDZ, and C2 domains that is homologous to vertebrate Rim1 | O |
| *alp-1*/ T11B7.4 | the *C. elegans* ortholog of the ALP (alpha-actininassociated LIM protein)-Enigma family of proteins | O |
| *pxf-1*/ T14G10.2 | an ortholog of human RAPGEF2 (Rap guanine nucleotide exchange factor 2), RAPGEF6 (Rap guanine nucleotide exchange factor 6) and CTC-432M15.3 | O |
| T15H9.4 | uncharacterized | O |
| T19B10.5 | a protein with partial similarity to human PERIAXIN, which when mutated leads to Dejerine-Sottas neuropathy | O |
| *mics-1*/ T21C9.1 | an ortholog of human SYNJ2BP-COX16 (SYNJ2BP-COX16 readthrough) and SYNJ2BP (synaptojanin 2 binding protein) | O |
| *smz-2*/ T21G5.4 | a PDZ domain-containing protein | O |
| *par-6*/ T26E3.3 | a PDZ-domain-containing protein that is conserved in Drosophila and mammals | O |
| *afd-1*/ W03F11.6 | an ortholog of human MLLT4 | - |
| *zoo-1*/ Y105E8A.26 | a protein ortholog of the zonula occludens (ZO) subfamily of membrane-associated guanylate kinases (MAGUKs) | - |
| *frm-5.2*/ Y38C1AB.4 | an ortholog of human FRMPD2 (FERM and PDZ domain containing 2) and PTPN13 (protein tyrosine phosphatase, non-receptor type 13) | O |
| *frm-5.1*/ Y38C1AB.8 | a protein that contains a FERM (Band 4.1-ezrin-radixin-moesin) domain near the center of the molecule and two C-terminal PDZ domains | - |
| Y42H9AR.1 | an ortholog of human GORASP1 (Golgi reassembly stacking protein 1) and GORASP2 (Golgi reassembly stacking protein 2) | O |
| *mpz-6*/ Y51B9A.3 | uncharacterized | O |
| Y52E8A.1 | uncharacterized | - |
| *lin-7*/ Y54G11A.10 | a protein that contains a PDZ domain and an L27 domain | O |
| *magu-1*/ Y55B1BR.4 | an ortholog of human MPP7 (membrane palmitoylated protein 7), MPP3 (membrane palmitoylated protein 3) and MPP4 (membrane palmitoylated protein 4) | O |
| Y57G11C.22 | an ortholog of human PICK1 (protein interacting with PRKCA 1) | O |
| *npr-29*/ ZC84.4 | a G protein-coupled receptor with acidic amino acids in the -3 position of its PDZ binding motif | - |
| ZK1321.4 | an ortholog of human LDB3 (LIM domain binding 3), PDLIM7 (PDZ and LIM domain 7) and PDLIM5 (PDZ and LIM domain 5) | - |
| ZK849.1 | an ortholog of human PDZD7 (PDZ domain containing 7) and DFNB31 | O |
| *gopc-1*/ ZK849.2 | an ortholog of human GOPC (golgi associated PDZ and coiled-coil motif containing) | O |
| C46H11.6 | involved in hermaphrodite genitalia development and locomotion | - |
| *magu-3*/ C50F2.8 | an ortholog of human MPP6 (membrane palmitoylated protein 6) and MPP2 (membrane palmitoylated protein 2) | O |
| *sipa-1*/ T27F2.2 | a homolog of human SIPA1/EHBP1L1 | - |

**TABLE S2** Analysis of RNAi lifespan assay results targeting each of PDZ domain-containing protein-encoding genes

| **Strain** | **Mean lifespan ±s.e.m. (days)** | **75th percentile** | **% change** | **Number of animals that died/total** | ***p* value vs. control** | **Figure in text** |
| --- | --- | --- | --- | --- | --- | --- |
| *rrf-3(pk1426)* control RNAi | 24.96±0.6 | 28 |  | 61/75 |  | Figure 1b and c |
| *rrf-3(pk1426) daf-16* RNAi | 17.03±0.6 | 21 | -32% | 36/50 | <0.0001 |  |
| *rrf-3(pk1426) magu-2* RNAi | 26.75±0.6 | 31 | 7% | 58/75 | 0.0647 |  |
| *rrf-3(pk1426)* C01B7.5 RNAi | 25.94±0.6 | 31 | 4% | 67/75 | 0.3567 |  |
| *rrf-3(pk1426) nrfl-1* RNAi | 25.71±0.8 | 31 | 3% | 28/75 | 0.4472 |  |
| *rrf-3(pk1426) kin-4* RNAi | 24.34±0.4 | 28 | -2% | 61/75 | 0.0721 | Figure 1b |
| *rrf-3(pk1426) tiam-1* RNAi | 25.51±1.0 | 28 | 2% | 19/67 | 0.7492 |  |
| *rrf-3(pk1426) gipc-1/2* RNAi | 21.13±0.4 | 24 | -15% | 70/75 | <0.0001 | Figure 1c |
| *rrf-3(pk1426); daf-2(e1370)* control RNAi | 38.45±1.1 | 44 |  | 51/75 |  | Figure 1b |
| *rrf-3(pk1426); daf-2(e1370) daf-16* RNAi | 24.83±0.9 | 29 | -35%*^rrf-3; daf-2^* | 49/75 | <0.0001 *^rrf-3;^ ^daf-2^* |  |
| *rrf-3(pk1426); daf-2(e1370) magu-2* RNAi | 37.61±1.0 | 41 | -2% *^rrf-3; daf-2^* | 38/75 | 0.5311 *^rrf-3;^ ^daf-2^* |  |
| *rrf-3(pk1426); daf-2(e1370)* C01B7.5 RNAi | 37.05±0.9 | 44 | -4% *^rrf-3; daf-2^* | 49/75 | 0.1271 *^rrf-3;^ ^daf-2^* |  |
| *rrf-3(pk1426); daf-2(e1370) nrfl-1* RNAi | 38.76±1.1 | 46 | 1% *^rrf-3; daf-2^* | 50/75 | 0.9659 *^rrf-3;^ ^daf-2^* |  |
| *rrf-3(pk1426); daf-2(e1370) kin-4* RNAi | 33.74±0.9 | 38 | -12% *^rrf-3; daf-2^* | 31/75 | 0.0037 *^rrf-3;^ ^daf-2^* | Figure 1b |
| *rrf-3(pk1426); daf-2(e1370) tiam-1* RNAi | 36.38±0.9 | 41 | -5% *^rrf-3; daf-2^* | 62/75 | 0.0636 *^rrf-3;^ ^daf-2^* |  |
| *rrf-3(pk1426)* control RNAi | 24.36±0.8 | 29 |  | 61/75 |  |  |
| *rrf-3(pk1426) daf-16* RNAi | 19.1±0.6 | 22 | -22% | 61/75 | <0.0001 |  |
| *rrf-3(pk1426) magu-2* RNAi | 28.02±0.8 | 32 | 15% | 61/77 | 0.0003 |  |
| *rrf-3(pk1426)* C01B7.5 RNAi | 28.23±0.7 | 32 | 16% | 74/75 | 0.0001 |  |
| *rrf-3(pk1426) nrfl-1* RNAi | 28.84±0.9 | 34 | 18% | 64/76 | <0.0001 |  |
| *rrf-3(pk1426) kin-4* RNAi | 28.18±0.8 | 34 | 16% | 61/75 | 0.0002 |  |
| *rrf-3(pk1426) tiam-1* RNAi | 26.3±0.9 | 32 | 8% | 53/76 | 0.0484 |  |
| *rrf-3(pk1426) gipc-1/2* RNAi | 22.68±0.8 | 27 | -7% | 62/76 | 0.1041 |  |
| *rrf-3(pk1426); daf-2(e1370)* control RNAi | 43.66±1.1 | 49 |  | 68/75 |  |  |
| *rrf-3(pk1426); daf-2(e1370) daf-16* RNAi | 27.76±1.0 | 34 | -36% *^rrf-3; daf-2^* | 67/76 | <0.0001 *^rrf-3;^ ^daf-2^* |  |
| *rrf-3(pk1426); daf-2(e1370) magu-2* RNAi | 43.49±1.2 | 52 | 0% *^rrf-3; daf-2^* | 68/75 | 0.6646 *^rrf-3;^ ^daf-2^* |  |
| *rrf-3(pk1426); daf-2(e1370)* C01B7.5 RNAi | 45.32±1.2 | 52 | 4% *^rrf-3; daf-2^* | 66/75 | 0.2055 *^rrf-3;^ ^daf-2^* |  |
| *rrf-3(pk1426); daf-2(e1370) nrfl-1* RNAi | 46.41±1.2 | 52 | 6% *^rrf-3; daf-2^* | 58/75 | 0.0273 *^rrf-3;^ ^daf-2^* |  |
| *rrf-3(pk1426); daf-2(e1370) kin-4* RNAi | 35.26±0.9 | 40 | -19% *^rrf-3; daf-2^* | 69/75 | <0.0001 *^rrf-3;^ ^daf-2^* |  |
| *rrf-3(pk1426); daf-2(e1370) tiam-1* RNAi | 45.01±1.0 | 49 | 3% *^rrf-3; daf-2^* | 69/75 | 0.4116 *^rrf-3;^ ^daf-2^* |  |
| *rrf-3(pk1426)* control RNAi | 27.08±1.3 | 33 |  | 20/50 |  |  |
| *rrf-3(pk1426) daf-16* RNAi | 15.9±0.6 | 21 | -41% | 46/75 | <0.0001 |  |
| *rrf-3(pk1426)* C09G1.4 RNAi | 22.03±0.6 | 25 | -19% | 50/75 | 0.0003 |  |
| *rrf-3(pk1426) smz-1* RNAi | 28.78±1.4 | 33 | 6% | 27/75 | 0.3815 |  |
| *rrf-3(pk1426) dsh-2* RNAi | 26.86±0.8 | 33 | -1% | 42/75 | 0.6157 |  |
| *rrf-3(pk1426) shn-1* RNAi | 25.26±1.0 | 29 | -7% | 40/75 | 0.3271 |  |
| *rrf-3(pk1426) dsh-1* RNAi | 25.08±1.0 | 31 | -7% | 37/75 | 0.2374 |  |
| *rrf-3(pk1426) nab-1* RNAi | 24.26±0.8 | 29 | -10% | 43/75 | 0.0529 |  |
| *rrf-3(pk1426) psmd-9* RNAi | 25.77±0.8 | 31 | -5% | 47/75 | 0.2747 |  |
| *rrf-3(pk1426)* C45G9.7 RNAi | 24.54±0.8 | 29 | -9% | 37/75 | 0.0592 |  |
| *rrf-3(pk1426) ptp-1* RNAi | 26.7±1.0 | 31 | -1% | 43/74 | 0.8696 |  |
| *rrf-3(pk1426)* C52A11.3 RNAi | 26.73±0.9 | 31 | -1% | 44/76 | 0.7544 |  |
| *rrf-3(pk1426); daf-2(e1370)* control RNAi | 42.61±0.8 | 45 |  | 41/73 |  | Figure 1c |
| *rrf-3(pk1426); daf-2(e1370) daf-16* RNAi | 24.19±0.6 | 27 | -43% *^rrf-3;^ ^daf-2^* | 48/75 | <0.0001 *^rrf-3;^ ^daf-2^* |  |
| *rrf-3(pk1426); daf-2(e1370)* C09G1.4 RNAi | 38.39±1.1 | 42 | -10% *^rrf-3;^ ^daf-2^* | 41/75 | 0.0158 *^rrf-3;^ ^daf-2^* |  |
| *rrf-3(pk1426); daf-2(e1370) smz-1* RNAi | 39.99±0.9 | 45 | -6% *^rrf-3;^ ^daf-2^* | 48/75 | 0.0546 *^rrf-3;^ ^daf-2^* |  |
| *rrf-3(pk1426); daf-2(e1370) dsh-2* RNAi | 38.84±0.9 | 42 | -9% *^rrf-3;^ ^daf-2^* | 44/73 | 0.0016 *^rrf-3;^ ^daf-2^* |  |
| *rrf-3(pk1426); daf-2(e1370) shn-1* RNAi | 40.31±1.0 | 45 | -5% *^rrf-3;^ ^daf-2^* | 38/75 | 0.1167 *^rrf-3;^ ^daf-2^* |  |
| *rrf-3(pk1426); daf-2(e1370) dsh-1* RNAi | 39.76±1.0 | 45 | -7% *^rrf-3;^ ^daf-2^* | 46/76 | 0.0691 *^rrf-3;^ ^daf-2^* |  |
| *rrf-3(pk1426); daf-2(e1370) gipc-1/2* RNAi | 30.46±0.6 | 33 | -29% *^rrf-3;^ ^daf-2^* | 47/75 | <0.0001 *^rrf-3;^ ^daf-2^* | Figure 1c |
| *rrf-3(pk1426); daf-2(e1370) nab-1* RNAi | 40.67±1.0 | 45 | -5% *^rrf-3;^ ^daf-2^* | 48/74 | 0.2724 *^rrf-3;^ ^daf-2^* |  |
| *rrf-3(pk1426); daf-2(e1370) psmd-9* RNAi | 37.65±0.9 | 42 | -12% *^rrf-3;^ ^daf-2^* | 40/75 | 0.0001 *^rrf-3;^ ^daf-2^* |  |
| *rrf-3(pk1426); daf-2(e1370)* C45G9.7 RNAi | 38.42±0.9 | 42 | -10% *^rrf-3;^ ^daf-2^* | 49/75 | 0.0005 *^rrf-3;^ ^daf-2^* |  |
| *rrf-3(pk1426); daf-2(e1370) ptp-1* RNAi | 39.36±1.0 | 45 | -8% *^rrf-3;^ ^daf-2^* | 42/74 | 0.021 *^rrf-3;^ ^daf-2^* |  |
| *rrf-3(pk1426)* control RNAi | 25.13±0.9 | 31 |  | 55/75 |  |  |
| *rrf-3(pk1426) daf-16* RNAi | 15.28±0.4 | 19 | -39% | 49/74 | <0.0001 |  |
| *rrf-3(pk1426)* C09G1.4 RNAi | 23.63±0.9 | 29 | -6% | 53/75 | 0.2145 |  |
| *rrf-3(pk1426) smz-1* RNAi | 30.68±0.9 | 35 | 22% | 56/75 | <0.0001 |  |
| *rrf-3(pk1426) dsh-2* RNAi | 26.14±0.8 | 31 | 4% | 63/76 | 0.485 |  |
| *rrf-3(pk1426) shn-1* RNAi | 25.81±0.8 | 31 | 3% | 59/75 | 0.5646 |  |
| *rrf-3(pk1426) dsh-1* RNAi | 26.06±0.9 | 31 | 4% | 56/75 | 0.4899 |  |
| *rrf-3(pk1426) nab-1* RNAi | 23.33±0.8 | 29 | -7% | 63/75 | 0.0835 |  |
| *rrf-3(pk1426) psmd-9* RNAi | 28.21±0.7 | 33 | 12% | 64/75 | 0.0152 |  |
| *rrf-3(pk1426)* C45G9.7 RNAi | 27.08±0.9 | 33 | 8% | 59/75 | 0.0858 |  |
| *rrf-3(pk1426) ptp-1* RNAi | 27.04±0.9 | 33 | 8% | 52/74 | 0.1257 |  |
| *rrf-3(pk1426)* C52A11.3 RNAi | 28.84±0.8 | 33 | 15% | 56/75 | 0.0037 |  |
| *rrf-3(pk1426); daf-2(e1370)* control RNAi | 41.34±0.8 | 45 |  | 60/73 |  |  |
| *rrf-3(pk1426); daf-2(e1370) daf-16* RNAi | 26.16±1.0 | 30 | -37% *^rrf-3;^ ^daf-2^* | 50/59 | <0.0001 *^rrf-3;^ ^daf-2^* |  |
| *rrf-3(pk1426); daf-2(e1370)* C09G1.4 RNAi | 41.42±1.0 | 45 | 0% *^rrf-3;^ ^daf-2^* | 59/70 | 0.8214 *^rrf-3;^ ^daf-2^* |  |
| *rrf-3(pk1426); daf-2(e1370) smz-1* RNAi | 42.47±1.0 | 48 | 3% *^rrf-3;^ ^daf-2^* | 64/75 | 0.1101 *^rrf-3;^ ^daf-2^* |  |
| *rrf-3(pk1426); daf-2(e1370) dsh-2* RNAi | 42.47±0.8 | 48 | 3% *^rrf-3;^ ^daf-2^* | 55/65 | 0.5607 *^rrf-3;^ ^daf-2^* |  |
| *rrf-3(pk1426); daf-2(e1370) shn-1* RNAi | 40.98±1.0 | 48 | -1% *^rrf-3;^ ^daf-2^* | 58/75 | 0.7407 *^rrf-3;^ ^daf-2^* |  |
| *rrf-3(pk1426); daf-2(e1370) dsh-1* RNAi | 41.59±0.9 | 45 | 1% *^rrf-3;^ ^daf-2^* | 52/75 | 0.7993 *^rrf-3;^ ^daf-2^* |  |
| *rrf-3(pk1426); daf-2(e1370) gipc-1/2* RNAi | 31.12±0.6 | 33 | -25% *^rrf-3;^ ^daf-2^* | 62/75 | <0.0001 *^rrf-3;^ ^daf-2^* |  |
| *rrf-3(pk1426); daf-2(e1370) nab-1* RNAi | 42.85±0.9 | 48 | 4% *^rrf-3;^ ^daf-2^* | 66/75 | 0.1552 *^rrf-3;^ ^daf-2^* |  |
| *rrf-3(pk1426); daf-2(e1370) psmd-9* RNAi | 41.31±0.7 | 45 | 0% *^rrf-3;^ ^daf-2^* | 62/75 | 0.4227 *^rrf-3;^ ^daf-2^* |  |
| *rrf-3(pk1426); daf-2(e1370)* C45G9.7 RNAi | 42.33±1.0 | 48 | 2% *^rrf-3;^ ^daf-2^* | 56/73 | 0.5011 *^rrf-3;^ ^daf-2^* |  |
| *rrf-3(pk1426); daf-2(e1370) ptp-1* RNAi | 42.32±0.9 | 48 | 2% *^rrf-3;^ ^daf-2^* | 59/75 | 0.3183 *^rrf-3;^ ^daf-2^* |  |
| *rrf-3(pk1426)* control RNAi | 26.78±1.1 | 32 |  | 37/50 |  |  |
| *rrf-3(pk1426) daf-16* RNAi | 19.3±0.6 | 24 | -28% | 46/75 | <0.0001 |  |
| *rrf-3(pk1426) mpz-1* RNAi | 27.71±0.8 | 32 | 3% | 44/75 | 0.5096 |  |
| *rrf-3(pk1426) mpz-3* RNAi | 30.11±1.0 | 34 | 12% | 46/76 | 0.0167 |  |
| *rrf-3(pk1426)* F23B2.8 RNAi | 27.46±0.8 | 32 | 3% | 49/75 | 0.9866 |  |
| *rrf-3(pk1426) snx-27* RNAi | 29.43±0.8 | 34 | 10% | 58/75 | 0.0692 |  |
| *rrf-3(pk1426) let-413* RNAi | 29.72±0.9 | 34 | 11% | 49/75 | 0.0378 |  |
| *rrf-3(pk1426) stn-2* RNAi | 29.1±1.0 | 34 | 9% | 35/75 | 0.1201 |  |
| *rrf-3(pk1426) syd-1* RNAi | 30.25±0.8 | 34 | 13% | 46/75 | 0.0213 |  |
| *rrf-3(pk1426)* F40F9.3 RNAi | 29.1±0.8 | 34 | 9% | 41/74 | 0.1875 |  |
| *rrf-3(pk1426) magu-4* RNAi | 30.95±1.1 | 36 | 16% | 35/75 | 0.0055 |  |
| *rrf-3(pk1426) gipc-2* RNAi | 28.96±1.1 | 32 | 8% | 40/74 | 0.2029 |  |
| *rrf-3(pk1426); daf-2(e1370)* control RNAi | 47.17±0.7 | 54 |  | 45/75 |  |  |
| *rrf-3(pk1426); daf-2(e1370) daf-16* RNAi | 27.33±0.9 | 30 | -42% *^rrf-3;^ ^daf-2^* | 48/75 | <0.0001 *^rrf-3;^ ^daf-2^* |  |
| *rrf-3(pk1426); daf-2(e1370)* C52A11.3 RNAi | 44.58±0.9 | 51 | -5% *^rrf-3;^ ^daf-2^* | 53/75 | 0.0182 *^rrf-3;^ ^daf-2^* |  |
| *rrf-3(pk1426); daf-2(e1370) mpz-1* RNAi | 44.64±0.9 | 51 | -5% *^rrf-3;^ ^daf-2^* | 39/75 | 0.0051 *^rrf-3;^ ^daf-2^* |  |
| *rrf-3(pk1426); daf-2(e1370) mpz-3* RNAi | 42.16±0.9 | 48 | -11% *^rrf-3;^ ^daf-2^* | 57/75 | <0.0001 *^rrf-3;^ ^daf-2^* |  |
| *rrf-3(pk1426); daf-2(e1370)* F23B2.8 RNAi | 44.3±0.9 | 48 | -6% *^rrf-3;^ ^daf-2^* | 28/50 | 0.0046 *^rrf-3;^ ^daf-2^* |  |
| *rrf-3(pk1426); daf-2(e1370) snx-27* RNAi | 44.66±0.9 | 51 | -5% *^rrf-3;^ ^daf-2^* | 42/75 | 0.0529 *^rrf-3;^ ^daf-2^* |  |
| *rrf-3(pk1426); daf-2(e1370) let-413* RNAi | 44.32±0.8 | 48 | -6% *^rrf-3;^ ^daf-2^* | 50/75 | 0.001 *^rrf-3;^ ^daf-2^* |  |
| *rrf-3(pk1426); daf-2(e1370) stn-2* RNAi | 44.25±0.9 | 48 | -6% *^rrf-3;^ ^daf-2^* | 48/75 | 0.0076 *^rrf-3;^ ^daf-2^* |  |
| *rrf-3(pk1426); daf-2(e1370) syd-1* RNAi | 42.39±0.9 | 48 | -10% *^rrf-3;^ ^daf-2^* | 46/75 | 0.0001 *^rrf-3;^ ^daf-2^* |  |
| *rrf-3(pk1426); daf-2(e1370)* F40F9.3 RNAi | 41.28±0.7 | 44 | -12% *^rrf-3;^ ^daf-2^* | 52/75 | <0.0001 *^rrf-3;^ ^daf-2^* |  |
| *rrf-3(pk1426);daf-2(e1370) magu-4* RNAi | 44.34±0.9 | 51 | -6% *^rrf-3;^ ^daf-2^* | 61/75 | 0.0033 *^rrf-3;^ ^daf-2^* |  |
| *rrf-3(pk1426)* control RNAi | 28.11±0.6 | 32 |  | 55/75 |  |  |
| *rrf-3(pk1426) daf-16* RNAi | 17.64±0.4 | 21 | -37% | 61/75 | <0.0001 |  |
| *rrf-3(pk1426) mpz-1* RNAi | 26.4±0.8 | 32 | -6% | 58/75 | 0.3415 |  |
| *rrf-3(pk1426) mpz-3* RNAi | 28.63±0.9 | 32 | 2% | 53/74 | 0.2747 |  |
| *rrf-3(pk1426)* F23B2.8 RNAi | 27.9±0.7 | 32 | -1% | 57/75 | 0.732 |  |
| *rrf-3(pk1426) snx-27* RNAi | 31.34±1.0 | 36 | 11% | 52/74 | 0.0001 |  |
| *rrf-3(pk1426) let-413* RNAi | 30.26±0.8 | 34 | 8% | 47/75 | 0.0112 |  |
| *rrf-3(pk1426) stn-2* RNAi | 29.88±0.9 | 34 | 6% | 45/75 | 0.0378 |  |
| *rrf-3(pk1426) syd-1* RNAi | 30.18±0.7 | 32 | 7% | 45/75 | 0.0424 |  |
| *rrf-3(pk1426)* F40F9.3 RNAi | 27.38±0.8 | 32 | -3% | 51/74 | 0.7174 |  |
| *rrf-3(pk1426) magu-4* RNAi | 28.15±0.7 | 32 | 0% | 53/75 | 0.8072 |  |
| *rrf-3(pk1426) gipc-2* RNAi | 29.34±0.9 | 36 | 4% | 39/50 | 0.1731 |  |
| *rrf-3(pk1426); daf-2(e1370)* control RNAi | 49.28±1.1 | 56 |  | 64/75 |  |  |
| *rrf-3(pk1426); daf-2(e1370) daf-16* RNAi | 25.43±0.7 | 29 | -48% *^rrf-3;^ ^daf-2^* | 62/75 | <0.0001 *^rrf-3;^ ^daf-2^* |  |
| *rrf-3(pk1426); daf-2(e1370)* C52A11.3 RNAi | 50.94±1.0 | 56 | 3% *^rrf-3;^ ^daf-2^* | 61/75 | 0.3585 *^rrf-3;^ ^daf-2^* |  |
| *rrf-3(pk1426); daf-2(e1370) mpz-1* RNAi | 50.73±1.1 | 56 | 3% *^rrf-3;^ ^daf-2^* | 61/75 | 0.1235 *^rrf-3;^ ^daf-2^* |  |
| *rrf-3(pk1426); daf-2(e1370) mpz-3* RNAi | 49.02±1.0 | 53 | -1% *^rrf-3;^ ^daf-2^* | 58/73 | 0.6674 *^rrf-3;^ ^daf-2^* |  |
| *rrf-3(pk1426); daf-2(e1370)* F23B2.8 RNAi | 48.97±1.2 | 56 | -1% *^rrf-3;^ ^daf-2^* | 64/75 | 0.5876 *^rrf-3;^ ^daf-2^* |  |
| *rrf-3(pk1426); daf-2(e1370) snx-27* RNAi | 51.08±1.0 | 56 | 4% *^rrf-3;^ ^daf-2^* | 64/74 | 0.1968 *^rrf-3;^ ^daf-2^* |  |
| *rrf-3(pk1426); daf-2(e1370) let-413* RNAi | 50.7±1.0 | 56 | 3% *^rrf-3;^ ^daf-2^* | 64/75 | 0.2883 *^rrf-3;^ ^daf-2^* |  |
| *rrf-3(pk1426); daf-2(e1370) stn-2* RNAi | 50.39±1.2 | 56 | 2% *^rrf-3;^ ^daf-2^* | 60/74 | 0.2415 *^rrf-3;^ ^daf-2^* |  |
| *rrf-3(pk1426); daf-2(e1370) syd-1* RNAi | 50.31±0.9 | 56 | 2% *^rrf-3;^ ^daf-2^* | 64/75 | 0.9625 *^rrf-3;^ ^daf-2^* |  |
| *rrf-3(pk1426); daf-2(e1370)* F40F9.3 RNAi | 51.48±1.0 | 56 | 4% *^rrf-3;^ ^daf-2^* | 58/75 | 0.165 *^rrf-3;^ ^daf-2^* |  |
| *rrf-3(pk1426);daf-2(e1370) magu-4* RNAi | 48.66±1.7 | 59 | -1% *^rrf-3;^ ^daf-2^* | 41/50 | 0.2868 *^rrf-3;^ ^daf-2^* |  |
| *rrf-3(pk1426)* control RNAi | 29.08±1.2 | 34 |  | 47/75 |  |  |
| *rrf-3(pk1426) daf-16* RNAi | 15.71±0.4 | 19 | -46% | 55/75 | <0.0001 |  |
| *rrf-3(pk1426) cla-1* RNAi | 26.71±1.4 | 32 | -8% | 42/75 | 0.2302 |  |
| *rrf-3(pk1426) mpz-4* RNAi | 25.11±1.0 | 28 | -14% | 50/66 | 0.0119 |  |
| *rrf-3(pk1426) frm-8* RNAi | 29.58±1.2 | 34 | 2% | 36/50 | 0.8179 |  |
| *rrf-3(pk1426) magi-1* RNAi | 25.62±1.5 | 32 | -12% | 40/68 | 0.0699 |  |
| *rrf-3(pk1426) cnk-1* RNAi | 26.56±0.9 | 30 | -9% | 43/75 | 0.0642 |  |
| *rrf-3(pk1426) mig-5* RNAi | 26.09±1.6 | 32 | -10% | 37/56 | 0.2476 |  |
| *rrf-3(pk1426) unc-10* RNAi | 27.55±1.3 | 36 | -5% | 56/75 | 0.5032 |  |
| *rrf-3(pk1426) alp-1* RNAi | 25.92±1.0 | 32 | -11% | 49/75 | 0.0358 |  |
| *rrf-3(pk1426) pxf-1* RNAi | 27.58±1.2 | 34 | -5% | 62/75 | 0.4664 |  |
| *rrf-3(pk1426)* T15H9.4 RNAi | 26.95±1.1 | 30 | -7% | 49/75 | 0.1341 |  |
| *rrf-3(pk1426); daf-2(e1370)* control RNAi | 44.29±0.8 | 49 |  | 43/72 |  |  |
| *rrf-3(pk1426); daf-2(e1370) daf-16* RNAi | 26.69±0.8 | 31 | -40% *^rrf-3;^ ^daf-2^* | 38/75 | <0.0001 *^rrf-3;^ ^daf-2^* |  |
| *rrf-3(pk1426); daf-2(e1370) gipc-2* RNAi | 44.61±0.8 | 49 | 1% *^rrf-3;^ ^daf-2^* | 49/75 | 0.7685 *^rrf-3;^ ^daf-2^* |  |
| *rrf-3(pk1426); daf-2(e1370) cla-1* RNAi | 42.78±0.7 | 46 | -3% *^rrf-3;^ ^daf-2^* | 58/76 | 0.0961 *^rrf-3;^ ^daf-2^* |  |
| *rrf-3(pk1426); daf-2(e1370) mpz-4* RNAi | 43.81±0.6 | 49 | -1% *^rrf-3;^ ^daf-2^* | 59/75 | 0.2896 *^rrf-3;^ ^daf-2^* |  |
| *rrf-3(pk1426); daf-2(e1370) frm-8* RNAi | 43.15±1.0 | 49 | -3% *^rrf-3;^ ^daf-2^* | 44/75 | 0.6971 *^rrf-3;^ ^daf-2^* |  |
| *rrf-3(pk1426); daf-2(e1370) magi-1* RNAi | 43.51±0.8 | 46 | -2% *^rrf-3;^ ^daf-2^* | 48/75 | 0.5576 *^rrf-3;^ ^daf-2^* |  |
| *rrf-3(pk1426); daf-2(e1370) cnk-1* RNAi | 45.23±1.2 | 52 | 2% *^rrf-3;^ ^daf-2^* | 47/75 | 0.0995 *^rrf-3;^ ^daf-2^* |  |
| *rrf-3(pk1426); daf-2(e1370) mig-5* RNAi | 43.64±0.8 | 49 | -1% *^rrf-3;^ ^daf-2^* | 50/75 | 0.665 *^rrf-3;^ ^daf-2^* |  |
| *rrf-3(pk1426); daf-2(e1370) unc-10* RNAi | 44.57±0.8 | 49 | 1% *^rrf-3;^ ^daf-2^* | 50/75 | 0.9208 *^rrf-3;^ ^daf-2^* |  |
| *rrf-3(pk1426); daf-2(e1370) alp-1* RNAi | 42.61±0.8 | 46 | -4% *^rrf-3;^ ^daf-2^* | 46/75 | 0.1154 *^rrf-3;^ ^daf-2^* |  |
| *rrf-3(pk1426); daf-2(e1370) pxf-1* RNAi | 43.46±0.8 | 49 | -2% *^rrf-3;^ ^daf-2^* | 39/50 | 0.4395 *^rrf-3;^ ^daf-2^* |  |
| *rrf-3(pk1426)* control RNAi | 33.23±1.1 | 39 |  | 38/75 |  |  |
| *rrf-3(pk1426) daf-16* RNAi | 15.39±0.3 | 18 | -54% | 56/75 | <0.0001 |  |
| *rrf-3(pk1426) cla-1* RNAi | 29.58±1.0 | 33 | -11% | 45/75 | 0.011 |  |
| *rrf-3(pk1426) mpz-4* RNAi | 31.27±0.9 | 37 | -6% | 41/75 | 0.0949 |  |
| *rrf-3(pk1426) frm-8* RNAi | 27.98±0.9 | 31 | -16% | 48/75 | 0.0002 |  |
| *rrf-3(pk1426) magi-1* RNAi | 27.51±1.0 | 33 | -17% | 49/75 | <0.0001 |  |
| *rrf-3(pk1426) cnk-1* RNAi | 27.68±0.8 | 31 | -17% | 53/75 | <0.0001 |  |
| *rrf-3(pk1426) mig-5* RNAi | 28.94±1.1 | 33 | -13% | 46/75 | 0.0132 |  |
| *rrf-3(pk1426) unc-10* RNAi | 30.18±0.9 | 35 | -9% | 49/75 | 0.0231 |  |
| *rrf-3(pk1426) alp-1* RNAi | 28.06±0.7 | 31 | -16% | 53/75 | 0.0001 |  |
| *rrf-3(pk1426) pxf-1* RNAi | 29.55±1.0 | 35 | -11% | 48/75 | 0.0062 |  |
| *rrf-3(pk1426)* T15H9.4 RNAi | 30.37±1.0 | 33 | -9% | 53/75 | 0.0943 |  |
| *rrf-3(pk1426); daf-2(e1370)* control RNAi | 47.14±1.3 | 54 |  | 34/45 |  |  |
| *rrf-3(pk1426); daf-2(e1370) daf-16* RNAi | 23.75±0.5 | 26 | -50% *^rrf-3;^ ^daf-2^* | 56/75 | <0.0001 *^rrf-3;^ ^daf-2^* |  |
| *rrf-3(pk1426); daf-2(e1370) gipc-2* RNAi | 48.05±0.8 | 54 | 2% *^rrf-3;^ ^daf-2^* | 62/75 | 0.6014 *^rrf-3;^ ^daf-2^* |  |
| *rrf-3(pk1426); daf-2(e1370) cla-1* RNAi | 47.74±0.9 | 51 | 1% *^rrf-3;^ ^daf-2^* | 57/75 | 0.5749 *^rrf-3;^ ^daf-2^* |  |
| *rrf-3(pk1426); daf-2(e1370) mpz-4* RNAi | 47.86±0.7 | 51 | 2% *^rrf-3;^ ^daf-2^* | 59/75 | 0.3697 *^rrf-3;^ ^daf-2^* |  |
| *rrf-3(pk1426); daf-2(e1370) frm-8* RNAi | 47.45±0.7 | 51 | 1% *^rrf-3;^ ^daf-2^* | 58/74 | 0.2334 *^rrf-3;^ ^daf-2^* |  |
| *rrf-3(pk1426); daf-2(e1370) magi-1* RNAi | 44.99±0.9 | 51 | -5% *^rrf-3;^ ^daf-2^* | 60/75 | 0.0195 *^rrf-3;^ ^daf-2^* |  |
| *rrf-3(pk1426); daf-2(e1370) cnk-1* RNAi | 46.21±1.1 | 51 | -2% *^rrf-3;^ ^daf-2^* | 55/75 | 0.24 *^rrf-3;^ ^daf-2^* |  |
| *rrf-3(pk1426); daf-2(e1370) mig-5* RNAi | 47.41±0.9 | 51 | 1% *^rrf-3;^ ^daf-2^* | 59/75 | 0.3117 *^rrf-3;^ ^daf-2^* |  |
| *rrf-3(pk1426); daf-2(e1370) unc-10* RNAi | 48.64±0.7 | 54 | 3% *^rrf-3;^ ^daf-2^* | 58/75 | 0.6359 *^rrf-3;^ ^daf-2^* |  |
| *rrf-3(pk1426); daf-2(e1370) alp-1* RNAi | 45.25±1.0 | 51 | -4% *^rrf-3;^ ^daf-2^* | 38/50 | 0.0316 *^rrf-3;^ ^daf-2^* |  |
| *rrf-3(pk1426); daf-2(e1370) pxf-1* RNAi | 48.28±0.9 | 54 | 2% *^rrf-3;^ ^daf-2^* | 61/75 | 0.9914 *^rrf-3;^ ^daf-2^* |  |
| *rrf-3(pk1426)* control RNAi | 30.51±0.9 | 36 |  | 55/75 |  |  |
| *rrf-3(pk1426) daf-16* RNAi | 15.42±0.3 | 18 | -49% | 54/75 | <0.0001 |  |
| *rrf-3(pk1426)* T19B10.5 RNAi | 29.13±1.0 | 34 | -5% | 59/75 | 0.4193 |  |
| *rrf-3(pk1426) mics-1* RNAi | 29.17±1.1 | 36 | -4% | 58/75 | 0.5906 |  |
| *rrf-3(pk1426) smz-2* RNAi | 30.34±1.0 | 34 | -1% | 56/75 | 0.9317 |  |
| *rrf-3(pk1426) par-6* RNAi | 23.47±0.9 | 29 | -23% | 65/75 | <0.0001 |  |
| *rrf-3(pk1426) frm-5.2* RNAi | 30.17±0.9 | 36 | -1% | 65/75 | 0.9808 |  |
| *rrf-3(pk1426)* Y42H9AR.1 RNAi | 29.79±1.1 | 36 | -2% | 60/75 | 0.8786 |  |
| *rrf-3(pk1426) mpz-6* RNAi | 28.28±0.8 | 34 | -7% | 56/75 | 0.0293 |  |
| *rrf-3(pk1426) lin-7* RNAi | 26.76±1.0 | 34 | -12% | 61/75 | 0.0096 |  |
| *rrf-3(pk1426); daf-2(e1370)* control RNAi | 45.4±0.7 | 49 |  | 41/75 |  |  |
| *rrf-3(pk1426); daf-2(e1370) daf-16* RNAi | 25.2±0.6 | 28 | -44% *^rrf-3;^ ^daf-2^* | 37/75 | <0.0001 *^rrf-3;^ ^daf-2^* |  |
| *rrf-3(pk1426); daf-2(e1370)* T15H9.4 RNAi | 46.37±0.6 | 49 | 2% *^rrf-3;^ ^daf-2^* | 26/50 | 0.8038 *^rrf-3;^ ^daf-2^* |  |
| *rrf-3(pk1426); daf-2(e1370)* T19B10.5 RNAi | 45.41±0.8 | 49 | 0% *^rrf-3;^ ^daf-2^* | 30/75 | 0.8182 *^rrf-3;^ ^daf-2^* |  |
| *rrf-3(pk1426); daf-2(e1370) mics-1* RNAi | 43.95±0.9 | 49 | -3% *^rrf-3;^ ^daf-2^* | 46/75 | 0.3463 *^rrf-3;^ ^daf-2^* |  |
| *rrf-3(pk1426); daf-2(e1370) smz-2* RNAi i | 42.05±0.7 | 46 | -7% *^rrf-3;^ ^daf-2^* | 61/75 | 0.0024 *^rrf-3;^ ^daf-2^* |  |
| *rrf-3(pk1426); daf-2(e1370) par-6* RNAi | 41.6±0.9 | 46 | -8% *^rrf-3;^ ^daf-2^* | 50/75 | 0.0021 *^rrf-3;^ ^daf-2^* |  |
| *rrf-3(pk1426); daf-2(e1370) frm-5.2* RNAi | 41.09±0.7 | 46 | -9% *^rrf-3;^ ^daf-2^* | 60/75 | <0.0001 *^rrf-3;^ ^daf-2^* |  |
| *rrf-3(pk1426); daf-2(e1370)* Y42H9AR.1 RNAi | 41.79±0.8 | 46 | -8% *^rrf-3;^ ^daf-2^* | 53/75 | 0.0008 *^rrf-3;^ ^daf-2^* |  |
| *rrf-3(pk1426); daf-2(e1370) mpz-6* RNAi | 42.77±0.7 | 46 | -6% *^rrf-3;^ ^daf-2^* | 58/75 | 0.0082 *^rrf-3;^ ^daf-2^* |  |
| *rrf-3(pk1426)* control RNAi | 31.38±0.9 | 36 |  | 53/76 |  |  |
| *rrf-3(pk1426) daf-16* RNAi | 15±0.4 | 17 | -52% | 60/75 | <0.0001 |  |
| *rrf-3(pk1426)* T19B10.5 RNAi | 30.26±0.7 | 34 | -4% | 42/76 | 0.0886 |  |
| *rrf-3(pk1426) mics-1* RNAi | 27.92±0.9 | 32 | -11% | 50/74 | 0.0011 |  |
| *rrf-3(pk1426) smz-2* RNAi | 28.43±0.9 | 34 | -9% | 52/75 | 0.0046 |  |
| *rrf-3(pk1426) par-6* RNAi | 23.63±0.7 | 28 | -25% | 57/75 | <0.0001 |  |
| *rrf-3(pk1426) frm-5.2* RNAi | 27.93±0.9 | 32 | -11% | 49/75 | 0.0046 |  |
| *rrf-3(pk1426)* Y42H9AR.1 RNAi | 28.93±0.8 | 34 | -8% | 52/75 | 0.0139 |  |
| *rrf-3(pk1426) mpz-6* RNAi | 29.13±1.0 | 32 | -7% | 34/55 | 0.0185 |  |
| *rrf-3(pk1426) lin-7* RNAi | 26.96±0.9 | 32 | -14% | 50/75 | <0.0001 |  |
| *rrf-3(pk1426); daf-2(e1370)* control RNAi | 50.16±1.1 | 56 |  | 62/77 |  |  |
| *rrf-3(pk1426); daf-2(e1370) daf-16* RNAi | 23.68±0.7 | 26 | -53% *^rrf-3;^ ^daf-2^* | 54/75 | <0.0001 *^rrf-3;^ ^daf-2^* |  |
| *rrf-3(pk1426); daf-2(e1370)* T15H9.4 RNAi | 50.86±0.9 | 56 | 1% *^rrf-3;^ ^daf-2^* | 65/75 | 0.901 *^rrf-3;^ ^daf-2^* |  |
| *rrf-3(pk1426); daf-2(e1370)* T19B10.5 RNAi | 53.08±1.1 | 59 | 6% *^rrf-3;^ ^daf-2^* | 66/75 | 0.0385 *^rrf-3;^ ^daf-2^* |  |
| *rrf-3(pk1426); daf-2(e1370) mics-1* RNAi | 48.3±1.0 | 53 | -4% *^rrf-3;^ ^daf-2^* | 65/75 | 0.0683 *^rrf-3;^ ^daf-2^* |  |
| *rrf-3(pk1426); daf-2(e1370) smz-2* RNAi i | 51.99±1.0 | 59 | 4% *^rrf-3;^ ^daf-2^* | 62/75 | 0.2726 *^rrf-3;^ ^daf-2^* |  |
| *rrf-3(pk1426); daf-2(e1370) par-6* RNAi | 48.2±0.8 | 53 | -4% *^rrf-3;^ ^daf-2^* | 61/75 | 0.014 *^rrf-3;^ ^daf-2^* |  |
| *rrf-3(pk1426); daf-2(e1370) frm-5.2* RNAi | 47.13±1.0 | 53 | -6% *^rrf-3;^ ^daf-2^* | 60/75 | 0.0223 *^rrf-3;^ ^daf-2^* |  |
| *rrf-3(pk1426); daf-2(e1370)* Y42H9AR.1 RNAi | 48.59±1.1 | 56 | -3% *^rrf-3;^ ^daf-2^* | 67/75 | 0.182 *^rrf-3;^ ^daf-2^* |  |
| *rrf-3(pk1426); daf-2(e1370) mpz-6* RNAi | 52.5±0.8 | 56 | 5% *^rrf-3;^ ^daf-2^* | 62/75 | 0.3778 *^rrf-3;^ ^daf-2^* |  |
| *rrf-3(pk1426)* control RNAi | 27.38±1.2 | 36 |  | 57/75 |  |  |
| *rrf-3(pk1426) daf-16* RNAi | 17.11±0.6 | 20 | -38% | 51/75 | <0.0001 |  |
| *rrf-3(pk1426) magu-1* RNAi | 29.88±1.0 | 34 | 9% | 48/75 | 0.2939 |  |
| *rrf-3(pk1426)* Y57G11C.22 RNAi | 29.92±0.9 | 36 | 9% | 47/75 | 0.5418 |  |
| *rrf-3(pk1426)* ZK849.1 RNAi | 28.39±1.0 | 34 | 4% | 51/75 | 0.9807 |  |
| *rrf-3(pk1426) gopc-1* RNAi | 29.62±1.1 | 35 | 8% | 49/75 | 0.2759 |  |
| *rrf-3(pk1426) magu-3* RNAi | 29.08±1.0 | 34 | 6% | 52/75 | 0.6492 |  |
| *rrf-3(pk1426); daf-2(e1370)* control RNAi | 50.68±1.4 | 57 |  | 35/75 |  |  |
| *rrf-3(pk1426); daf-2(e1370) daf-16* RNAi | 28.57±0.7 | 32 | -44% *^rrf-3;^ ^daf-2^* | 42/75 | <0.0001 *^rrf-3;^ ^daf-2^* |  |
| *rrf-3(pk1426); daf-2(e1370) lin-7* RNAi | 49.59±1.2 | 57 | -2% *^rrf-3;^ ^daf-2^* | 41/76 | 0.4948 *^rrf-3;^ ^daf-2^* |  |
| *rrf-3(pk1426); daf-2(e1370) magu-1* RNAi | 48.46±1.3 | 57 | -4% *^rrf-3;^ ^daf-2^* | 43/75 | 0.3229 *^rrf-3;^ ^daf-2^* |  |
| *rrf-3(pk1426); daf-2(e1370)* Y57G11C.22 RNAi | 52.01±1.4 | 59 | 3% *^rrf-3;^ ^daf-2^* | 36/75 | 0.4555 *^rrf-3;^ ^daf-2^* |  |
| *rrf-3(pk1426); daf-2(e1370)* ZK849.1 RNAi | 50.24±0.8 | 54 | -1% *^rrf-3;^ ^daf-2^* | 43/75 | 0.2498 *^rrf-3;^ ^daf-2^* |  |
| *rrf-3(pk1426); daf-2(e1370) gopc-1* RNAi | 51.13±1.9 | 56 | 1% *^rrf-3;^ ^daf-2^* | 9/50 | 0.9128 *^rrf-3;^ ^daf-2^* |  |
| *rrf-3(pk1426); daf-2(e1370) magu-3* RNAi | 52.01±1.2 | 57 | 3% *^rrf-3;^ ^daf-2^* | 48/75 | 0.5111 *^rrf-3;^ ^daf-2^* |  |
| *rrf-3(pk1426)* control RNAi | 25.68±0.7 | 29 |  | 51/75 |  |  |
| *rrf-3(pk1426) daf-16* RNAi | 14.95±0.4 | 19 | -42% | 50/75 | <0.0001 |  |
| *rrf-3(pk1426) magu-1* RNAi | 27.91±0.8 | 31 | 9% | 41/77 | 0.0325 |  |
| *rrf-3(pk1426)* Y57G11C.22 RNAi | 26.1±0.7 | 29 | 2% | 50/75 | 0.6455 |  |
| *rrf-3(pk1426)* ZK849.1 RNAi | 27.67±0.8 | 31 | 8% | 50/75 | 0.063 |  |
| *rrf-3(pk1426) gopc-1* RNAi | 30.44±0.6 | 34 | 19% | 57/75 | <0.0001 |  |
| *rrf-3(pk1426) magu-3* RNAi | 28.26±0.8 | 31 | 10% | 40/75 | 0.0131 |  |
| *rrf-3(pk1426); daf-2(e1370)* control RNAi | 48.6±1.0 | 56 |  | 57/75 |  |  |
| *rrf-3(pk1426); daf-2(e1370) daf-16* RNAi | 27.28±0.7 | 33 | -44% *^rrf-3;^ ^daf-2^* | 55/74 | <0.0001 *^rrf-3;^ ^daf-2^* |  |
| *rrf-3(pk1426); daf-2(e1370) lin-7* RNAi | 48.97±0.9 | 56 | 1% *^rrf-3;^ ^daf-2^* | 60/75 | 0.8925 *^rrf-3;^ ^daf-2^* |  |
| *rrf-3(pk1426); daf-2(e1370) magu-1* RNAi | 47.68±0.9 | 52 | -2% *^rrf-3;^ ^daf-2^* | 54/75 | 0.3108 *^rrf-3;^ ^daf-2^* |  |
| *rrf-3(pk1426); daf-2(e1370)* Y57G11C.22 RNAi | 46.57±1.0 | 52 | -4% *^rrf-3;^ ^daf-2^* | 57/75 | 0.237 *^rrf-3;^ ^daf-2^* |  |
| *rrf-3(pk1426); daf-2(e1370)* ZK849.1 RNAi | 47±0.8 | 52 | -3% *^rrf-3;^ ^daf-2^* | 56/75 | 0.0721 *^rrf-3;^ ^daf-2^* |  |
| *rrf-3(pk1426); daf-2(e1370) gopc-1* RNAi | 52.06±1.1 | 59 | 7% *^rrf-3;^ ^daf-2^* | 54/75 | 0.0021 *^rrf-3;^ ^daf-2^* |  |
| *rrf-3(pk1426); daf-2(e1370) magu-3* RNAi | 48.27±0.9 | 52 | -1% *^rrf-3;^ ^daf-2^* | 64/75 | 0.7967 *^rrf-3;^ ^daf-2^* |  |

Individual lifespan assay sets are distinguished by solid lines, and dashed lines divide conditions. All of lifespan assays were performed by transferring L4 worms from control RNAi to corresponding RNAi conditions. Percent changes (%) and *p*–values were calculated within one experimental set. *p*–values were calculated with log-rank (Mantel-Cox method) test.

*^rrf-3;^ ^daf-2^* indicates the comparison between each experimental condition and *rrf-3(pk1426); daf-2(e1370)* control RNAi condition.

**TABLE S3** Analysis of lifespan assay results other than RNAi screens

| **Strain** | **Mean lifespan ±s.e.m. (days)** | **75th percentile** | **% change** | **Number of animals that died/total** | ***p* value vs. control** | **Figure in text** |
| --- | --- | --- | --- | --- | --- | --- |
| WT | 20.13±0.9 | 26 |  | 44/50 |  |  |
| *kin-4(tm1049)* | 12.34±0.6 | 17 | -39% | 80/100 | <0.0001 |  |
| *daf-2(e1370)* | 35.80±1.0 | 40 | 78% | 67/75 | <0.0001 |  |
| *daf-2(e1370); kin-4(tm1049)* | 26.86±1.2 | 37 | -25% *^daf-2^* | 94/100 | 0.0001 *^daf-2^* |  |
| WT | 15.71±0.5 | 20 |  | 78/100 |  |  |
| *kin-4(tm1049)* | 12.02±0.3 | 15 | -23% | 159/200 | <0.0001 |  |
| *daf-2(e1370)* | 31.51±1.3 | 42 | 101% | 76/100 | <0.0001 |  |
| *daf-2(e1370); kin-4(tm1049)* | 25.81±1.2 | 32 | -18% *^daf-2^* | 89/100 | 0.001 *^daf-2^* |  |
| WT | 16.92±0.5 | 20 |  | 91/100 |  |  |
| *kin-4(tm1049)* | 16.93±0.4 | 20 | 0% | 51/100 | 0.7797 |  |
| *daf-2(e1370)* | 36.06±1.5 | 45 | 113% | 67/100 | <0.0001 |  |
| *daf-2(e1370); kin-4(tm1049)* | 28.69±1.1 | 37 | -20% *^daf-2^* | 83/100 | <0.0001 *^daf-2^* |  |
| WT | 22.75±0.4 | 26 |  | 98/120 |  |  |
| *kin-4(tm1049)* | 16.47±0.5 | 20 | -28% | 68/120 | <0.0001 |  |
| *daf-2(e1370)* | 39.22±1.0 | 46 | 72% | 100/118 | <0.0001 |  |
| *daf-2(e1370); kin-4(tm1049)* | 29.23±1.3 | 40 | -25% *^daf-2^* | 114/120 | <0.0001 *^daf-2^* |  |
| WT | 22.09±0.4 | 24 |  | 79/120 |  | Figure S1a |
| *kin-4(tm1049)* | 16.02±0.6 | 20 | -27% | 48/76 | <0.0001 | Figure S1a |
| *daf-2(e1370)* | 41.25±1.4 | 53 | 87% | 85/120 | <0.0001 | Figure S1a |
| *daf-2(e1370); kin-4(tm1049)* | 26.81±1.0 | 31 | -35% *^daf-2^* | 107/120 | <0.0001 *^daf-2^* | Figure S1a |
| WT | 20.69±0.5 | 24 |  | 85/120 |  |  |
| *kin-4(tm1049)* | 15.03±0.5 | 19 | -27% | 86/119 | <0.0001 |  |
| *daf-2(e1370)* | 35.23±0.9 | 41 | 70% | 99/120 | <0.0001 |  |
| *daf-2(e1370); kin-4(tm1049)* | 26.36±1.0 | 36 | -25% *^daf-2^* | 109/122 | <0.0001 *^daf-2^* |  |
| WT^α^ | 21.71±0.5 | 26 |  | 109/120 |  |  |
| *kin-4(tm1049)*^α^ | 16.73±0.5 | 23 | -23% | 97/120 | <0.0001 |  |
| *daf-2(e1370)*^α^ | 41.75±1.4 | 54 | 92% | 100/120 | <0.0001 |  |
| *daf-2(e1370); kin-4(tm1049)*^α^ | 32.06±1.6 | 43 | -23% *^daf-2^* | 73/120 | <0.0001 *^daf-2^* |  |
| WT^α^ | 17.68±0.6 | 22 |  | 74/120 |  | Figure 2a |
| *kin-4(tm1049* ^α^ | 15.51±0.5 | 18 | -12% | 54/90 | 0.0015 | Figure 2a |
| *daf-2(e1370)*^α^ | 41.42±1.7 | 51 | 134% | 62/90 | <0.0001 | Figure 2a |
| *daf-2(e1370); kin-4(tm1049)*^α^ | 21.71±1.5 | 27 | -48% *^daf-2^* | 66/120 | <0.0001 *^daf-2^* | Figure 2a |
| WT^α^ | 18±1.0 | 19 |  | 30/83 |  |  |
| *kin-4(tm1049)*^α^ | 16.65±0.6 | 21 | -8% | 75/118 | 0.3064 |  |
| *daf-2(e1370)*^α^ | 39.33±1.0 | 44 | 119% | 52/120 | <0.0001 |  |
| *daf-2(e1370); kin-4(tm1049)*^α^ | 23.07±1.6 | 34 | -41% *^daf-2^* | 64/120 | <0.0001 *^daf-2^* |  |
| WT | 18.62±0.4 | 21 |  | 106/114 |  |  |
| *kin-4(nj170)* | 17.14±0.3 | 18 | -8% | 113/119 | 0.0025 |  |
| *daf-2(e1370)* | 31.17±1.3 | 46 | 67% | 103/120 | <0.0001 |  |
| *daf-2(e1370); kin-4(nj170)* | 17.91±1.3 | 23 | -43% *^daf-2^* | 74/102 | <0.0001 *^daf-2^* |  |
| WT | 14.81±0.3 | 17 |  | 104/120 |  | Figure S1b |
| *kin-4(nj170)* | 14.91±0.2 | 17 | 1% | 93/120 | 0.8229 | Figure S1b |
| *daf-2(e1370)* | 35.6±1.4 | 44 | 140% | 76/120 | <0.0001 | Figure S1b |
| *daf-2(e1370); kin-4(nj170)* | 12.63±0.7 | 15 | -65% *^daf-2^* | 38/63 | <0.0001 *^daf-2^* | Figure S1b |
| WT^α^ | 17.12±0.5 | 21 |  | 93/160 |  | Figure 2b |
| *kin-4(nj170)*^α^ | 15.42±0.5 | 18 | -10% | 51/160 | 0.0204 | Figure 2b |
| *daf-2(e1370)*^α^ | 42.16±1.5 | 54 | 146% | 101/204 | <0.0001 | Figure 2b |
| *daf-2(e1370); kin-4(nj170)*^α^ | 20.04±1.0 | 25 | -52% *^daf-2^* | 70/307 | <0.0001 *^daf-2^* | Figure 2b |
| WT^α^ | 17.35±0.5 | 21 |  | 128/160 |  |  |
| *kin-4(nj170)*^α^ | 14.04±0.8 | 21 | -19% | 70/160 | 0.0003 |  |
| *daf-2(e1370)*^α^ | 38.67±1.3 | 49 | 123% | 108/200 | <0.0001 |  |
| *daf-2(e1370); kin-4(nj170)*^α^ | 17.09±0.7 | 20 | -56% *^daf-2^* | 87/240 | <0.0001 *^daf-2^* |  |
| WT control RNAi | 24.37±0.5 | 27 |  | 105/120 |  | Figure S1d |
| *kin-4(tm1049)* control RNAi | 18.51±0.4 | 22 | -24% | 106/120 | <0.0001 | Figure S1d |
| WT *daf-2* RNAi | 37.46±1.2 | 48 | 54% | 77/90 | <0.0001 | Figure S1d |
| *kin-4(tm1049) daf-2* RNAi | 26.64±0.7 | 33 | -29% *^daf-2^* | 108/130 | <0.0001 *^daf-2^* | Figure S1d |
| WT control RNAi | 21±0.4 | 25 |  | 107/120 |  |  |
| *kin-4(tm1049)* control RNAi | 14.98±0.3 | 18 | -29% | 106/120 | <0.0001 |  |
| WT *daf-2* RNAi | 35.6±1.4 | 51 | 70% | 108/120 | <0.0001 |  |
| *kin-4(tm1049) daf-2* RNAi | 20.26±0.6 | 25 | -43% *^daf-2^* | 110/120 | <0.0001 *^daf-2^* |  |
| WT control RNAi | 23.19±0.5 | 28 |  | 107/119 |  |  |
| *kin-4(tm1049)* control RNAi | 16.01±0.4 | 20 | -31% | 92/120 | <0.0001 |  |
| WT *daf-2* RNAi | 37.01±1.1 | 46 | 60% | 97/120 | <0.0001 |  |
| *kin-4(tm1049) daf-2* RNAi | 25.28±1.0 | 31 | -32% *^daf-2^* | 98/120 | <0.0001 *^daf-2^* |  |
| WT control RNAi^α^ | 24.22±0.6 | 31 |  | 86/120 |  |  |
| *kin-4(tm1049)* control RNAi^α^ | 19.81±0.5 | 24 | -18% | 108/119 | <0.0001 |  |
| WT *daf-2* RNAi^α^ | 42.97±2.7 | 58 | 77% | 37/81 | <0.0001 |  |
| *kin-4(tm1049) daf-2* RNAi^α^ | 29.1±1.1 | 38 | -32% *^daf-2^* | 106/120 | <0.0001 *^daf-2^* |  |
| WT control RNAi^α^ | 20.34±0.6 | 25 |  | 78/120 |  |  |
| *kin-4(tm1049)* control RNAi^α^ | 15.46±0.5 | 19 | -24% | 76/120 | <0.0001 |  |
| WT *daf-2* RNAi^α^ | 35.78±2.0 | 49 | 76% | 50/90 | <0.0001 |  |
| *kin-4(tm1049) daf-2* RNAi^α^ | 26.13±1.1 | 34 | -27% *^daf-2^* | 88/120 | <0.0001 *^daf-2^* |  |
| WT control RNAi^α^ | 22.38±0.6 | 28 |  | 91/120 |  | Figure S1e |
| *kin-4(tm1049)* control RNAi^α^ | 17.68±0.4 | 20 | -21% | 76/119 | <0.0001 | Figure S1e |
| WT *daf-2* RNAi^α^ | 35.66±1.6 | 45 | 59% | 72/120 | <0.0001 | Figure S1e |
| *kin-4(tm1049) daf-2* RNAi^α^ | 25.2±0.9 | 33 | -29% *^daf-2^* | 96/120 | <0.0001 *^daf-2^* | Figure S1e |
| WT | 20.68±0.4 | 25 |  | 101/120 |  | Figure S1c |
| *gipc-1(hc192); gipc-2(ok1172)* | 21.23±0.6 | 27 | 3% | 85/120 | 0.0418 | Figure S1c |
| *daf-2(e1370)* | 34.19±1.4 | 49 | 65% | 94/120 | <0.0001 | Figure S1c |
| *daf-2(e1370) gipc-1(hc192); gipc-2(ok1172)* | 29.1±1.3 | 43 | -15% *^daf-2^* | 113/120 | 0.0077 *^daf-2^* | Figure S1c |
| WT | 19.38±0.4 | 22 |  | 99/120 |  |  |
| *gipc-1(hc192); gipc-2(ok1172)* | 19.34±0.4 | 22 | 0% | 90/120 | 0.9452 |  |
| *daf-2(e1370)* | 35.33±0.9 | 44 | 82% | 107/120 | <0.0001 |  |
| *daf-2(e1370) gipc-1(hc192); gipc-2(ok1172)* | 30.1±0.7 | 34 | -15% *^daf-2^* | 107/120 | <0.0001 *^daf-2^* |  |
| WT | 17.97±0.4 | 21 |  | 106/120 |  |  |
| *gipc-1(hc192); gipc-2(ok1172)* | 17.89±0.6 | 21 | 0% | 57/120 | 0.9087 |  |
| *daf-2(e1370)* | 40.07±1.5 | 52 | 123% | 100/120 | <0.0001 |  |
| *daf-2(e1370) gipc-1(hc192); gipc-2(ok1172)* | 35.4±1.6 | 50 | -12% *^daf-2^* | 99/120 | 0.2165 *^daf-2^* |  |
| WT | 21.22±0.6 | 25 |  | 99/120 |  |  |
| *kin-4(tm1049)* | 17.57±0.5 | 22 | -17% | 89/123 | <0.0001 |  |
| *osm-5(p813)* | 28.8±1.1 | 37 | 36% | 83/200 | <0.0001 |  |
| *kin-4(tm1049); osm-5(p813)* | 20.79±0.6 | 25 | -28% *^osm-5^* | 93/200 | <0.0001 *^osm-5^* |  |
| WT | 16.74±0.5 | 20 |  | 76/90 |  | Figure 2c |
| *kin-4(tm1049)* | 16.31±0.5 | 20 | -3% | 93/117 | 0.7382 | Figure 2c |
| *osm-5(p813)* | 24.14±0.7 | 30 | 44% | 101/200 | <0.0001 | Figure 2c |
| *kin-4(tm1049); osm-5(p813)* | 16.99±0.5 | 20 | -30% *^osm-5^* | 104/120 | <0.0001 *^osm-5^* | Figure 2c |
| WT | 16.31±0.4 | 18 |  | 107/118 |  | Figure 2d |
| *kin-4(tm1049)* | 14.92±0.3 | 18 | -9% | 128/130 | 0.0104 | Figure 2d |
| *eat-2(ad1116)* | 20.12±0.5 | 24 | 23% | 96/120 | <0.0001 | Figure 2d |
| *eat-2(ad1116); kin-4(tm1049)* | 21.27±0.4 | 24 | 6% *^eat-2^* | 116/140 | 0.1551 *^eat-2^* | Figure 2d |
| WT | 16.54±0.4 | 19 |  | 115/130 |  |  |
| *kin-4(tm1049)* | 15.71±0.3 | 19 | -5% | 152/160 | 0.0752 |  |
| *eat-2(ad1116)* | 19.15±0.5 | 21 | 16% | 94/120 | 0.0017 |  |
| *eat-2(ad1116); kin-4(tm1049)* | 20.13±0.4 | 25 | 5% *^eat-2^* | 121/140 | 0.1545 *^eat-2^* |  |
| *yhEx246[odr-1p::rfp]* | 21.13±0.6 | 25 |  | 93/120 |  |  |
| *yhEx244[kin-4p::kin-4a::gfp; odr-1p::rfp]* line1 | 26.51±0.6 | 31 | 25% | 97/120 | <0.0001 |  |
| *yhEx245[kin-4p::kin-4a::gfp; odr-1p::rfp]* line2 | 25.82±0.6 | 31 | 22% | 95/120 | <0.0001 |  |
| *yhEx246[odr-1p::rfp]* | 18.67±0.5 | 24 |  | 99/120 |  | Figure S4c |
| *yhEx244[kin-4p::kin-4a::gfp; odr-1p::rfp]* line1 | 20.02±0.6 | 24 | 7% | 65/120 | 0.2242 | Figure S4c |
| *yhEx245[kin-4p::kin-4a::gfp; odr-1p::rfp]* line2 | 19.64±0.5 | 24 | 5% | 83/120 | 0.3256 | Figure S4c |
| WT | 22.64±0.5 | 26 |  | 107/120 |  |  |
| *njEx683[kin-4p::kin-4::gfp; ges-1p::TagRFP]* | 25.6±0.6 | 29 | 13% | 111/120 | <0.0001 |  |
| WT | 20.51±0.5 | 26 |  | 112/120 |  | Figure 3e |
| *njEx683[kin-4p::kin-4::gfp; ges-1p::TagRFP]* | 21.66±0.6 | 26 | 6% | 107/120 | 0.0176 | Figure 3e |
| WT | 19.61±0.6 | 24 |  | 96/120 |  |  |
| *njEx683[kin-4p::kin-4::gfp; ges-1p::TagRFP]* | 20.4±0.7 | 27 | 4% | 82/120 | 0.2259 |  |
| *daf-2(e1370)* | 44.01±1.4 | 56 |  | 98/120 |  | Figure 3d, Figure 6b-d |
| *daf-2(e1370); kin-4(tm1049); yhEx314[odr-1p::rfp]* | 28.37±1.3 | 37 | -36% | 86/90 | <0.0001 | Figure 3d |
| *daf-2(e1370); kin-4(tm1049); yhEx322[kin-4p::kin-4a::gfp; odr-1p::rfp]* | 38.68±1.4 | 52 | -12%  (36% *^daf-2; kin-4; odr-1p::rfp^*) | 107/120 | 0.0025  (<0.0001 *^daf-2; kin-4; odr-1p::rfp^*) | Figure 3d |
| *daf-2(e1370); daf-18(nr2037); yhEx318[odr-1p::rfp]* | 17.2±0.4 | 20 | -61% | 106/111 | <0.0001 |  |
| *daf-2(e1370); daf-18(nr2037); yhEx313[daf-18p::mCherry::daf-18 WT; odr-1p::rfp]* | 40.96±1.6 | 55 | -7%  (138% *^daf-2; daf-18; odr-1p::rfp^*) | 93/120 | 0.2022  (<0.0001 *^daf-2; daf-18; odr-1p::rfp^*) | Figure 6b |
| *daf-2(e1370); daf-18(nr2037); yhEx315[daf-18p::mCherry::daf-18 Δ4C; odr-1p::rfp]* line 1 | 33.88±1.2 | 46 | -23%  (97% *^daf-2; daf-18; odr-1p::rfp^*) | 106/121 | <0.0001  (<0.0001 *^daf-2; daf-18; odr-1p::rfp^*) | Figure 6c |
| *daf-2(e1370); daf-18(nr2037); yhEx315[daf-18p::mCherry::daf-18 Δ4C; odr-1p::rfp]* line 2 | 37.81±1.1 | 44 | -14%  (120% *^daf-2; daf-18; odr-1p::rfp^*) | 106/120 | <0.0001  (<0.0001 *^daf-2; daf-18; odr-1p::rfp^*) | Figure 6c |
| *daf-2(e1370); daf-18(nr2037); yhEx315[daf-18p::mCherry::daf-18 Δ12C; odr-1p::rfp]* | 24.81±1.1 | 32 | -44%  (44% *^daf-2; daf-18; odr-1p::rfp^*) | 101/121 | <0.0001  (<0.0001 *^daf-2; daf-18; odr-1p::rfp^*) | Figure 6d |
| *daf-2(e1370); daf-18(nr2037)* | 19.06±0.3 | 24 | -57%  (11% *^daf-2; daf-18; odr-1p::rfp^*) | 100/120 | <0.0001  (0.0003 *^daf-2; daf-18; odr-1p::rfp^*) | Figure 6b-d |
| *daf-2(e1370)* | 43.65±1.4 | 54 |  | 75/90 |  |  |
| *daf-2(e1370); kin-4(tm1049); yhEx314[odr-1p::rfp]* | 26.93±1.1 | 34 | -38% | 102/119 | <0.0001 |  |
| *daf-2(e1370); kin-4(tm1049); yhEx322[kin-4p::kin-4a::gfp; odr-1p::rfp]* | 34.69±1.3 | 48 | -21%  (29% *^daf-2; kin-4; odr-1p::rfp^*) | 104/121 | 0.0001  (<0.0001 *^daf-2; kin-4; odr-1p::rfp^*) |  |
| *daf-2(e1370)* | 45.86±1.3 | 55 |  | 95/120 |  |  |
| *daf-2(e1370); daf-18(nr2037); yhEx318[odr-1p::rfp]* | 20.89±0.5 | 24 | -54% | 111/120 | <0.0001 |  |
| *daf-2(e1370); daf-18(nr2037); yhEx313[daf-18p::mCherry::daf-18 WT; odr-1p::rfp]* | 39.43±1.8 | 57 | -14%  (89% *^daf-2; daf-18; odr-1p::rfp^*) | 99/119 | 0.2083  (<0.0001 *^daf-2; daf-18; odr-1p::rfp^*) |  |
| *daf-2(e1370); daf-18(nr2037); yhEx315[daf-18p::mCherry::daf-18 Δ4C; odr-1p::rfp]* line 1 | 40.34±1.5 | 53 | -12%  (93% *^daf-2; daf-18; odr-1p::rfp^*) | 95/120 | 0.0301  (<0.0001 *^daf-2; daf-18; odr-1p::rfp^*) |  |
| *daf-2(e1370); daf-18(nr2037); yhEx315[daf-18p::mCherry::daf-18 Δ4C; odr-1p::rfp]* line 2 | 35.01±1.6 | 47 | -24%  (68% *^daf-2; daf-18; odr-1p::rfp^*) | 96/120 | 0.0001  (<0.0001 *^daf-2; daf-18; odr-1p::rfp^*) |  |
| *daf-2(e1370); daf-18(nr2037); yhEx315[daf-18p::mCherry::daf-18 Δ12C; odr-1p::rfp]* | 25.54±1.1 | 32 | -44%  (22% *^daf-2; daf-18; odr-1p::rfp^*) | 107/120 | <0.0001  (<0.0001 *^daf-2; daf-18; odr-1p::rfp^*) |  |
| *daf-2(e1370); daf-18(nr2037)* | 20.42±0.4 | 25 | -55%  (-2% *^daf-2; daf-18; odr-1p::rfp^*) | 110/120 | <0.0001  (0.7566 *^daf-2; daf-18; odr-1p::rfp^*) |  |
| *daf-2(e1370); kin-4(tm1049)* | 32.25±1.0 | 41 |  | 126/140 |  |  |
| *daf-2(e1370); kin-4(tm1049); yhEx446[rgef-1p::kin-4a::gfp; odr-1p::RFP]* | 37.62±1.3 | 50 | 17% | 118/120 | <0.0001  (0.9014 *^daf-2^*) |  |
| *daf-2(e1370); kin-4(tm1049); yhEx449[lin-26p::kin-4a::gfp; odr-1p::RFP]* | 34.1±1.0 | 41 | 6% | 115/120 | 0.2809  (0.0137 *^daf-2^*) |  |
| *daf-2(e1370); kin-4(tm1049); yhEx466[ges-1p::kin-4a::gfp; odr-1p::RFP]* | 33.31±1.2 | 44 | 3% | 111/120 | 0.1031  (0.0218 *^daf-2^*) |  |
| *daf-2(e1370); yhEx246[odr-1p::RFP]* | 36.35±1.4 | 53 | 13% | 108/120 | 0.0009 |  |
| *daf-2(e1370); kin-4(tm1049)* | 29.73±0.9 | 38 |  | 128/140 |  | Figure 3f, g and S5d |
| *daf-2(e1370); kin-4(tm1049); yhEx446[rgef-1p::kin-4a::gfp; odr-1p::RFP]* | 35.09±1.2 | 46 | 18% | 82/90 | 0.0003  (0.0494 *^daf-2^*) | Figure 3f |
| *daf-2(e1370); kin-4(tm1049); yhEx449[lin-26p::kin-4a::gfp; odr-1p::RFP]* | 29.66±0.8 | 35 | 0% | 111/120 | 0.6313  (<0.0001 *^daf-2^*) | Figure S5d |
| *daf-2(e1370); kin-4(tm1049); yhEx466[ges-1p::kin-4a::gfp; odr-1p::RFP]* | 31.6±0.9 | 40 | 6% | 100/120 | 0.5006  (<0.0001 *^daf-2^*) | Figure 3g |
| *daf-2(e1370); yhEx246[odr-1p::RFP]* | 37.26±1.1 | 50 | 25% | 117/120 | <0.0001 | Figure 3f, g and S5d |
| *sid-1(pk3321)* control RNAi | 21.97±0.6 | 25 |  | 102/117 |  | Figure S5i |
| *sid-1(pk3321) kin-4* RNAi | 22.73±0.6 | 30 | 3% | 73/90 | 0.4823 | Figure S5i |
| *daf-2(e1370); sid-1(pk3321)* control RNAi | 46.31±1.0 | 53 | 111% | 103/120 | <0.0001 | Figure S5i |
| *daf-2(e1370); sid-1(pk3321) kin-4* RNAi | 44.39±1.0 | 50 | -4% *^daf-2^* | 102/120 | 0.1073 *^daf-2^* | Figure S5i |
| *sid-1(pk3321); uIs69[myo-2p::mCherry; unc-119p::sid-1]* control RNAi | 22.34±0.5 | 25 |  | 106/120 |  | Figure 3h |
| *sid-1(pk3321); uIs69[myo-2p::mCherry; unc-119p::sid-1] kin-4* RNAi | 21.84±0.5 | 25 | -2%*^)^* | 106/120 | 0.2361 | Figure 3h |
| *daf-2(e1370); sid-1(pk3321); uIs69[myo-2p::mCherry; unc-119p::sid-1]* control RNAi | 40.11±1.0 | 47 | 80% | 100/120 | <0.0001 | Figure 3h |
| *daf-2(e1370); sid-1(pk3321); uIs69[myo-2p::mCherry; unc-119p::sid-1] kin-4* RNAi | 35.37±0.9 | 44 | -12% *^daf-2^* | 100/122 | <0.0001 *^daf-2^* | Figure 3h |
| *sid-1(pk3321)* control RNAi | 18.08±0.5 | 23 |  | 99/120 |  |  |
| *sid-1(pk3321) kin-4* RNAi | 17.49±0.5 | 23 | -3% | 79/120 | 0.362 |  |
| *daf-2(e1370); sid-1(pk3321)* control RNAi | 41.47±0.9 | 47 | 129% | 98/120 | <0.0001 |  |
| *daf-2(e1370); sid-1(pk3321) kin-4* RNAi | 42.83±0.8 | 47 | 3% *^daf-2^* | 100/120 | 0.6308 *^daf-2^* |  |
| *sid-1(pk3321); uIs69[myo-2p::mCherry; unc-119p::sid-1]* control RNAi | 19.93±0.6 | 26 |  | 100/120 |  |  |
| *sid-1(pk3321); uIs69[myo-2p::mCherry; unc-119p::sid-1] kin-4* RNAi | 17.53±0.3 | 23 | -12% | 100/120 | <0.0001 |  |
| *daf-2(e1370); sid-1(pk3321); uIs69[myo-2p::mCherry; unc-119p::sid-1]* control RNAi | 38.77±1.1 | 47 | 95% | 97/120 | <0.0001 |  |
| *daf-2(e1370); sid-1(pk3321); uIs69[myo-2p::mCherry; unc-119p::sid-1] kin-4* RNAi | 35.74±0.9 | 44 | -8% *^daf-2^* | 103/120 | 0.0013 *^daf-2^* |  |
| *rde-1(ne219)* control RNAi | 18.28±0.5 | 24 |  | 105/120 |  |  |
| *rde-1(ne219) kin-4* RNAi | 18.93±0.5 | 24 | 4% | 102/120 | 0.4539 |  |
| *daf-2(e1370); rde-1(ne219)* control RNAi | 28.97±0.9 | 36 | 59% | 81/120 | <0.0001 |  |
| *daf-2(e1370); rde-1(ne219) kin-4* RNAi | 31.91±1.0 | 39 | 10% *^daf-2^* | 72/120 | 0.0311 *^daf-2^* |  |
| *rde-1(ne219); Is[wrt-2p::rde-1; myo-2p::rfp]* control RNAi | 22.13±0.5 | 30 |  | 80/120 |  |  |
| *rde-1(ne219); Is[wrt-2p::rde-1; myo-2p::rfp] kin-4* RNAi | 22±0.5 | 30 | -1% | 98/120 | 0.7628 |  |
| *daf-2(e1370); rde-1(ne219); Is[wrt-2p::rde-1; myo-2p::rfp]* control RNAi | 30.92±0.7 | 36 | 40% | 109/120 | <0.0001 |  |
| *daf-2(e1370); rde-1(ne219); Is[wrt-2p::rde-1; myo-2p::rfp] kin-4* RNAi | 31.41±0.9 | 39 | 2% *^daf-2^* | 104/120 | 0.1385 *^daf-2^* |  |
| *rde-1(ne219); kzls9[lin-26p::nls::gfp; lin-26p::rde-1; rol-6D]* control RNAi | 22.23±0.5 | 30 |  | 94/120 |  |  |
| *rde-1(ne219); kzls9[lin-26p::nls::gfp; lin-26p::rde-1; rol-6D] kin-4* RNAi | 20.71±0.4 | 24 | -7% | 99/120 | 0.0056 |  |
| *daf-2(e1370); rde-1(ne219); kzls9[lin-26p::nls::gfp; lin-26p::rde-1; rol-6D]* control RNAi | 36.42±0.7 | 43 | 64% | 111/120 | <0.0001 |  |
| *daf-2(e1370); rde-1(ne219); kzls9[lin-26p::nls::gfp; lin-26p::rde-1; rol-6D] kin-4* RNAi | 35.78±0.8 | 43 | -2% *^daf-2^* | 104/120 | 0.4282 *^daf-2^* |  |
| *rde-1(ne219); kbls7[nhx-2p::rde-1; rol-6D]* control RNAi | 20.89±0.5 | 24 |  | 100/120 |  |  |
| *rde-1(ne219); kbls7[nhx-2p::rde-1; rol-6D] kin-4* RNAi | 18.9±0.4 | 24 | -10% | 81/120 | 0.0082 |  |
| *daf-2(e1370); rde-1(ne219); kbls7[nhx-2p::rde-1; rol-6D]* control RNAi | 34.25±0.8 | 39 | 64% | 112/120 | <0.0001 |  |
| *daf-2(e1370); rde-1(ne219); kbls7[nhx-2p::rde-1; rol-6D] kin-4* RNAi | 29.05±0.9 | 39 | -15% *^daf-2^* | 107/120 | <0.0001 *^daf-2^* |  |
| *rde-1(ne219); kzls20[hlh-1p::rde-1; sur-5p::nls::gfp]* control RNAi | 16.25±0.4 | 20 |  | 100/140 |  |  |
| *rde-1(ne219); kzls20[hlh-1p::rde-1; sur-5p::nls::gfp] kin-4* RNAi | 15.8±0.3 | 20 | -3% | 79/131 | 0.2164 |  |
| *daf-2(e1370); rde-1(ne219); kzls20[hlh-1p::rde-1; sur-5p::nls::gfp]* control RNAi | 25.62±1.0 | 36 | 58% | 105/120 | <0.0001 |  |
| *daf-2(e1370); rde-1(ne219); kzls20[hlh-1p::rde-1; sur-5p::nls::gfp] kin-4* RNAi | 27.75±1.0 | 36 | 8% *^daf-2^* | 95/121 | 0.1913 *^daf-2^* |  |
| *rde-1(ne219)* control RNAi | 19.08±0.3 | 22 |  | 97/120 |  | Figure S5j |
| *rde-1(ne219) kin-4* RNAi | 18.85±0.4 | 22 | -1% | 91/120 | 0.8115 | Figure S5j |
| *daf-2(e1370); rde-1(ne219)* control RNAi | 33.17±0.9 | 40 | 74% | 63/120 | <0.0001 | Figure S5j |
| *daf-2(e1370); rde-1(ne219) kin-4* RNAi | 33.51±0.7 | 40 | 1% *^daf-2^* | 80/120 | 0.8375 *^daf-2^* | Figure S5j |
| *rde-1(ne219); Is[wrt-2p::rde-1; myo-2p::rfp]* control RNAi | 20.67±0.4 | 25 |  | 92/120 |  | Figure S5g |
| *rde-1(ne219); Is[wrt-2p::rde-1; myo-2p::rfp] kin-4* RNAi | 20.77±0.3 | 25 | 0% | 103/120 | 0.8754 | Figure S5g |
| *daf-2(e1370); rde-1(ne219); Is[wrt-2p::rde-1; myo-2p::rfp]* control RNAi | 36.56±0.7 | 40 | 77% | 84/120 | <0.0001 | Figure S5g |
| *daf-2(e1370); rde-1(ne219); Is[wrt-2p::rde-1; myo-2p::rfp] kin-4* RNAi | 37.79±0.6 | 43 | 3% *^daf-2^* | 95/120 | 0.1065 *^daf-2^* | Figure S5g |
| *rde-1(ne219); kzls9[lin-26p::nls::gfp; lin-26p::rde-1; rol-6D]* control RNAi | 20.79±0.4 | 25 |  | 84/119 |  | Figure S5f |
| *rde-1(ne219); kzls9[lin-26p::nls::gfp; lin-26p::rde-1; rol-6D] kin-4* RNAi | 19.22±0.3 | 22 | -8% | 100/120 | 0.0007 | Figure S5f |
| *daf-2(e1370); rde-1(ne219); kzls9[lin-26p::nls::gfp; lin-26p::rde-1; rol-6D]* control RNAi | 37.4±0.8 | 43 | 80% | 84/120 | <0.0001 | Figure S5f |
| *daf-2(e1370); rde-1(ne219); kzls9[lin-26p::nls::gfp; lin-26p::rde-1; rol-6D] kin-4* RNAi | 37.69±0.6 | 43 | 1% *^daf-2^* | 91/120 | 0.6078 *^daf-2^* | Figure S5f |
| *rde-1(ne219); kbls7[nhx-2p::rde-1; rol-6D]* control RNAi | 18.96±0.4 | 22 |  | 102/120 |  | Figure 3i |
| *rde-1(ne219); kbls7[nhx-2p::rde-1; rol-6D] kin-4* RNAi | 17.62±0.4 | 22 | -7% | 89/120 | 0.0069 | Figure 3i |
| *daf-2(e1370); rde-1(ne219); kbls7[nhx-2p::rde-1; rol-6D]* control RNAi | 38.03±0.8 | 43 | 101% | 79/120 | <0.0001 | Figure 3i |
| *daf-2(e1370); rde-1(ne219); kbls7[nhx-2p::rde-1; rol-6D] kin-4* RNAi | 34.03±0.8 | 40 | -11% *^daf-2^* | 73/120 | <0.0001 *^daf-2^* | Figure 3i |
| *rde-1(ne219); kzls20[hlh-1p::rde-1; sur-5p::nls::gfp]* control RNAi | 15.65±0.4 | 19 |  | 86/140 |  | Figure S5h |
| *rde-1(ne219); kzls20[hlh-1p::rde-1; sur-5p::nls::gfp] kin-4* RNAi | 15.38±0.5 | 19 | -2% | 68/120 | 0.6279 | Figure S5h |
| *daf-2(e1370); rde-1(ne219); kzls20[hlh-1p::rde-1; sur-5p::nls::gfp]* control RNAi | 31.57±1.2 | 40 | 102% | 51/120 | <0.0001 | Figure S5h |
| *daf-2(e1370); rde-1(ne219); kzls20[hlh-1p::rde-1; sur-5p::nls::gfp] kin-4* RNAi | 31.4±1.1 | 37 | -1% *^daf-2^* | 49/120 | 0.5735 *^daf-2^* | Figure S5h |
| *daf-2(e1370); daf-18(nr2037); yhIs84[daf-18p::mCherry::daf-18 Δ12C; odr-1p::rfp]* | 33.26±0.9 | 42 |  | 120/123 |  |  |
| *daf-2(e1370); daf-18(nr2037) kin-4(tm1049); yhIs84[daf-18p::mCherry::daf-18 Δ12C; odr-1p::rfp]* | 31.08±1.0 | 38 | -7% | 107/120 | 0.1362 |  |
| *daf-2(e1370); daf-18(nr2037); yhIs84[daf-18p::mCherry::daf-18 Δ12C; odr-1p::rfp]* | 31.64±0.8 | 40 |  | 112/120 |  | Figure S9 |
| *daf-2(e1370); daf-18(nr2037) kin-4(tm1049); yhIs84[daf-18p::mCherry::daf-18 Δ12C; odr-1p::rfp]* | 23.63±0.8 | 33 | -25% | 71/110 | <0.0001 | Figure S9 |
| *daf-2(e1370); daf-18(nr2037); yhIs84[daf-18p::mCherry::daf-18 Δ12C; odr-1p::rfp]* | 28.88±1.0 | 39 |  | 119/120 |  |  |
| *daf-2(e1370); daf-18(nr2037) kin-4(tm1049); yhIs84[daf-18p::mCherry::daf-18 Δ12C; odr-1p::rfp]* | 21.49±0.7 | 27 | -26% | 93/115 | <0.0001 |  |

Solid lines distinguish different sets of lifespan assays, and different conditions were divided by dashed lines. Percent changes (%) and *p–*values were calculated within one experimental set, and *p*–values were calculated with log-rank (Mantel-Cox method) test. When statistical analysis was conducted using different controls, they were marked with superscripts.

^α^: Lifespan assays that were conducted without FUdR treatment.

**TABLE S4** Analysis of dauer formation test results

| **Strain** | **% dauer±s.e.m** | **% change of dauer formation** | **Number of animals** | ***p* value vs. control** | **Figure in text** |
| --- | --- | --- | --- | --- | --- |
| *daf-2(e1370)* at 22.5^o^C | 36.34±6.2 |  | 1915 |  | Figure 2e |
| *daf-2(e1370); kin-4(tm1049)* at 22.5^o^C | 15.00±4.8 | -59% | 1867 | 0.0142 | Figure 2e |
| *daf-2(e1370)* at 25^o^C | 100±0 |  | 778 |  | Figure S2a |
| *daf-2(e1370); kin-4(tm1049)* at 25^o^C | 100±0 | 0% | 237 | - | Figure S2a |

*p* value was calculated using Student’s *t−*test.

**TABLE S5** Analysis of stress resistance assay results

| **Strain** | **Mean survival time ±s.e.m. (hours)** | **75th percentile** | **% change** | **Number of animals that died/total** | ***p* value vs. control** | **Figure in text** |
| --- | --- | --- | --- | --- | --- | --- |
| Oxidative stress resistance (7.5 mM t-BOOH) | | | | | | |
| WT | 29.68±0.9 | 38 |  | 86/89 |  | Figure 2f |
| *kin-4(tm1049)* | 26.59±1.0 | 36 | -10% | 73/83 | 0.0054 | Figure 2f |
| *daf-2(e1370)* | 75.4±3.7 | 83 | 154% | 68/90 | <0.0001 | Figure 2f |
| *daf-2(e1370); kin-4(tm1049)* | 51.67±3.0 | 62 | -31% *^daf-2^* | 69/90 | <0.0001 *^daf-2^* | Figure 2f |
| WT | 16.63±0.5 | 22 |  | 81/90 |  |  |
| *kin-4(tm1049)* | 16.25±0.5 | 22 | -2% | 82/90 | 0.7028 |  |
| *daf-2(e1370)* | 75.22±3.6 | 89 | 352% | 34/83 | <0.0001 |  |
| *daf-2(e1370); kin-4(tm1049)* | 61.06±3.1 | 72 | -19% *^daf-2^* | 41/82 | 0.0005 *^daf-2^* |  |
| Heat stress resistance (35^o^C) | | | | | | |
| WT | 15.49±0.2 | 18 |  | 89/90 |  | Figure S2c |
| *kin-4(tm1049)* | 16.95±0.3 | 20 | 9% | 86/90 | 0.0001 | Figure S2c |
| *daf-2(e1370)* | 31.09±0.3 | 34 | 101% | 80/90 | <0.0001 | Figure S2c |
| *daf-2(e1370); kin-4(tm1049)* | 30.47±0.3 | 32 | -2% *^daf-2^* | 78/90 | 0.091 *^daf-2^* | Figure S2c |
| WT | 13.71±0.3 | 16 |  | 90/90 |  |  |
| *kin-4(tm1049)* | 13.44±0.3 | 16 | -2% | 84/90 | 0.7522 |  |
| *daf-2(e1370)* | 26.70±0.3 | 30 | 95% | 83/90 | <0.0001 |  |
| *daf-2(e1370); kin-4(tm1049)* | 25.88±0.4 | 28 | -3% *^daf-2^* | 76/90 | 0.1176 *^daf-2^* |  |
| Pathogen resistance (PA14) | | | | | | |
| WT | 81.23±2.3 | 100 |  | 76/90 |  | Figure S2b |
| *kin-4(tm1049)* | 69.73±2.6 | 76 | -14% | 62/80 | 0.0009 | Figure S2b |
| *daf-2(e1370)* | 109.32±7.3 | 138 | 35% | 73/90 | 0.0019 | Figure S2b |
| *daf-2(e1370); kin-4(tm1049)* | 152.6±9.1 | 219 | 40% *^daf-2^* | 83/90 | 0.002 *^daf-2^* | Figure S2b |
| WT | 97.41±2.8 | 114 |  | 71/85 |  |  |
| *kin-4(tm1049)* | 62.49±1.9 | 76 | -36% | 35/59 | <0.0001 |  |
| *daf-2(e1370)* | 134.77±8.9 | 188 | 38% | 61/90 | 0.0043 |  |
| *daf-2(e1370); kin-4(tm1049)* | 159.66±9.1 | 237 | 18% *^daf-2^* | 61/90 | 0.1913 |  |

Solid lines distinguished different sets of survival assays, and different conditions were divided by dashed lines. Percent changes (%) and *p*–values were calculated within one experimental set. *p*–values were calculated with log-rank (Mantel-Cox method) test.

*^daf-2^* indicates the comparison of each experimental condition with *daf-2(e1370)* condition.

**TABLE S6** List of KIN-4 PDZ domain-interacting proteins identified from a yeast two-hybrid screen

| **Protein/ homolog** | **Number/types of clones** | **Description** | **Carbonyl-terminus** | **PDZ-binding motif** |
| --- | --- | --- | --- | --- |
| MIG-6/ Papilin | 24/14 | the extracellular matrix proteins papilin and lacunin | IEECQARCPSKF | Class I |
| DAF-18/ PTEN | 14/9 | a lipid phosphatase homologous to the human PTEN tumor suppresor | FSDSNFDQAIYL | Class II |
| CPR-1/ Cathepsin B | 6/7 | a cysteine protease of the cathepsin B-like cysteine protease family | IESAVVAGKAKV | Class II |
| ZTF-17/ ZFP42/YY2/YY1 | 4/2 | an ortholog of human ZFP42 (ZFP42 zinc finger protein), YY2 (YY2 transcription factor) and YY1 (YY1 transcription factor) | NFBDRHNNLNIS | - |
| MEL-11/ PP-1M | 3/3 | the vertebrate smooth muscle myosin-associated phosphatase regulatory subunit | GALVRVISKMTI | Class II |
| NID-1/ Nidogen-2 | 3/2 | the nidogen (entactin) protein, which is a member of the nidogen protein family and a structural component of basement membranes, believed to connect laminin and collagen IV networks together | CECPDNVKVKGC | - |
| K04H4.2 | 2/2 | predicted to be secreted, with an N-terminal chitin-binding peritrophin-A domain followed by up to 15 cysteine-rich domains | ANDDKSSSSVSV | Class II |
| F28H1.1 | 2/1 | uncharacterized | SPVDFGPWTSNL | Class I |
| NSY-7 | 2/1 | a protein with low similarity to homeodomain transcription factors | HHILNSLQETTA | Class I |
| F09E5.7/ PROSC | 1 | an ortholog of human PROSC (proline synthetase cotranscribed) | GNGGGGGFDGYI | - |
| F56C3.9/ BTBD19 | 1 | an ortholog of human BTBD19 (BTB domain containing 19) | FDWDHTPYSAYV | Class II |
| ZC247.1 | 1 | uncharacterized | ARQRVRNICTML | Class I |
| ERP-44.2/ ERP44 | 1 | an ortholog of human ERP44 (endoplasmic reticulum protein 44) | DKRYSILQKSEL | Class I |
| C06G3.6/ SQSTM1 | 1 | an ortholog of human SQSTM1 (sequestosome 1) | DFDVVEEEAKQS | - |
| C34F6.1/ TFPI2 | 1 | an ortholog of human TFPI2 (tissue factor pathway inhibitor 2) | ICCKEYKRRHRL | - |
| C39D10.7 | 1 | a large (1185-residue) protein with 11 chitin-binding peritrophin-A domains | VAPTTSSAYKSY | - |
| R12A1.3/ WFDC8/TFPI2 | 1 | an ortholog of human WFDC8 (WAP four-disulfide core domain 8) and TFPI2 (tissue factor pathway inhibitor 2) | MTSCGYKICYQY | Class II |
| SLT-1 | 1 | the sole *C. elegans* homolog of *Drosophila* Split, a secreted extracellular protein containing leucine-rich and EGF-like repeats that functions as a ligand for the Robo receptor | QCEPTKSVLSEK | - |
| RPS-0/ 40S ribosomal protein SA | 1 | a small ribosomal subunit SA protein | EEWANAPTQSNW | Class I |
| CYLD-1/ CYLD1 | 1 | an ortholog of human CYLD1 (Cylindromatosis) | STIISSIKNMMN | - |
| M03B6.3 | 1 | uncharacterized | MNSLNVVNFGDC | - |
| ACLY-1/ACLY | 1 | an ortholog of human ACLY (ATP citrate lyase) | SYIMPESNLVKF | Class II |
| F10G8.8/ MPRIP/TRIOBP | 1 | human MPRIP (myosin phosphatase Rho interacting protein) and TRIOBP (TRIO and F-actin binding protein) | SRKAEEKKSSPC | - |
| F55D12.2/ SESTD1 | 1 | human SESTD1 (SEC14 and spectrin domain containing 1) | PAIRLSKNESYL | Class I |
| BATH-44/ SPOP | 1 | a protein with a meprin-associated Traf homology (MATH) domain that may be involved in apoptosis | ASKRARMTETNL | Class I |
| R06F6.12 | 1 | uncharacterized | REKIIVLDEQSS | - |
| DPL-1/ Dp-1 | 1 | mammalian DP, which is the E2F-heterodimerization partner | QHPQPEEYDYFQ | - |
| UNC-52/ HSPG2 | 1 | perlecan, a protein orthologous to human basement membrane-specific heparan sulfate proteoglycan core protein (HSPG2) | TRTTTKLFGSWF | Class I |
| COH-4 | 1 | a member of the Rad21/Rec8-like family of cohesion proteins known as kleisins | IEDIDELAMADF | Class II |
| CPNA-1/ Copine domain protein | 1 | functions in striated muscle contraction | DDVAFVEYVTVL | Class I |
| NAS-23/ BMP1 | 1 | an astacin-like metalloprotease | RSEFIDENTADI | Class II |
| RBG-1/ RAB3GAP1 | 1 | human RAB3GAP1 (RAB3 GTPase activating protein catalytic subunit 1) and involved in amyloid fibril formation, autophagy and positive regulation of autophagosome assembly | ANDCNFSNSSYL | Class I |
| NHR-88/ NR5A1 | 1 | an ortholog of human NR5A1 (nuclear receptor subfamily 5 group A member 1) and NR5A2 (nuclear receptor subfamily 5 group A member 2) | TGVVHNQPFRFV | - |
| SPR-1/ REST corepressor 3 | 1 | the human corepressor CoREST that functions in HDAC-containing complexes to mediate transcriptional repression | RLADMEEEEDSI | Class III |
| ZK673.4 | 1 | a protein with a THAP or THAP-like domain | LVDAQLSTTSEV | Class I |
| SQST-2/ SQSTM1 | 1 | an ortholog of human SQSTM1 (sequestosome 1) | RYDIKREHYHWF | - |
| ZTF-1/ ZFAT | 1 | a zinc-finger transcription factor | DLEEVFNFGNVA | - |
| F53B3.3 | 1 | uncharacterized | QNPKNREATYHL | Class II |
| HIF-1/ HIF1 | 1 | an ortholog of the mammalian hypoxia-induced factor HIF1 | QPSSYSPFPMLS | - |
| CCAR-3/ CCAR1 | 1 | an ortholog of human CCAR1 (cell division cycle and apoptosis regulator 1) | DKDAEVVEESKE | - |
| T16G1.9/ EIF3K/BARD1 | 1 | an ortholog of human EIF3K (eukaryotic translation initiation factor 3 subunit K) and BARD1 (BRCA1 associated RING domain 1) | PHDLQEMGKQLL | - |

The PDZ domain of KIN-4 was used as bait for this yeast two-hybrid screen. We obtained 106 positive hits from 6.8 X 10^6^ screened clones. PDZ-binding motifs are located at C-termini of PDZ domain-binding proteins and classified into three classes based on the last three amino acid sequences: class I (serine/threonine-X (any amino acid)-ɸ (hydrophobic amino acid)), class II (ɸ-X- ɸ), and class III (glutamate/aspartate-X- ɸ). Candidates that contain PDZ-binding motifs were marked with these classes.

**TABLE S7** Analysis of lifespan assay results for secondary RNAi lifespan screen for candidates from yeast two-hybrid screen

| **Strain** | **Mean lifespan ±s.e.m. (days)** | **75th percentile** | **% change** | **Number of animals that died/total** | ***p* value vs. control** | **Figure in text** |
| --- | --- | --- | --- | --- | --- | --- |
| *rrf-3(pk1426)* control RNAi | 22.21±0.6 | 27 |  | 96/100 |  | Figure 4b and d |
| *rrf-3(pk1426)* *mig-6* RNAi | 22.69±0.8 | 27 | 2% | 69/100 | 0.1063 | Figure 4d |
| *rrf-3(pk1426)* *cpr-1* RNAi | 22.27±0.7 | 27 | 0% | 69/100 | 0.4782 |  |
| *rrf-3(pk1426)* *daf-18* RNAi | 19.97±0.4 | 24 | -10% | 77/96 | 0.0001 | Figure 4b |
| *rrf-3(pk1426); daf-2(e1370)* control RNAi | 52.08±1.7 | 63 | 134% | 65/100 | <0.0001 | Figure 4b and d |
| *rrf-3(pk1426); daf-2(e1370)* *mig-6* RNAi | 42.55±1.8 | 56 | -18% *^rrf-3; daf-2^* | 72/100 | 0.0001 *^rrf-3; daf-2^* | Figure 4d |
| *rrf-3(pk1426); daf-2(e1370)* *cpr-1* RNAi | 46.33±1.6 | 56 | -11% *^rrf-3; daf-2^* | 67/100 | 0.0005 *^rrf-3; daf-2^* |  |
| *rrf-3(pk1426); daf-2(e1370)* *daf-18* RNAi | 43.06±1.5 | 53 | -17% *^rrf-3; daf-2^* | 57/100 | <0.0001 *^rrf-3; daf-2^* | Figure 4b |
| *rrf-3(pk1426)* control RNAi | 24.2±0.9 | 31 |  | 69/75 |  |  |
| *rrf-3(pk1426)* *mig-6* RNAi | 21.55±0.7 | 28 | -11% | 90/100 | <0.0001 |  |
| *rrf-3(pk1426)* *cpr-1* RNAi | 23.68±0.8 | 28 | -2% | 60/100 | 0.3532 |  |
| *rrf-3(pk1426)* *daf-18* RNAi | 18.84±0.4 | 22 | -22% | 92/100 | 0.0169 |  |
| *rrf-3(pk1426); daf-2(e1370)* control RNAi | 47.41±1.8 | 61 | 96% | 69/75 | <0.0001 |  |
| *rrf-3(pk1426); daf-2(e1370)* *mig-6* RNAi | 39.64±1.4 | 49 | -16% *^rrf-3; daf-2^* | 80/88 | <0.0001 *^rrf-3; daf-2^* |  |
| *rrf-3(pk1426); daf-2(e1370)* *cpr-1* RNAi | 48.25±1.5 | 58 | 2% *^rrf-3; daf-2^* | 67/75 | 0.6554 *^rrf-3; daf-2^* |  |
| *rrf-3(pk1426); daf-2(e1370)* *daf-18* RNAi | 41.68±1.3 | 49 | -12% *^rrf-3; daf-2^* | 94/102 | <0.0001 *^rrf-3; daf-2^* |  |
| *rrf-3(pk1426); daf-2(e1370)* control RNAi | 53.55±1.6 | 62 |  | 44/91 |  |  |
| *rrf-3(pk1426); daf-2(e1370)* *mig-6* RNAi | 47.66±1.6 | 58 | -11% | 78/98 | 0.0291 |  |
| *rrf-3(pk1426); daf-2(e1370)* *cpr-1* RNAi | 49.84±1.2 | 58 | -7% | 77/100 | 0.0485 |  |
| *rrf-3(pk1426); daf-2(e1370)* *daf-18* RNAi | 36.25±0.8 | 40 | -32% | 63/100 | <0.0001 |  |
| *rrf-3(pk1426)* control RNAi | 26.34±1.0 | 32 |  | 81/100 |  |  |
| *rrf-3(pk1426)* *daf-16* RNAi | 18.76±0.6 | 23 | -29% | 51/100 | <0.0001 |  |
| *rrf-3(pk1426)* *coh-4* RNAi | 23.51±0.8 | 29 | -11% | 61/100 | 0.0223 |  |
| *rrf-3(pk1426)* *cpna-1* RNAi | 23.08±0.8 | 29 | -12% | 80/100 | 0.0022 |  |
| *rrf-3(pk1426)* *nsy-7* RNAi | 21.85±0.5 | 25 | -17% | 90/121 | <0.0001 |  |
| *rrf-3(pk1426)* *mel-11* RNAi | 19.52±0.4 | 22 | -26% | 92/113 | <0.0001 |  |
| *rrf-3(pk1426)* F28H1.1 RNAi | 21.89±0.6 | 25 | -17% | 94/117 | <0.0001 |  |
| *rrf-3(pk1426)* F56C3.9 RNAi | 21.56±0.6 | 25 | -18% | 85/120 | <0.0001 |  |
| *rrf-3(pk1426)* control RNAi | 25.82±0.9 | 33 |  | 73/120 |  |  |
| *rrf-3(pk1426)* *daf-16* RNAi | 19.22±0.6 | 23 | -26% | 84/120 | <0.0001 |  |
| *rrf-3(pk1426)* *mig-6* RNAi | 24.2±0.7 | 30 | -6% | 83/120 | 0.0361 |  |
| *rrf-3(pk1426)* *cpr-1* RNAi | 26.83±0.6 | 30 | 4% | 90/120 | 0.8301 |  |
| *rrf-3(pk1426)* *daf-18* RNAi | 20.63±0.4 | 23 | -20% | 82/120 | <0.0001 |  |
| *rrf-3(pk1426)* *coh-4* RNAi | 26.01±0.8 | 30 | 1% | 78/120 | 0.7917 |  |
| *rrf-3(pk1426)* *cpna-1* RNAi | 26.59±0.6 | 33 | 3% | 75/120 | 0.654 |  |
| *rrf-3(pk1426); nsy-7* RNAi | 22.62±0.8 | 27 | -12% | 73/120 | 0.0087 |  |
| *rrf-3(pk1426); daf-2(e1370)* control RNAi | 49.11±1.0 | 56 |  | 86/120 |  |  |
| *rrf-3(pk1426); daf-2(e1370)* *daf-16* RNAi | 22.67±0.5 | 28 | -54% | 88/120 | <0.0001 |  |
| *rrf-3(pk1426); daf-2(e1370)* *coh-4* RNAi | 48.54±1.0 | 56 | -1% | 94/120 | 0.4055 |  |
| *rrf-3(pk1426); daf-2(e1370)* *cpna-1* RNAi | 48.33±1.0 | 56 | -2% | 100/120 | 0.469 |  |
| *rrf-3(pk1426); daf-2(e1370)* *nsy-7* RNAi | 49.57±0.9 | 56 | 1% | 74/90 | 0.4795 |  |
| *rrf-3(pk1426)* control RNAi | 20.38±0.5 | 24 |  | 102/120 |  | Figure 4c |
| *rrf-3(pk1426)* *daf-16* RNAi | 16.07±0.4 | 19 | -21% | 110/120 | <0.0001 |  |
| *rrf-3(pk1426)* *mel-11* RNAi | 18.06±0.5 | 24 | -11% | 116/120 | 0.0036 | Figure 4c |
| *rrf-3(pk1426)* F28H1.1 RNAi | 20.35±0.5 | 24 | 0% | 110/120 | 0.8182 |  |
| *rrf-3(pk1426)* F56C3.9 RNAi | 19.77±0.5 | 24 | -3% | 118/120 | 0.6282 |  |
| *rrf-3(pk1426);* K04H4.2 RNAi | 19.59±0.5 | 24 | -4% | 114/120 | 0.3917 |  |
| *rrf-3(pk1426); bath-44* RNAi | 21.05±0.5 | 24 | 3% | 92/120 | 0.2759 |  |
| *rrf-3(pk1426); daf-2(e1370)* control RNAi | 44.77±0.9 | 53 |  | 103/124 |  | Figure 4c |
| *rrf-3(pk1426); daf-2(e1370)* *daf-16* RNAi | 21.11±0.5 | 23 | -53% | 93/120 | <0.0001 |  |
| *rrf-3(pk1426); daf-2(e1370)* *mel-11* RNAi | 37.69±0.8 | 44 | -16% | 117/122 | <0.0001 | Figure 4c |
| *rrf-3(pk1426); daf-2(e1370)* F28H1.1 RNAi | 44.42±1.0 | 50 | -1% | 70/90 | 0.657 |  |
| *rrf-3(pk1426); daf-2(e1370)* F56C3.9 RNAi | 43.42±1.0 | 50 | -3% | 78/90 | 0.1153 |  |
| *rrf-3(pk1426); daf-2(e1370)* K04H4.2 RNAi | 44.39±1.0 | 50 | -1% | 104/120 | 0.9602 |  |
| *rrf-3(pk1426)* control RNAi | 21.22±0.6 | 25 |  | 80/107 |  |  |
| *rrf-3(pk1426)* *daf-16* RNAi | 20.75±0.6 | 25 | -2% | 75/120 | 0.5489 |  |
| *rrf-3(pk1426)* *nsy-7* RNAi | 21.85±0.5 | 25 | 3% | 90/121 | 0.5227 |  |
| *rrf-3(pk1426); cpna-1* RNAi | 23.08±0.8 | 29 | 9% | 80/100 | 0.0003 |  |
| *rrf-3(pk1426); coh-4* RNAi | 22.07±0.9 | 29 | 4% | 58/100 | 0.0642 |  |
| *rrf-3(pk1426); daf-2(e1370)* control RNAi | 46.2±1.2 | 54 |  | 86/100 |  |  |
| *rrf-3(pk1426); daf-2(e1370)* *daf-16* RNAi | 23.53±0.5 | 28 | -49% | 73/100 | <0.0001 |  |
| *rrf-3(pk1426); daf-2(e1370)* *coh-4* RNAi | 49.11±1.4 | 59 | 6% | 79/100 | 0.0252 |  |
| *rrf-3(pk1426); daf-2(e1370)* *cpna-1* RNAi | 49.67±1.5 | 62 | 8% | 84/100 | 0.0039 |  |
| *rrf-3(pk1426); daf-2(e1370)* control RNAi | 49.36±1.4 | 58 |  | 81/100 |  |  |
| *rrf-3(pk1426); daf-2(e1370)* *daf-16* RNAi | 23.67±0.7 | 30 | -52% | 89/100 | <0.0001 |  |
| *rrf-3(pk1426); daf-2(e1370)* *nsy-7* RNAi | 47.91±1.4 | 58 | -3% | 68/94 | 0.3753 |  |
| *rrf-3(pk1426); daf-2(e1370)* *mel-11* RNAi | 39.88±1.0 | 47 | -19% | 88/97 | <0.0001 |  |
| *rrf-3(pk1426); daf-2(e1370)* F28H1.1 RNAi | 49.16±1.6 | 58 | 0% | 41/50 | 0.5440 |  |
| *rrf-3(pk1426)* control RNAi | 21.98±0.6 | 29 |  | 70/120 |  |  |
| *rrf-3(pk1426)* *daf-16* RNAi | 18.34±0.5 | 24 | -17% | 83/120 | <0.0001 |  |
| *rrf-3(pk1426)* K04H4.2 RNAi | 21.75±0.7 | 29 | -1% | 50/116 | 0.9832 |  |
| *rrf-3(pk1426); bath-44* RNAi | 21.5±0.6 | 24 | -2% | 84/120 | 0.5707 |  |
| *rrf-3(pk1426); daf-2(e1370)* control RNAi | 50.81±1.0 | 59 |  | 106/120 |  |  |
| *rrf-3(pk1426); daf-2(e1370)* *daf-16* RNAi | 23.75±0.6 | 27 | -53% | 89/105 | <0.0001 |  |
| *rrf-3(pk1426); daf-2(e1370)* F56C3.9 RNAi | 50.18±1.0 | 59 | -1% | 101/115 | 0.433 |  |
| *rrf-3(pk1426); daf-2(e1370)* K04H4.2 RNAi | 57.29±1.3 | 71 | 13% | 107/120 | <0.0001 |  |
| *rrf-3(pk1426); daf-2(e1370)* *bath-44* RNAi | 45.91±1.3 | 59 | -10% | 61/85 | 0.0721 |  |
| *rrf-3(pk1426)* control RNAi | 21.28±0.6 | 26 |  | 87/120 |  |  |
| *rrf-3(pk1426)* *daf-16* RNAi | 15.43±0.4 | 18 | -27% | 98/120 | <0.0001 |  |
| *rrf-3(pk1426)* *erp-44.2* RNAi | 20.24±0.6 | 23 | -5% | 73/90 | 0.1929 |  |
| *rrf-3(pk1426)* *acly-1* RNAi | 19.96±0.7 | 26 | -6% | 96/120 | 0.4643 |  |
| *rrf-3(pk1426);* F55D12.2 RNAi | 21.68±0.5 | 26 | 2% | 90/120 | 0.7779 |  |
| *rrf-3(pk1426); nas-23* RNAi | 19.35±0.6 | 23 | -9% | 95/118 | 0.0601 |  |
| *rrf-3(pk1426); daf-2(e1370)* control RNAi | 47.41±1.1 | 57 |  | 84/120 |  |  |
| *rrf-3(pk1426); daf-2(e1370)* *daf-16* RNAi | 19.88±0.5 | 27 | -58% | 76/108 | <0.0001 |  |
| *rrf-3(pk1426); daf-2(e1370)* *erp-44.2* RNAi | 47.13±1.1 | 53 | -1% | 100/120 | 0.7313 |  |
| *rrf-3(pk1426); daf-2(e1370)* *acly-1* RNAi | 46.57±1.1 | 53 | -2% | 95/120 | 0.9071 |  |
| *rrf-3(pk1426); daf-2(e1370)* F55D12.2 RNAi | 50.58±1.1 | 61 | 7% | 96/120 | 0.0020 |  |
| *rrf-3(pk1426); daf-2(e1370)* *nas-23* RNAi | 50.92±1.0 | 57 | 7% | 92/120 | 0.0057 |  |
| *rrf-3(pk1426)* control RNAi | 18.52±0.7 | 23 |  | 74/90 |  |  |
| *rrf-3(pk1426)* *daf-16* RNAi | 16.44±0.4 | 20 | -11% | 108/120 | 0.0001 |  |
| *rrf-3(pk1426)* *erp-44.2* RNAi | 19.06±0.5 | 23 | 3% | 100/120 | 0.9278 |  |
| *rrf-3(pk1426)* *acly-1* RNAi | 17.12±0.5 | 20 | -8% | 81/90 | 0.0054 |  |
| *rrf-3(pk1426);* F55D12.2 RNAi | 19.37±0.6 | 25 | 5% | 94/120 | 0.5566 |  |
| *rrf-3(pk1426); nas-23* RNAi | 18.6±0.6 | 23 | 0% | 78/90 | 0.5659 |  |
| *rrf-3(pk1426); daf-2(e1370)* control RNAi | 42.34±1.1 | 52 |  | 84/120 |  |  |
| *rrf-3(pk1426); daf-2(e1370)* *daf-16* RNAi | 21.7±0.4 | 25 | -49% | 104/120 | <0.0001 |  |
| *rrf-3(pk1426); daf-2(e1370)* *bath-44* RNAi | 38.6±0.8 | 44 | -9% | 94/120 | <0.0001 |  |
| *rrf-3(pk1426); daf-2(e1370)* *erp-44.2* RNAi | 40.09±0.9 | 46 | -5% | 91/120 | 0.0321 |  |
| *rrf-3(pk1426); daf-2(e1370)* *acly-1* RNAi | 40.82±1.0 | 46 | -4% | 86/120 | 0.2282 |  |
| *rrf-3(pk1426); daf-2(e1370)* F55D12.2 RNAi | 41.25±0.9 | 46 | -3% | 73/120 | 0.0556 |  |
| *rrf-3(pk1426); daf-2(e1370)* *nas-23* RNAi | 42.38±1.0 | 49 | 0% | 91/120 | 0.5290 |  |
| *rrf-3(pk1426)* control RNAi | 24.51±1.3 | 29 |  | 105/120 |  | Figure 4e |
| *rrf-3(pk1426)* *daf-16* RNAi | 19.01±0.5 | 23 | -22% | 103/120 | <0.0001 |  |
| *rrf-3(pk1426);* R12A1.3 RNAi | 25.97±1.1 | 29 | 6% | 97/120 | 0.0388 |  |
| *rrf-3(pk1426); rbg-1* RNAi | 25.68±0.7 | 33 | 5% | 96/120 | 0.0031 |  |
| *rrf-3(pk1426); rps-0* RNAi | 22.44±0.9 | 26 | -8% | 83/120 | 0.0484 | Figure 4e |
| *rrf-3(pk1426); spr-1* RNAi | 23.54±0.4 | 29 | -4% | 94/120 | 0.5304 |  |
| *rrf-3(pk1426); daf-2(e1370)* control RNAi | 50.01±1.4 | 60 |  | 51/60 |  | Figure 4e |
| *rrf-3(pk1426); daf-2(e1370)* R12A1.3 RNAi | 50.43±1.2 | 60 | 1% | 65/90 | 0.5899 |  |
| *rrf-3(pk1426); daf-2(e1370)* *rbg-1* RNAi | 49.71±1.6 | 60 | -1% | 71/89 | 0.4775 |  |
| *rrf-3(pk1426); daf-2(e1370)* *rps-0* RNAi | 63.30±2.0 | 73 | 27% | 45/60 | <0.0001 | Figure 4e |
| *rrf-3(pk1426); daf-2(e1370)* *spr-1* RNAi | 49.55±1.9 | 64 | -1% | 55/60 | 0.4466 |  |
| *rrf-3(pk1426)* control RNAi | 20.54±0.5 | 25 |  | 95/120 |  |  |
| *rrf-3(pk1426)* *daf-16* RNAi | 16.34±0.2 | 19 | -20% | 102/120 | <0.0001 |  |
| *rrf-3(pk1426);* R12A1.3 RNAi | 20.72±0.6 | 25 | 1% | 104/120 | 0.5760 |  |
| *rrf-3(pk1426); rbg-1* RNAi | 22.29±0.6 | 28 | 9% | 97/120 | 0.0131 |  |
| *rrf-3(pk1426); rps-0* RNAi | 18.65±0.6 | 22 | -9% | 78/120 | 0.0435 |  |
| *rrf-3(pk1426); spr-1* RNAi | 21.21±0.5 | 25 | 3% | 98/120 | 0.4548 |  |
| *rrf-3(pk1426); daf-2(e1370)* control RNAi | 51.5±1.0 | 59 |  | 81/90 |  |  |
| *rrf-3(pk1426); daf-2(e1370)* *daf-16* RNAi | 24.51±0.7 | 27 | -52% | 106/120 | <0.0001 |  |
| *rrf-3(pk1426); daf-2(e1370)* R12A1.3 RNAi | 45.48±0.9 | 53 | -12% | 94/120 | <0.0001 |  |
| *rrf-3(pk1426); daf-2(e1370)* *rbg-1* RNAi | 49.94±1.2 | 59 | -3% | 79/100 | 0.6710 |  |
| *rrf-3(pk1426); daf-2(e1370)* *rps-0* RNAi | 53.58±1.7 | 66 | 4% | 66/120 | 0.0006 |  |
| *rrf-3(pk1426); daf-2(e1370)* *spr-1* RNAi | 45.68±1.0 | 53 | -11% | 86/120 | <0.0001 |  |
| *rrf-3(pk1426)* control RNAi | 26.18±0.6 | 30 |  | 59/90 |  |  |
| *rrf-3(pk1426)* *daf-16* RNAi | 20.5±0.5 | 24 | -22% | 96/120 | <0.0001 |  |
| *rrf-3(pk1426); unc-52* RNAi | 24.55±0.5 | 30 | -6% | 86/120 | 0.0316 |  |
| *rrf-3(pk1426);* ZK673.4 RNAi | 26.89±0.9 | 30 | 3% | 87/120 | 0.8117 |  |
| *rrf-3(pk1426); daf-2(e1370)* control RNAi | 49.47±1.5 | 60 | 89% | 107/120 | <0.0001 |  |
| *rrf-3(pk1426); daf-2(e1370)* *daf-16* RNAi | 22.56±1.7 | 26 | -54% *^rrf-3; daf-2^* | 114/120 | <0.0001 *^rrf-3; daf-2^* |  |
| *rrf-3(pk1426); daf-2(e1370)* *unc-52* RNAi | 53.53±1.4 | 63 | 8% *^rrf-3; daf-2^* | 102/120 | 0.0189 *^rrf-3; daf-2^* |  |
| *rrf-3(pk1426); daf-2(e1370)* ZK673.4 RNAi | 53.51±0.6 | 63 | 8% *^rrf-3; daf-2^* | 105/120 | 0.1259 *^rrf-3; daf-2^* |  |
| *rrf-3(pk1426)* control RNAi | 19.17±0.4 | 21 |  | 78/90 |  |  |
| *rrf-3(pk1426)* *daf-16* RNAi | 14.66±0.3 | 19 | -24% | 114/120 | <0.0001 |  |
| *rrf-3(pk1426); unc-52* RNAi | 18.24±0.5 | 21 | -5% | 88/120 | 0.4896 |  |
| *rrf-3(pk1426);* ZK673.4 RNAi | 18.2±0.4 | 21 | -5% | 74/90 | 0.3058 |  |
| *rrf-3(pk1426); daf-2(e1370)* control RNAi | 42.55±1.0 | 48 | 122% | 100/120 | <0.0001 |  |
| *rrf-3(pk1426); daf-2(e1370)* *daf-16* RNAi | 17.07±0.4 | 20 | -60% *^rrf-3; daf-2^* | 101/120 | <0.0001 *^rrf-3; daf-2^* |  |
| *rrf-3(pk1426); daf-2(e1370)* *unc-52* RNAi | 41.37±1.6 | 50 | -3% *^rrf-3; daf-2^* | 94/120 | 0.3038 *^rrf-3; daf-2^* |  |
| *rrf-3(pk1426); daf-2(e1370)* ZK673.4 RNAi | 36.71±1.6 | 48 | -14% *^rrf-3; daf-2^* | 79/90 | 0.0764 *^rrf-3; daf-2^* |  |

Different lifespan assay sets were distinguished by solid lines, and different conditions were divided by dashed lines. All the lifespan assays were carried out by transferring L4 worms from control RNAi plates to corresponding RNAi plates. Percent changes (%) and *p*–values were calculated within one experimental set. *p*–values were calculated with log-rank (Mantel-Cox method) test.

*^rrf-3;^ ^daf-2^* indicates values obtained by comparing each experimental condition with *rrf-3(pk1426); daf-2(e1370)* control RNAi condition.

**Supplementary Experimental Procedures**

**Strains.** All strains were maintained at 20°C. Some strains were provided by the Caenorhabditis Genomics Center, which is funded by NIH Office of Research Infrastructure Programs (P40 OD010440). The following strains were used in this study. N2 wild-type, CF3152 *rrf-3(pk1426) II*, CF1814 *rrf-3(pk1426) II; daf-2(e1370) III*, IJ350 *kin-4(tm1049) IV* outcrossed 4 times with N2, CF1041 *daf-2(e1370) III* outcrossed 6 times to N2, IJ410 *daf-2(e1370) III; kin-4 (tm1049) IV*, IJ1386 *kin-4(nj170)* obtained by outcrossing IK2019 *kin-4(nj170)* 4 times with N2, IJ1803 *daf-2(e1370);* *kin-4(nj170)*, IJ642 *gipc-1(hc192) III; gipc-2(ok1172) IV*, IJ643 *daf-2(e1370) gipc-1(hc192) III; gipc-2(ok1172) IV*, IJ530 *yhEx121[odr-1p::RFP]*, IJ1066 *yhEx244[kin-4p::kin-4a::gfp; odr-1p::RFP]* line 1, IJ1067 *yhEx245[kin-4p::kin-4a::gfp; odr-1p::RFP]* line 2, IJ1266 *daf-2(e1370) III; kin-4(tm1049) IV; yhEx314[odr-1p::rfp]*, IJ1274 *daf-2(e1370) III; kin-4(tm1049) IV; yhEx322[kin-4p::kin-4a::gfp; odr-1p::rfp]*, IJ773 *daf-2(e1370) III; daf-18(nr2037) IV*, IJ1270 *daf-2(e1370) III; daf-18(nr2037) IV; yhEx318[odr-1p::rfp]*, IJ1265 *daf-2(e1370) III; daf-18(nr2037) IV; yhEx313[daf-18p::mCherry::daf-18 WT; odr-1p::rfp]*, IJ1267 *daf-2(e1370) III; daf-18(nr2037) IV; yhEx315[daf-18p::mCherry::daf-18 Δ4C; odr-1p::rfp]* line 1, IJ1268 *daf-2(e1370) III; daf-18(nr2037) IV; yhEx316[daf-18p::mCherry::daf-18 Δ4C; odr-1p::rfp]* line 2, IJ1269 *daf-2(e1370) III; daf-18(nr2037) IV; yhEx317[daf-18p::mCherry::daf-18 Δ12C; odr-1p::rfp]*, IJ1456 *daf-16(mu86) I; daf-2(e1370) III; muIs112[daf-16p::GFP::daf-16cDNA; odr-1p::RFP]*, IJ1458 *daf-16(mu86) I; daf-2(e1370) III; kin-4(tm1049); muIs112[daf-16p::GFP::daf-16cDNA; odr-1p::RFP]*, CF1553 *muIs84[sod-3p::gfp]*, CF1580 *daf-2(e1370) III; muIs84[sod-3p::GFP]*, IJ1445 *kin-4(tm1049) IV; muIs84[sod-3p::GFP]*, IJ1448 *daf-2(e1370) III; kin-4(tm1049) IV; muIs84[sod-3p::GFP]*, CF2553 *osm-5(p813) X*, IJ847 *kin-4(tm1049) IV; osm-5(p813) X*, IJ1664 *njEx683[kin-4p::kin-4::gfp; ges-1p::TagRFP]* outcrossed IK1847 *kin-4(tm1049) IV; njEx683[kin-4p::kin-4::gfp; ges-1p::TagRFP]* 4 times with N2, IJ1673 *daf-2(e1370) III; kin-4(tm1049) IV; yhEx446[rgef-1p::kin-4a::gfp; odr-1p::RFP]*, IJ1676 *daf-2(e1370) III; kin-4(tm1049) IV; yhEx449[lin-26p::kin-4a::gfp; odr-1p::RFP]*, IJ1693 *daf-2(e1370) III; kin-4(tm1049) IV; yhEx466[ges-1p::kin-4a::gfp; odr-1p::RFP]*, NL3321 *sid-1(pk3321) V*, IJ807 *daf-2(e1370) III; sid-1(pk3321) V*, TU3401 *sid-1(pk3321) V; uIs69[myo-2p::mCherry; unc-119::sid-1]*, IJ898 *daf-2(e1370) III; sid-1(pk3321) V; uIs69 [pCFJ90(Pmyo-2::mCherry); Punc-119::sid-1]*, WM27 *rde-1(ne219) V*, NR222 *rde-1(ne219) V; kzIs9[pKK1260(lin-26p::nls::GFP); pKK1253(lin-26p::rde-1); pRF6(rol-6(su1006)]*, NR350 *rde-1(ne219) V; kzIs20[pDM#715(hlh-1p::rde-1); pTG95(sur-5p::nls::GFP)]*, VP303 *rde-1(ne213) V; kbIs7[nhx-2p::rde-1; rol-6(su1006)]*, JM43 *rde-1(ne219); Is[wrt-2::RDE-1]*, IJ411 *daf-2(e1370) III; rde-1(ne219) V*, IJ415 *daf-2(e1370) III; rde-1(ne219) V; kzIs9[pKK1260(lin-26p::nls::GFP); pKK1253(lin-26p::rde-1); pRF6(rol-6(su1006)]*, IJ416 *daf-2(e1370) III; rde-1(ne219) V; kzIs20[pDM#715(hlh-1p::rde-1); pTG95(sur-5p::nls::GFP)]*, IJ417 *daf-2(e1370) III; rde-1(ne213) V; kbIs7[nhx-2p::rde-1; rol-6(su1006)]*, IJ418 *daf-2(e1370) III; rde-1(ne219); Is[wrt-2::RDE-1]*, CF1908 *eat-2(ad1116) II*, IJ1409 *eat-2(ad1116) II; kin-4(tm1049) IV*, IJ1696 *yhEx462[kin-4p::GFP; unc-122p::rfp]*, IJ1731 *yhEx497[mCherry::daf-18; kin-4a::gfp; ofm-1p::rfp]*, IJ1353 *daf-2(e1370) III; daf-18(nr2037) IV; yhIs84[daf-18p::mCherry::daf-18 Δ12C; odr-1p::rfp] #1* integrated IJ1269, IJ1555 *daf-2(e1370) III; daf-18(nr2037) IV; yhIs84[daf-18p::mCherry::daf-18 Δ12C; odr-1p::rfp] #1* outcrossed IJ1353 with IJ773 4 times, IJ1557 *daf-2(e1370) III; daf-18(nr2037) kin-4(tm1049) IV; yhIs84[daf-18p::mCherry::daf-18 Δ12C; odr-1p::rfp] #1.*

**Identification of PDZ proteins**

Using four different data sources, PDZ domain-containing *C. elegans* genes were assembled (S1 Table): i) 62 PDZ-containing genes from Wormbase (http://www.wormbase.org/) (R. Y. N. Lee et al., 2018), ii) 55 PDZ domain-containing genes from the previous studies (Tonikian et al., 2008) (Lenfant et al., 2010), iii) 46 PDZ domain-containing genes from precalculated data of Pfam (Finn et al., 2006) and iv) 64 PDZ domain-containing genes from manually conducted Pfam search. Genes that have HMM E-value greater than 0.01 or HMM aligned length shorter than 40 amino acids were subsequently excluded from our functional characterization. Eighty PDZ domain-containing *C. elegans* genes were finally selected.

**Lifespan assays.** Lifespan assays were conducted at 20^o^C as previously described, with some modifications (D. Lee et al., 2015). For RNAi lifespan screen, RNAi clones were obtained from the Julie Ahringer (Kamath & Ahringer, 2003) or Marc Vidal libraries (Rual et al., 2004). For the second RNAi lifespan screen for genes obtained from our yeast two-hybrid screen, RNAi clones that were not available from Ahringer or Vidal RNAi libraries, including those targeting *rbg-1*, *acly-1*, F28H1.1, ZK673.4, *mel-11*, K04H4.2, *erp-44.2*, *spr-1*, F55D12.2, *bath-44*, *nas-23*, F56C3.9, *unc-52*, *coh-4*, *cpna-1*, and *nsy-7*, were generated by using primers whose sequences were provided by Julie Ahringer library. RNAi bacteria cultured overnight at 37^o^C in 50 μg/mL ampicillin, and were seeded on ampicillin-containing NGM plates and incubated overnight at 37^o^C. Double stranded RNAs for RNAi were induced with 1 mM isopropyl β-D-1-thiogalactopyranoside (IPTG, Goldbio, MO) treatment for 24 hrs at room temperature or for 12 hrs at 37^o^C. To avoid potential developmental defects caused by RNAi, worms were fed with RNAi bacteria only during adulthood. Gravid adult worms were placed on control RNAi plates, and allowed to lay eggs for 24 hrs for synchronization. The worms were transferred onto each RNAi plate at L4 stage and subsequently onto each of new RNAi plates that contained 5-Fluoro-2′-deoxyuridine (FUdR, Sigma, MO) for the first 24 hrs and again after 48-72 hrs to prevent progeny production. If worms were grown on OP50 *E. coli* food, the worms were placed on FUdR-treated plates at young adult stage and transferred to new FUdR plates after 48 hrs. Dead worms were counted and animals that crawled off, contaminated by fungi, ruptured, bagged, or dug into agar were censored. Statistics were obtained by using online application of survival analysis 2 (OASIS 2: https://sbi.postech.ac.kr/oasis2/) (Han et al., 2016).

**Stress resistance assays.** Stress resistance assays were performed by following a previous report with some modifications (Seo et al., 2015). Worms laid eggs on OP50-seeded NGM plates for one day for synchronization. For oxidative stress resistance assays, young adults were transferred to new 5 μM FUdR-treated plates containing 7.5 mM tert-butyl hydroperoxide (t-BOOH, Sigma, MO). For heat stress resistance assays, synchronized young adults were incubated on plates without FUdR at 35^o^C. For pathogen resistance assays, *Pseudomonas aeruginosa* (PA14) were cultured overnight, and the PA14-seeded plates were incubated at 37^o^C for 24 hrs and subsequently at 25^o^C for 8-24 hrs to increase pathogenicity. Synchronized L4 stage worms were transferred onto the PA14-seeded plates containing 50 μM FUdR. The worms were transferred to new PA14-seeded plates again after 24-48 hrs from the first transfer to prevent reproduction. Dead worms were counted every 2 hrs for oxidative and heat stress resistance assays or three times a day for pathogen resistance assays. Statistics were calculated by using online application of survival analysis 2 (OASIS 2: https://sbi.postech.ac.kr/oasis2/) (Han et al., 2016).

**Generation of transgenic worms.** Transgenic worms were generated as previously described, with some modification (Artan et al., 2016). To generate *kin-4p::kin-4a::gfp*, the coding region (~4.5 kb) and a promoter region (~1 kb upstream sequences from start codon) of *kin-4a* were amplified using Pfu-X enzyme (Solgent, South Korea). To generate *kin-4::gfp* (pSN518), Counter-Selection BAC Modification kit (Gene Bridges, Heidelberg) was used to modify the fosmid WRM0635dF07 (Source BioScience, Nottingham). A GFP sequence from the vector pPD95.75 was inserted in front of the stop codon of the *kin-4* gene on the fosmid. For *daf-18p::mCherry::daf-18* construct, the coding region (~3 kb) and a promoter region (~1 kb) were amplified with the Pfu-X. Each coding region with the respective promoter was then inserted into pPD95.75 by using Infusion cloning (Takara, Japan). The constructs for *daf-18p::mCherry::daf-18 Δ4C* and *daf-18p::mCherry::daf-18 Δ12C* were generated by using primers that include respective deletion mutations. Each construct (50 ng/μL) and a co-injection marker (*odr-1p::rfp* or *ofm-1p::rfp*, 75 ng/μL) were microinjected into the gonads of day one adult worms.

**Yeast two-hybrid screen.** Yeast two-hybrid screen was performed by Panbionet (http://panbionet.com) following a previous report (Kim et al., 2008). To generate KIN-4 PDZ domain bait, the PDZ domain of KIN-4 (amino acid 1169th to 1257th of KIN-4 isoform a) with additional 10 amino acids at both sides, following previous reports (Belotti et al., 2013; Lenfant et al., 2010), were cloned into pGBKL vector, which contains the DNA-binding domain of GAL4. *C. elegans* cDNA prey library was generated by inserting cDNA fragments into pPC86 vector. Yeast strain that was used was PBN204, which expresses *URA3*, *ADE2*, and *lacZ*, and 6.8 x 10^6^ colonies were screened. To identify proteins interacting with the PDZ domain of KIN-4, yeast transformants were spread on a selection medium (SD) that did not include leucine, tryptophan or alanine (SD-LWA), and leucine, tryptophan or uracil (SD-LWU) to selectively grow yeast transformants yielding interactions between the prey and the bait proteins. To confirm the protein-protein interaction, three independent reporters with different types of GAL4-binding sites were employed. After confirmation, candidate prey proteins were identified by performing DNA sequencing and restriction enzyme digestion.

**Prediction of protein domains.** Amino acid sequences of KIN-4 and MAST2 were obtained from WormBase (http://www.wormbase.org/) (R. Y. N. Lee et al., 2018) and UniProt (http://www.uniprot.org/) (Magrane, 2011), respectively. Domains were predicted using InterPro (https://www.ebi.ac.uk/interpro/) (Quevillon et al., 2005) and drawn with DOG 2.0 program (Ren et al., 2009).

**Structure modeling and sequence alignment.** The structure of PDZ domain of KIN-4 was predicted based on that of MAST2 (PDB entry: 2KYL), using SWISS-MODEL (https://swissmodel.expasy.org/) (Schwede, Kopp, Guex, & Peitsch, 2003). The overlapping image between the PDZ domains of KIN-4 and MAST2 was generated by PyMOL program. The image that shows the predicted interaction between the PDZ domain of KIN-4 and the C-terminus of DAF-18 was generated by the Coot program (Emsley, Lohkamp, Scott, & Cowtan, 2010) based on 2KYL (Terrien et al., 2012).

**Generation of the phylogenic tree.** Amino acid sequences of *C. elegans* KIN-4, *D. melanogaster* Drop out, *X. laevis* MAST3, *M. musculus* MAST1/2/3, and *H. sapiens* MAST1/2/3 were obtained from UniProt (http://www.uniprot.org/) (Magrane, 2011). The phylogenic tree was drawn by using BLAST (https://blast.ncbi.nlm.nih.gov/Blast.cgi) (Altschul, Gish, Miller, Myers, & Lipman, 1990).

**Co-immunoprecipitation.** Co-immunoprecipitation was performed following a previous report (Seo et al., 2015) with some modifications. HEK293T cells cultured in 100 mm petri dishes were transfected with indicated plasmids. Cells were harvested 36 hrs after transfection, and then lysed in lysis buffer (20 mM Tris-HCl pH 7.4, 10 mM MgCl_2_, 2 mM ethylenediaminetetraacetic acid (EDTA), 10% glycerol, 0.01% Triton X-100, and 10 mM KCl) supplied with protease inhibitors (1 mM dithiothreitol (DTT), 0.5 mM phenylmethylsulfonyl fluoride (PMSF), 5 μg/ml leupeptin, 2 μg/ml pepstatin A, 5 μg/ml aprotin and 1 mM benzamindine) and phosphatase inhibitors (1 mM β-glycerophosphate, 1 mM Na_3_VO_4_, 5 mM NaF). Total 1 mg of cellular lysates were pre-cleared with mouse immunoglobulin G (IgG) and protein G/protein A-agarose beads (Calbiochem, CA) and then incubated with anti-FLAG antibody (2 μg/mg of lysates) overnight. Samples were washed four times in 1 ml of HNTG buffer (20 mM HEPES, pH 7.5, 150 mM NaCl, 0.1% Triton X100, 1% glycerol) as mentioned in a previous report (Valiente et al., 2005) supplied with protease inhibitors and phosphatase inhibitors. Proteins were then eluted from the bead by boiling at 95°C in Laemmli buffer (Bio-Rad, CA) containing 5% β-mercaptoethanol (Sigma, MO) for 5 min.

Protein samples were separated on 10% SDS-PAGE and transferred to nitrocellulose membrane (Whatman, NJ). Membranes were incubated with 7% skim milk in PBST (1% Tween 20 in PBS (pH 7.4)), followed by incubation with anti-HA (Santa Cruz, TX) or anti-FLAG (Sigma, MO) antibodies. Primary antibody incubation was followed by incubation with HRP-conjugated secondary antibody. Immunoreactive signals were visualized using an LAS4000 Image Reader (Fuji Film, Japan) after treatment with enhanced chemiluminescence (Thermo Fisher, MA) or SuperSignal™ West Femto Maximum Sensitivity Substrate (Thermo Fisher, MA).

**GST pull-down assay.** GST pull-down assays were performed following methods described in previous reports (Dev, Nishimune, Henley, & Nakanishi, 1999) with some modifications. To map the domain of KIN-4 that interacted with DAF-18, lysis buffer was used as described in the “Co-immunoprecipitation” and “Immunoblotting” sections. Protease inhibitors (2.5 mM β-glycerophosphate, 1 mM NaF, 1 mM DTT, and 1 mM PMSF) were freshly added before the usage of the buffer. The samples were sonicated six times using output 20%, 5 s sonication with 5 s interval, and incubated at 4^o^C for 1 hr with end-over-end rotation. NaCl (final concentration, 420 mM) was added after first 30 min incubation. The lysate was spun down for 15 min at 4^o^C with 15,700 g. After measuring total protein levels (1~2 mg) using Bradford assay, 30 μL of glutathione beads (GE healthcare, UK), which were washed three times with the lysis buffer before use, were added to the equal amount of each lysate. The mixture was incubated for 2~3 hrs at 4^o^C with end-over-end rotation, and the beads bound to GST-fused proteins were then washed with 1 mL of the lysis buffer three times. Cell lysate was prepared as described in the “Co-immunoprecipitation” and “Immunoblotting” sections. After measuring total protein levels (1~2 mg) using Bradford assay, cell lysates were aliquoted. To avoid non-specific binding, 200 μL of bovine serum albumin (BSA, 10 mg/mL) was added, and total volume was set to 1 mL. The mixture was incubated for 3~4 hrs at 4^o^C with end-over-end rotation, and then washed with 1 mL of the lysis buffer five times. Finally, 30 μL of the lysis buffer was added into each sample-containing tube, and the same amount of 2X Laemmli buffer was added to perform Western blot assays.

To determine the role of C-terminal DAF-18 in binding the PDZ domain of KIN-4, PTxE buffer composed of 1% Triton X-100 and 0.1 mM EDTA in PBS (pH 7.4) (Dev et al., 1999) with several protease inhibitors, 2.5 mM β-glycerophosphate, 1 mM NaF, 1 mM DTT and 1 mM PMSF, was used. Overall procedures were similar to methods described in the preceding paragraph except following; after GST-fused proteins were bound to beads, the beads were washed with PTx buffer (0.1% Triton X-100 in PBS pH 7.4). Cell lysate was prepared in PTxE buffer, and the cells were physically triturated with a syringe on ice and incubated at 4^o^C with end-over-end rotation for chemical lysis. The GST-fused proteins that bound to glutathione beads and the interacting proteins were washed with 1 mL of PTx buffer five times.

**Microscopy.** Microscopy experiments were executed as previously described with some modifications (Son et al., 2017). Worms were placed on a 2% agar pad and paralyzed with 2 mM levamisole treatment. Images were obtained by using Zeiss Axioscope A.1 microscope (ZEISS, Germany) with AxioCam HRc (ZEISS, Germany) camera. Confocal images were acquired by using Nikon Confocal microscopy (A1Rsi) at the Brain Research Core Facilities in Korea Brain Research Institute (KBRI) and using Olympus FLUOVIEW FV3000 Confocal Laser Scanning microscope (Olympus, Tokyo, Japan). Deconvolution images were obtained using Nikon ER (enhanced resolution) function in NIS Element (ver. 5.01) software program. Quantification was performed with imageJ program (Schindelin, Rueden, Hiner, & Eliceiri, 2015). For measuring DAF-16::GFP subcellular localization, semi-quantification was conducted blindly.

**Quantitative RT–PCR analysis.** Quantitative RT–PCR experiments were performed as previously described with some modifications (Son et al., 2017). Worms were allowed to develop at 20^o^C and collected at day 1 adult stage with M9 buffer. RNA was extracted from worm samples treated with RNAiso Plus (Takara, CA) and cDNA was synthesized using ImProm-II^TM^ reverse transcription system using random 6-mer primers (Promega, WI). Quantitative PCR was then performed with synthesized cDNA, primers that target respective genes, and SYBR Green Real Time-PCR master mixes (Thermo Fisher, MA) using StepOne^TM^ Real-Time PCR system (Thermo Fisher, MA). Results were analyzed with a comparative C_T_ method and *ama-1* mRNA levels were used for normalization. Following are primers used in this study.

*ama-1*-F: TGGAACTCTGGAGTCACACC

*ama-1*-R: CATCCTCCTTCATTGAACGG

*kin-4*-F: gtttcagaattccgattcggaaag

*kin-4*-R: GGCTCTGTTTCGACGCCATG

*sod-3*-F: CTATCTTCTGGACCAACTTGG

*sod-3*-R: GCAAGTTATCCAGGGAACCG

*dod-11*-F: GAACAAGCTGTTGAATACGTCAG

*dod-11*-R: GAAAGTAACCTTTGAATCCTTTG

*mtl-1*-F: GACTGCTGAAATTAAGAAATCATG

*mtl-1*-R: GTCTCCACTGCATTCACATTTGTC

**Supplementary References**

Altschul, S. F., Gish, W., Miller, W., Myers, E. W., & Lipman, D. J. (1990). Basic local alignment search tool. *J Mol Biol, 215*(3), 403-410. doi:10.1016/s0022-2836(05)80360-2

Artan, M., Jeong, D. E., Lee, D., Kim, Y. I., Son, H. G., Husain, Z., . . . Lee, S. J. (2016). Food-derived sensory cues modulate longevity via distinct neuroendocrine insulin-like peptides. *Genes Dev, 30*(9), 1047-1057. doi:10.1101/gad.279448.116

Belotti, E., Polanowska, J., Daulat, A. M., Audebert, S., Thome, V., Lissitzky, J. C., . . . Borg, J. P. (2013). The human PDZome: a gateway to PSD95-Disc large-zonula occludens (PDZ)-mediated functions. *Mol Cell Proteomics, 12*(9), 2587-2603. doi:10.1074/mcp.O112.021022

Dev, K. K., Nishimune, A., Henley, J. M., & Nakanishi, S. (1999). The protein kinase C alpha binding protein PICK1 interacts with short but not long form alternative splice variants of AMPA receptor subunits. *Neuropharmacology, 38*(5), 635-644.

Emsley, P., Lohkamp, B., Scott, W. G., & Cowtan, K. (2010). Features and development of Coot. *Acta Crystallogr D Biol Crystallogr, 66*(Pt 4), 486-501. doi:10.1107/s0907444910007493

Finn, R. D., Mistry, J., Schuster-Bockler, B., Griffiths-Jones, S., Hollich, V., Lassmann, T., . . . Bateman, A. (2006). Pfam: clans, web tools and services. Nucleic Acids Res, 34(Database issue), D247-251. doi:10.1093/nar/gkj149

Han, S. K., Lee, D., Lee, H., Kim, D., Son, H. G., Yang, J. S., . . . Kim, S. (2016). OASIS 2: online application for survival analysis 2 with features for the analysis of maximal lifespan and healthspan in aging research. *Oncotarget, 7*(35), 56147-56152. doi:10.18632/oncotarget.11269

Hwang, A. B., Ryu, E. A., Artan, M., Chang, H. W., Kabir, M. H., Nam, H. J., . . . Lee, S. J. (2014). Feedback regulation via AMPK and HIF-1 mediates ROS-dependent longevity in *Caenorhabditis elegans*. *Proc Natl Acad Sci U S A, 111*(42), E4458-4467. doi:10.1073/pnas.1411199111

Kamath, R. S., & Ahringer, J. (2003). Genome-wide RNAi screening in *Caenorhabditis elegans*. *Methods, 30*(4), 313-321.

Kim, J. E., Ryu, I., Kim, W. J., Song, O. K., Ryu, J., Kwon, M. Y., . . . Jang, S. K. (2008). Proline-rich transcript in brain protein induces stress granule formation. *Mol Cell Biol, 28*(2), 803-813. doi:10.1128/mcb.01226-07

Klenova, E., Chernukhin, I., Inoue, T., Shamsuddin, S., & Norton, J. (2002). Immunoprecipitation techniques for the analysis of transcription factor complexes. *Methods, 26*(3), 254-259. doi:10.1016/s1046-2023(02)00029-4

Lee, D., Jeong, D. E., Son, H. G., Yamaoka, Y., Kim, H., Seo, K., . . . Lee, S. J. (2015). SREBP and MDT-15 protect *C. elegans* from glucose-induced accelerated aging by preventing accumulation of saturated fat. *Genes Dev, 29*(23), 2490-2503. doi:10.1101/gad.266304.115

Lee, R. Y. N., Howe, K. L., Harris, T. W., Arnaboldi, V., Cain, S., Chan, J., . . . Sternberg, P. W. (2018). WormBase 2017: molting into a new stage. *Nucleic Acids Res, 46*(D1), D869-d874. doi:10.1093/nar/gkx998

Lenfant, N., Polanowska, J., Bamps, S., Omi, S., Borg, J. P., & Reboul, J. (2010). A genome-wide study of PDZ-domain interactions in *C. elegans* reveals a high frequency of non-canonical binding. *BMC Genomics, 11*, 671. doi:10.1186/1471-2164-11-671

Magrane, M. (2011). UniProt Knowledgebase: a hub of integrated protein data. *Database (Oxford), 2011*, bar009. doi:10.1093/database/bar009

Quevillon, E., Silventoinen, V., Pillai, S., Harte, N., Mulder, N., Apweiler, R., & Lopez, R. (2005). InterProScan: protein domains identifier. *Nucleic Acids Res, 33*(Web Server issue), W116-120. doi:10.1093/nar/gki442

Ren, J., Wen, L., Gao, X., Jin, C., Xue, Y., & Yao, X. (2009). DOG 1.0: illustrator of protein domain structures. *Cell Res, 19*(2), 271-273. doi:10.1038/cr.2009.6

Rual, J. F., Ceron, J., Koreth, J., Hao, T., Nicot, A. S., Hirozane-Kishikawa, T., . . . Vidal, M. (2004). Toward improving *Caenorhabditis elegans* phenome mapping with an ORFeome-based RNAi library. *Genome Res, 14*(10b), 2162-2168. doi:10.1101/gr.2505604

Schindelin, J., Rueden, C. T., Hiner, M. C., & Eliceiri, K. W. (2015). The ImageJ ecosystem: An open platform for biomedical image analysis. *Mol Reprod Dev, 82*(7-8), 518-529. doi:10.1002/mrd.22489

Schwede, T., Kopp, J., Guex, N., & Peitsch, M. C. (2003). SWISS-MODEL: An automated protein homology-modeling server. *Nucleic Acids Res, 31*(13), 3381-3385.

Seo, M., Seo, K., Hwang, W., Koo, H. J., Hahm, J. H., Yang, J. S., . . . Lee, S. J. (2015). RNA helicase HEL-1 promotes longevity by specifically activating DAF-16/FOXO transcription factor signaling in *Caenorhabditis elegans*. *Proc Natl Acad Sci U S A, 112*(31), E4246-4255. doi:10.1073/pnas.1505451112

Son, H. G., Seo, M., Ham, S., Hwang, W., Lee, D., An, S. W., . . . Roh, T. Y. (2017). RNA surveillance via nonsense-mediated mRNA decay is crucial for longevity in *daf-2*/insulin/IGF-1 mutant *C. elegans*. *8*, 14749. doi:10.1038/ncomms14749

Terrien, E., Chaffotte, A., Lafage, M., Khan, Z., Prehaud, C., Cordier, F., . . . Wolff, N. (2012). Interference with the PTEN-MAST2 interaction by a viral protein leads to cellular relocalization of PTEN. *Sci Signal, 5*(237), ra58. doi:10.1126/scisignal.2002941

Tonikian, R., Zhang, Y., Sazinsky, S. L., Currell, B., Yeh, J. H., Reva, B., . . . Sidhu, S. S. (2008). A specificity map for the PDZ domain family. PLoS Biol, 6(9), e239. doi:10.1371/journal.pbio.0060239

Valiente, M., Andres-Pons, A., Gomar, B., Torres, J., Gil, A., Tapparel, C., . . . Pulido, R. (2005). Binding of PTEN to specific PDZ domains contributes to PTEN protein stability and phosphorylation by microtubule-associated serine/threonine kinases. *J Biol Chem, 280*(32), 28936-28943. doi:10.1074/jbc.M504761200
