# Supplementary figures and images for: KIN‐4/MAST kinase promotes PTEN‐mediated longevity of Caenorhabditis elegans via binding through a PDZ domain
Source: Aging Cell. 2019 Feb 17;18(3):e12906. doi: 10.1111/acel.12906 (PMC6516182; doi:10.1111/acel.12906)

**Figure S1**

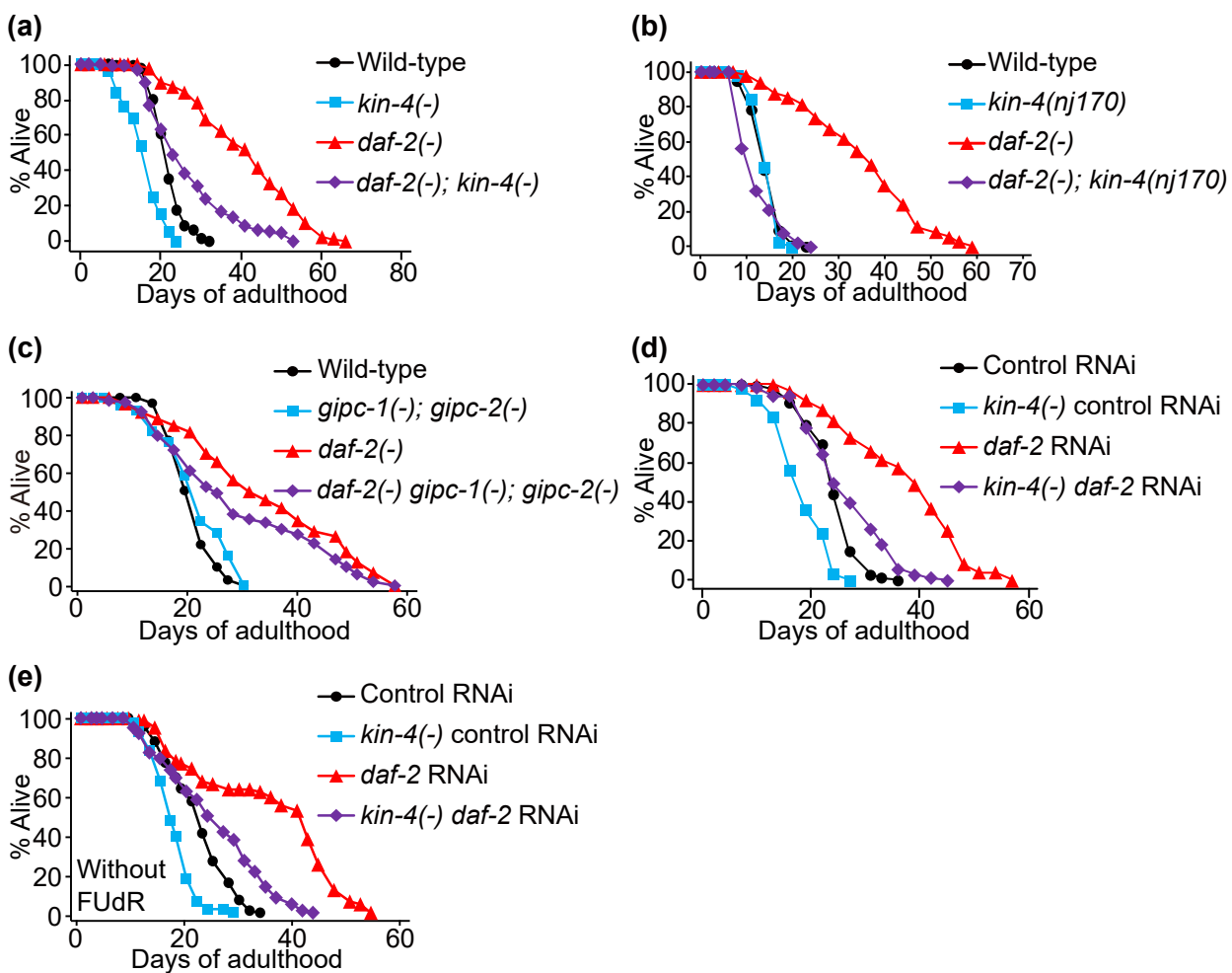

**Figure S2**

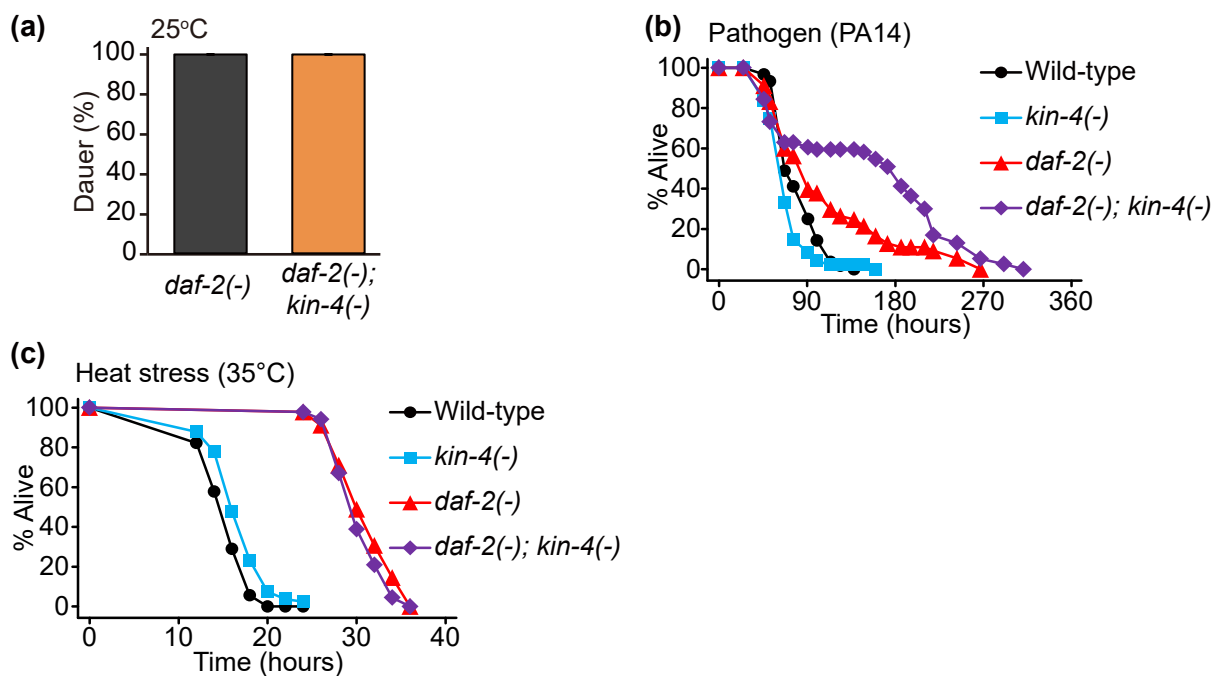

Figure S3

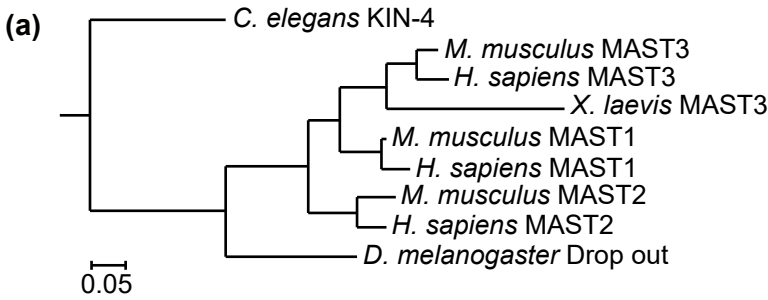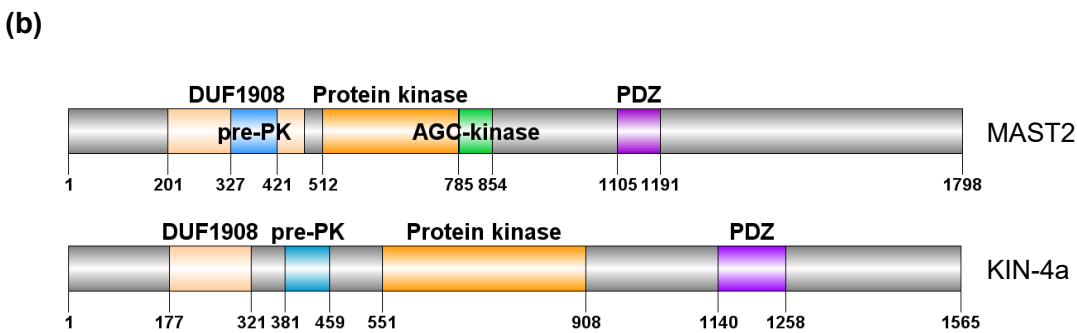

**Figure S4**

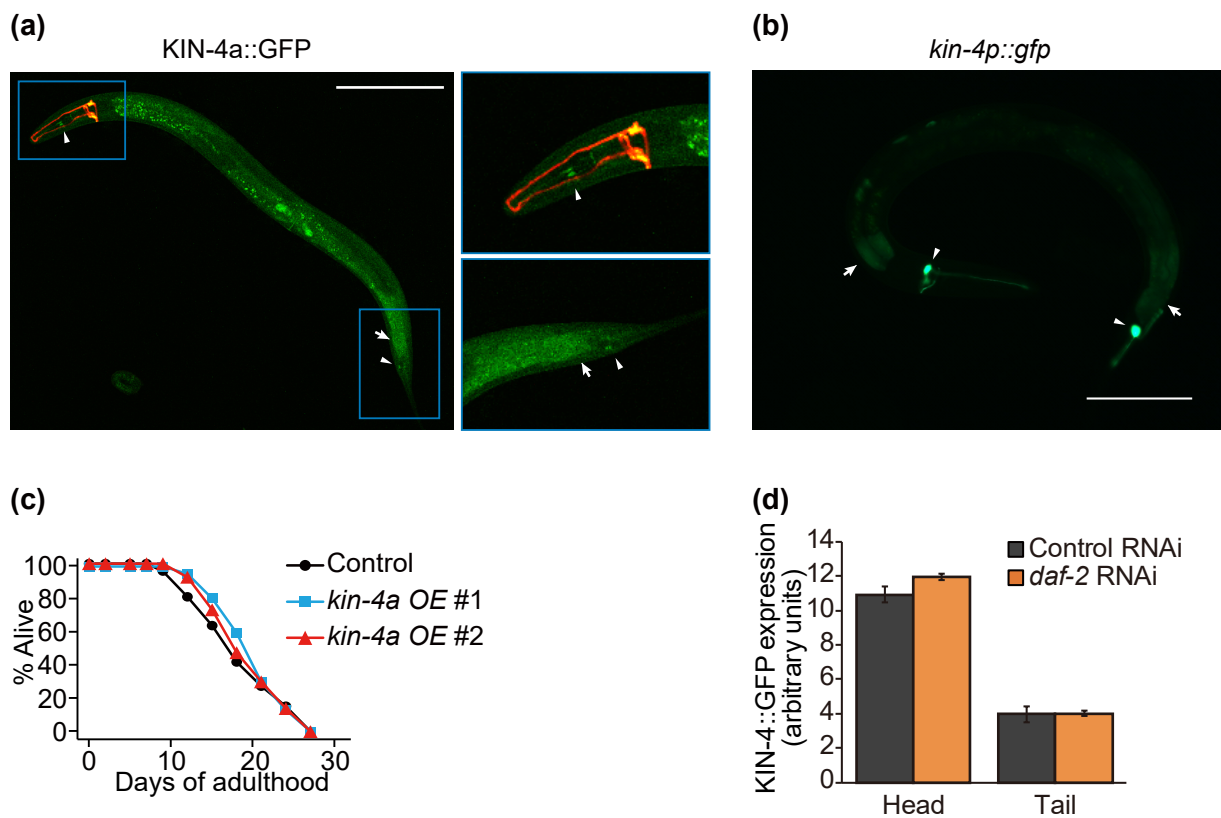

**Figure S5**

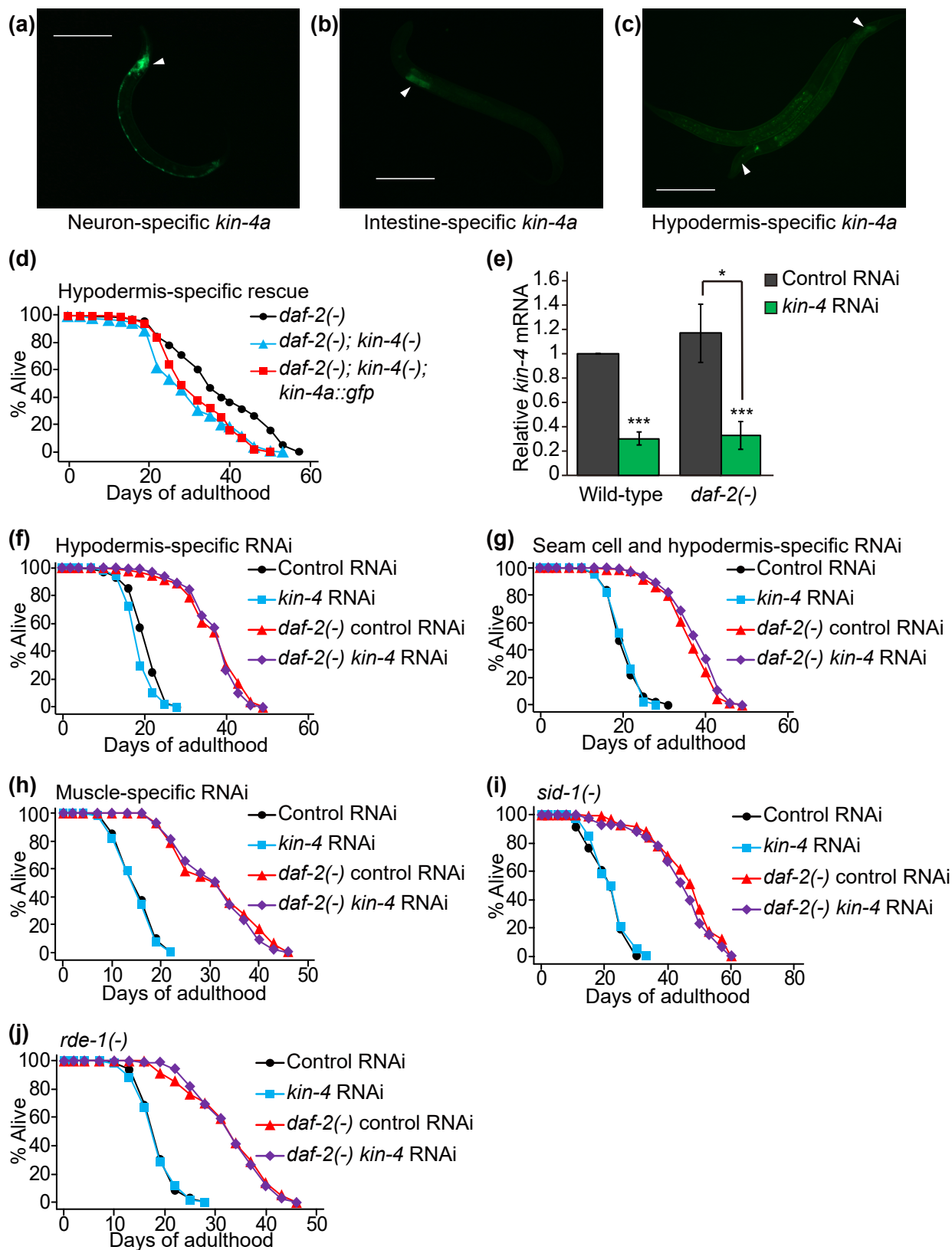

**Figure S6**

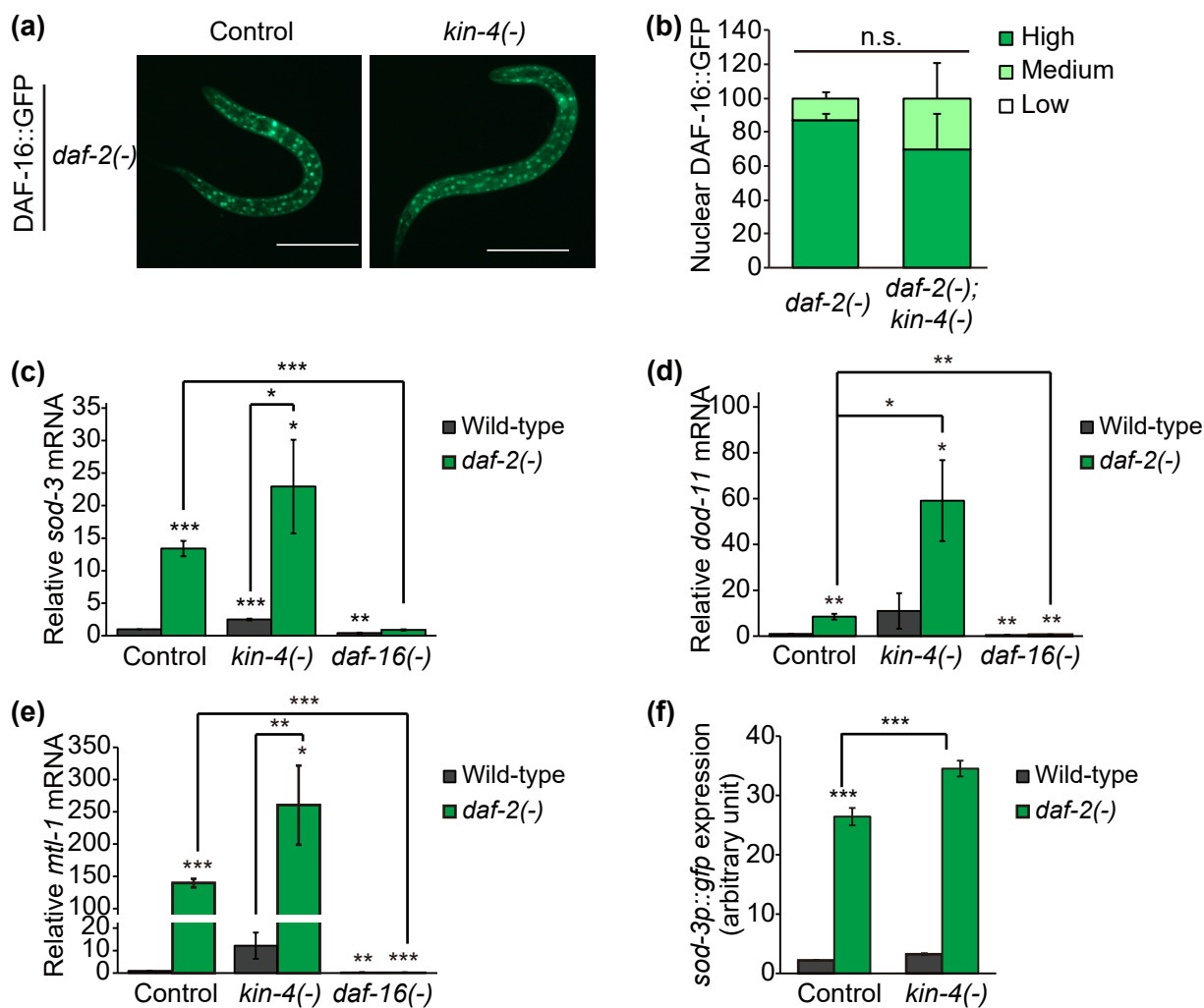

Figure S7

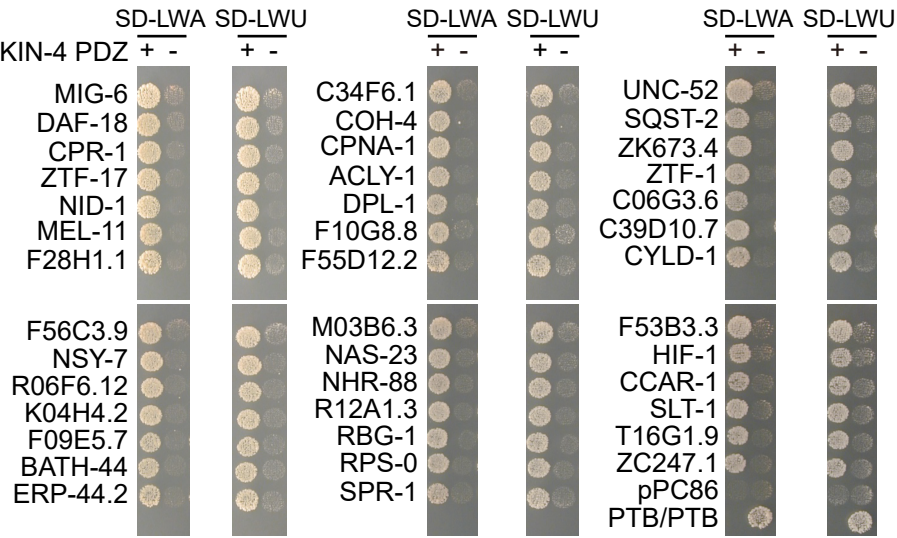

**Figure S8**

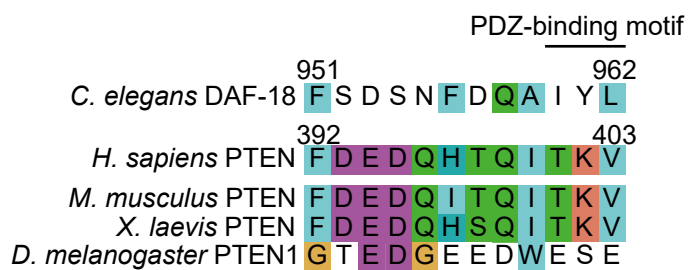

**Figure S9**

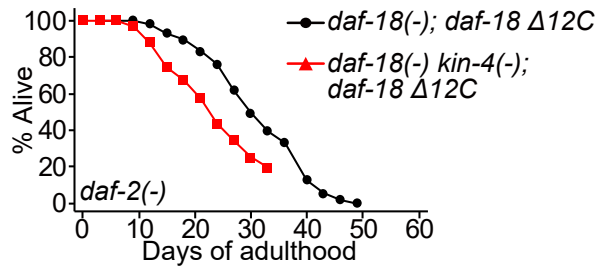

Supplement: Supplementary file 2 [file ACEL-18-e12906-s002.pdf]
